# Recent advances in the transesterification of β-keto esters

**DOI:** 10.1039/d1ra03513d

**Published:** 2021-07-02

**Authors:** M. C. Hennessy, T. P. O'Sullivan

**Affiliations:** School of Chemistry, University College Cork Cork Ireland tim.osullivan@ucc.ie; Analytical and Biological Chemistry Research Facility, University College Cork Cork Ireland; School of Pharmacy, University College Cork Cork Ireland

## Abstract

The ability to selectively transesterify β-keto esters is a useful transformation in organic synthesis. The increasing interest in transesterification for the synthesis of compounds of pharmaceutical importance, as well as for biodiesel production, has led to the development of a variety of different approaches. This article aims to summarise recent advances in the transesterifications of β-keto esters. Particular interest has been paid to methodologies with minimal environmental impact.

## Abstract 2

This review summarises the many advances in the selective transesterification of β-keto esters, with a particular focus on the past 30 years.

## Graphical abstract

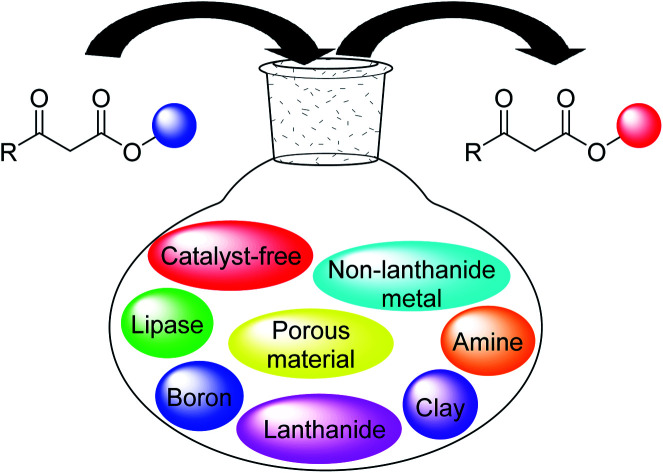

## 1. Introduction

**Fig. 1:**
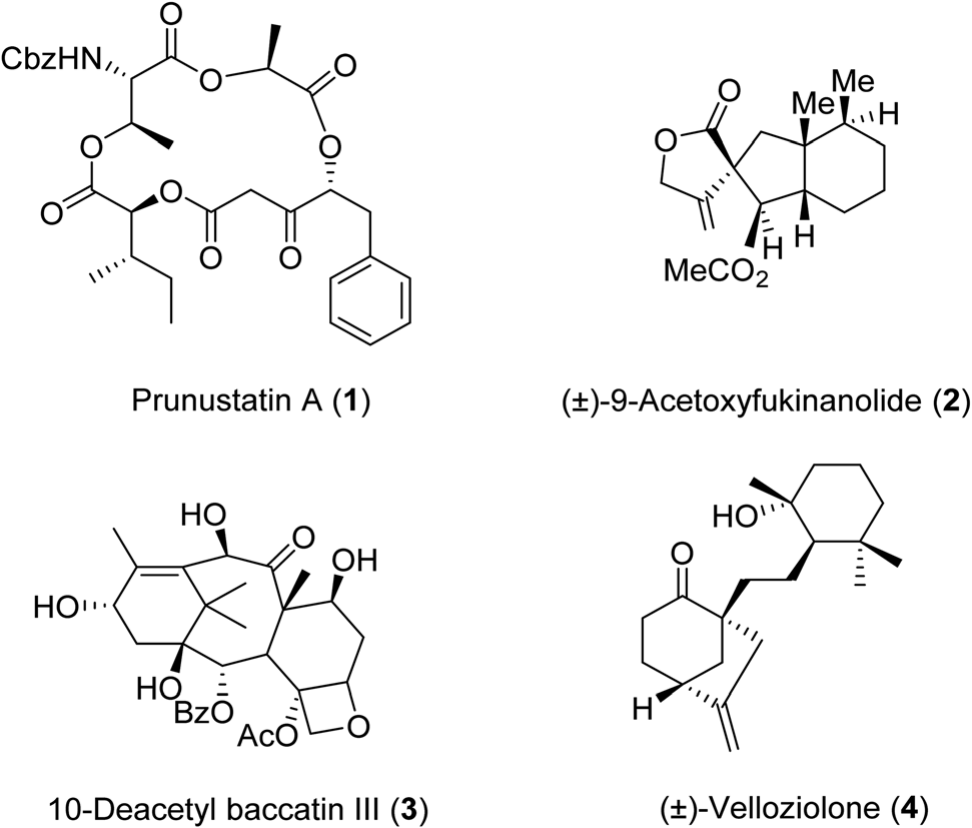
Examples of products synthesized *via* the transesterification of β-keto esters.

β-Keto esters contain both electrophilic and nucleophilic sites and so are important synthons in synthetic chemistry. They often constitute a core building block in complex medicinal compounds.^1^ β-Keto esters represent key intermediates in the synthesis of complex molecules such as paclitaxel, prunustatin A, (±)-9-acetoxyfukinanolide and (±)-velloziolone^2–5^ (Fig. 1).

Direct transesterification obviates the need to produce intermediate carboxylic acids, which often display poor solubility in organic solvents. Additionally, β-keto acids are unstable and tend to decarboxylate readily.^6^ A wide range of methyl and ethyl esters are commercially available and represent convenient starting points for elaboration *via* transesterification. The transesterification of β-keto esters has widespread application in agrochemicals where transesterification of vegetable oil with alcohols has long been a preferred method for the production of biodiesel.^7,8^ Transesterification is a convenient method for modifying both simple and elaborate esters and is commonplace in both research and industry.

**Scheme 1:**
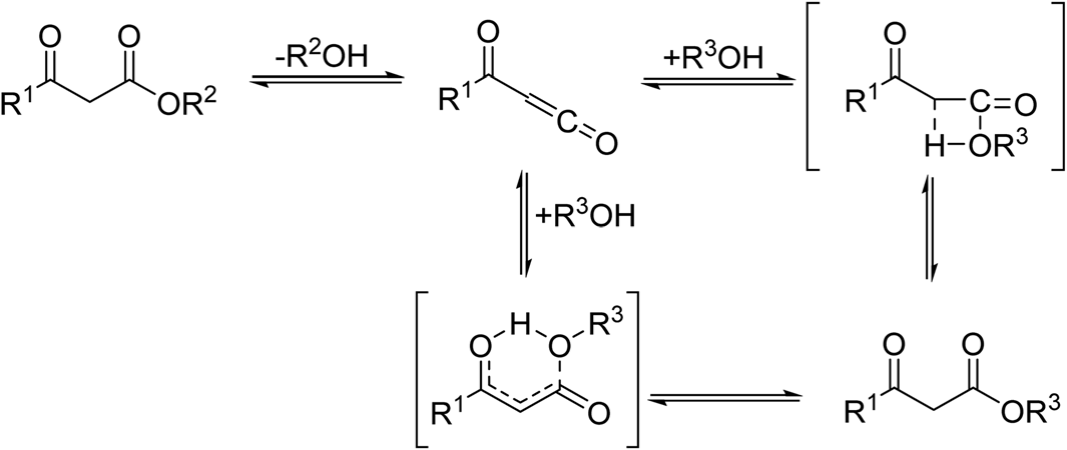
Transesterification *via* an acylketene intermediate.

β-Keto ester groups may be selectively transesterified over other esters such as simple, α-keto esters or γ-keto esters.^9^ Reactions which are selective for β-keto esters most likely proceed *via* an enol intermediate, as chelation between the two carbonyls to a catalyst heteroatom and the presence of an enolisable α-proton is important. Alternatively, the formation of an acylketene intermediate, as proposed by Lawrie and Campbell, may also occur (Scheme 1).^10^

A broad range of viable reaction conditions has been developed for the transesterification of β-keto esters.^11,12^ The kinetics of transesterifications are typically slow, so a catalyst is normally required. The literature contains many such examples including protic acids, Lewis acids, organic bases and enzymes as well as catalyst-free methodologies. These conditions are generally mild, allowing for transformations with broad functional group tolerance. In the case of acid-catalysed transesterifications, anhydrous conditions are usually required to prevent hydrolysis. Current trends in this area seek to exploit the advantages of both heterogeneous and homogeneous catalysts to achieve superior yields with good selectivity and, critically for industry, reduced environmental impact.

Transesterification has been the subject of much study since the publication of Otera's comprehensive review.^11^ There has not been a similar exercise in the interim period of 1993–2021, so it is timely to conduct a fresh survey of the literature. This current review has been subdivided based on the different catalytic approaches as follows:

(1) Boron catalysts

(2) Amine catalysts

(3) Lipase catalysts

(4) Non-lanthanide metal catalysts

(5) Lanthanide catalysts

(6) Clay catalysts

(7) Mesoporous and microporous material-based catalysts

(8) Catalyst-free methodologies

(9) Miscellaneous methods

## 2. Boron catalysts

With increasing demand for environmentally sustainable reagents and greater regulatory constraints on the chemical industry, boronic acids are an attractive option for large scale synthesis as they display low toxicity and decompose into boric acid. Boronic acids are efficient Lewis acid catalysts due to their vacant p orbital in the sp^2^ hybridised boron atom which can form reversible covalent bonds with carbonyl and hydroxyl groups.^13^

Boric acid is an environmentally benign catalyst which has been successfully utilised in several transformations.^14^ Kondaiah *et al.* have described its application in the transesterification of ethyl acetoacetate with a variety of primary and secondary alcohols (Table 1).^15^ The synthesis of β-keto esters using allylic (entry 9) and propargylic (entry 8) alcohols was successful with high yields obtained in a reaction time of just five hours. Tertiary alcohols were not compatible with these conditions.

**Table 1:** Boronic acid catalysis^15^

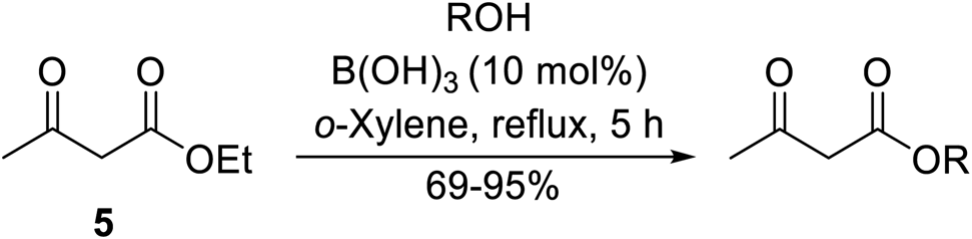			
Entry	R	Alcohol (equiv.)	Yield (%)
1	Bn	1.5	75
2	^*n*^Bu	2	92
3	4-CF_3_–C_6_H_4_CH_2_	1.5	95
4	^i^PrCH_2_	2	82
5	^*t*^BuCH_2_	2	80
6	^i^Bu	2	72
7	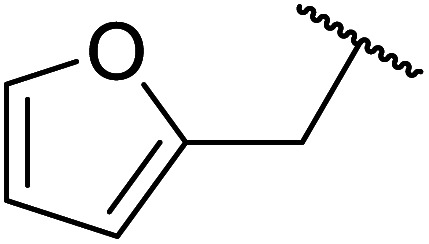	2	72
8	HC≡CCH_2_	2	71
9	H_2_C=CHCH_2_	2	69
10	Ph(CH_2_)_2_	1.5	77
11	Menthyl	1.5	92
12	Cy	2	95
13	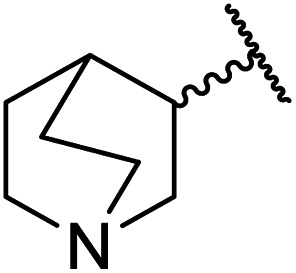	1.5	92

**Scheme 2:**
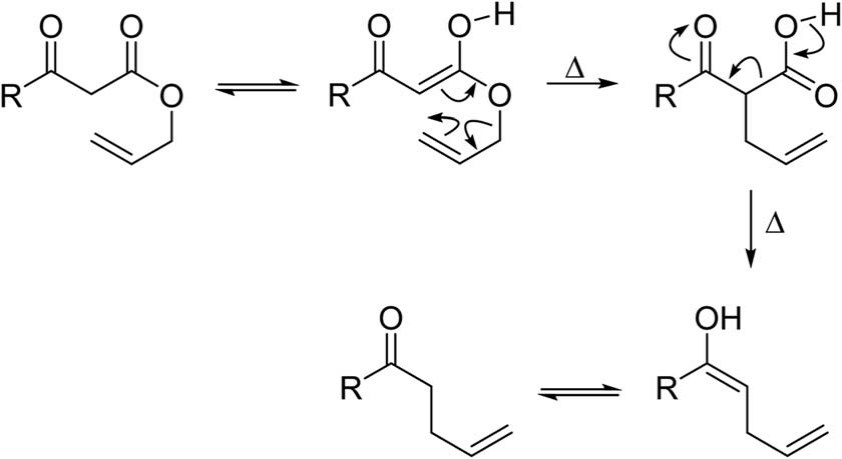
Carroll rearrangement and subsequent decarboxylation.

Wankhede and co-workers have reported a mild and environmentally benign protocol for the transesterification of β-keto ethylesters by employing methylboronic acid.^16^ 4 Å molecular sieves were also used to remove the liberated ethanol and help drive the reaction to completion. Methylboronic acid could be recovered by simple filtration and reused up to three times without significant loss of yield. Primary alcohols (Table 2 entries 1–3, 7–10), secondary alcohols (entries 4 and 6), and tertiary alcohols (entry 5) are compatible with this methodology generating the corresponding transesterified products in moderate to high yields. For benzylic alcohols, the nature of the ring substituents was important, with electron-donating substituents increasing reactivity (entries 3 and 9) and electron-withdrawing substituents decreasing reactivity (entry 10). Transesterification with α,β-unsaturated alcohols, such as cinnamyl alcohol (entries 7 and 8), is often challenging due to the propensity for Carroll rearrangement and subsequent decarboxylation (Scheme 2),^17–20^ so the ability of this catalyst to facilitate the transformation of these particular substrates is significant. Other types of esters, such as α-keto esters, γ-keto esters and simple esters, failed to react under these conditions. A likely explanation is that only β-keto esters allow for the formation of a 6-membered transition state by the coordination of boron with the two carbonyl oxygen atoms.

**Table 2:** Transesterification with methylboronic acid^16^

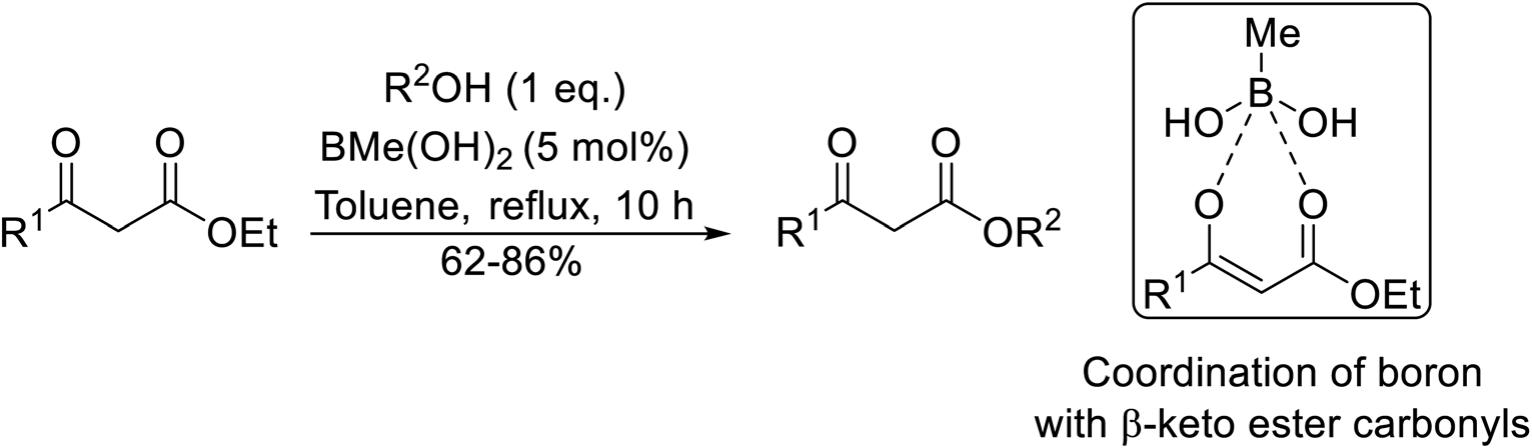			
Entry	R^1^	R^2^	Yield (%)
1	Me	^*n*^Dec	86
2	Me	Bn	75
3	Me	4-MeO–C_6_H_4_CH_2_	81
4	Me	(Ph)_2_CH	73
5	Me	1-Adamantanol	76
6	Me	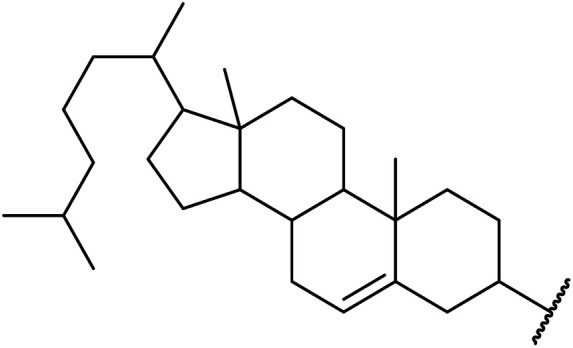	62
7	Ph	PhCH=CHCH_2_	63
8	Me	PhCH=CHCH_2_	81
9	Me	4-Me–C_6_H_4_CH_2_	78
10	Me	4-NO_2_–C_6_H_4_CH_2_	69

Arylboronic acids, particularly those with electron-withdrawing substituents on the aromatic ring, are often effective Lewis acid catalysts. For example, 3-nitrobenzeneboronic acid catalyses transesterifications in good to excellent yields with a 2.5 mol% catalyst loading.^21^ Cyclic, aromatic and open-chain β-keto esters were successfully transesterified with primary, secondary, tertiary, cyclic, allylic and benzylic alcohols (Table 3). Other nucleophiles, including thiols and amines, were similarly compatible allowing for transthioesterification (entry 12) and transamidation respectively (entries 13 and 16). Amino alcohols reacted preferentially at the amine (entry 16) due to the increased nucleophilicity of the nitrogen, whereas mercapto alcohols reacted at the hydroxyl (entry 15). Interestingly, diols underwent exclusive mono-transesterification (entry 14).

**Table 3:** Catalysis using 3-nitrobenzeneboronic acid^21^

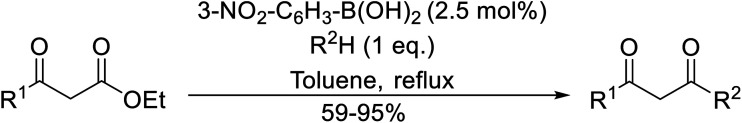				
Entry	R^1^	R^2^	Time (h)	Yield (%)
1	Me	^*n*^BuO	10	78
2	Me	^*n*^HexO	16	71
3	Me	^*n*^CH_3_(CH_2_)_9_O	14	74
4	Me	CyO	10	92
5	Ph	BnO	12	95
6	Me	4-Cl–C_6_H_4_CH_2_O	16	87
7	Me	2-Cl–C_6_H_4_CH_2_O	18	67
8	Ph	4-NO_2_–C_6_H_4_CH_2_O	16	75
9	Me	^*t*^BuO	19	59
10	Me	HC≡CCH_2_O	15	64
11	Me	PhCH_2_CH=CHO	12	89
12	Me	PhS	10	78
13	Me	PhNH	16	87
14	Me	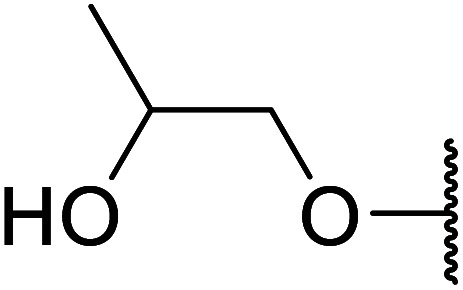	14	74
15	Me	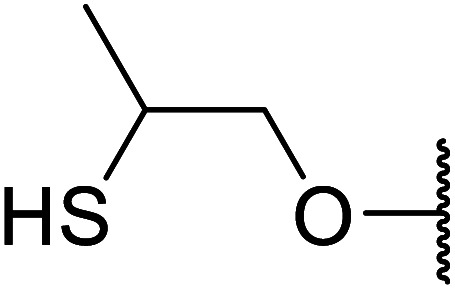	13	90
16	Me	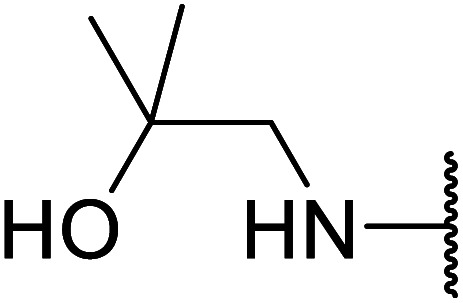	12	67

Liu and co-workers have exploited boron trifluoride diethyl etherate, a commercially available Lewis acid, for the transesterification of β-keto esters (Table 4).^22^ They also investigated other Lewis acid metal salts such as NiCl_2_, CuCl_2_, Bi(NO_3_)_5_, Fe_2_(SO_4_)_3_ and CoCl_2_, but these returned lower yields. Methyl acetoacetate and phenyl acetoacetate reacted readily with aliphatic (entries 1–8, 14), aromatic (entries 10 and 11) and allyl (entry 9) alcohols in good to excellent yields. When *n*-butanol was utilised, aromatic β-keto esters (entry 14) were found to undergo transesterification faster than aliphatic β-keto esters (entry 1). Other esters, such as a simple aliphatic ester (entry 16), γ-ketoester (entry 17), nitrile ester (entry 18) or unsaturated esters (entries 19 and 20), failed to react under these conditions even with an extended reaction time of 8 hours.

**Table 4:** Boron trifluoride diethyl etherate catalysis^22^

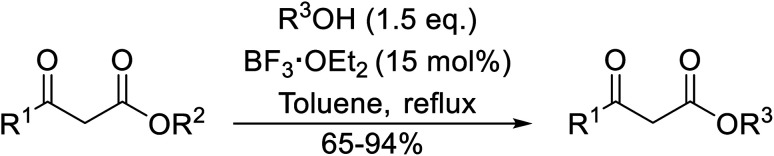					
Entry	R^1^	R^2^	R^3^	Time (h)	Yield (%)
1	Me	Me	^*n*^Bu	5.0	92
2	Me	Me	^*n*^Pr	4.5	94
3	Me	Me	^i^Bu	6.0	72
4	Me	Me	^i^Pr	5.0	87
5	Me	Me	^*sec*^Bu	5.5	81
6	Me	Me	^i^Pr(CH_2_)_2_	6.5	80
7	Me	Me	^*n*^Pen	6.5	86
8	Me	Me	^*n*^Oct	4.5	91
9	Me	Me	H_2_C=CHCH_2_	6.0	68
10	Me	Me	Bn	4.5	93
11	Me	Me	Ph(CH_2_)_2_	6.0	76
12	Me	Me	Cy	5.0	83
13	Me	Me	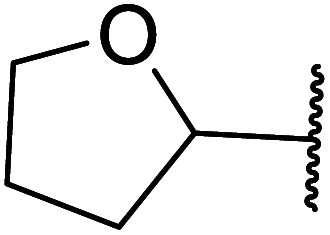	5.5	75
14	Ph	Me	^*n*^Bu	4.0	92
15	Ph	Me	Br(CH_2_)_2_	7.0	65
16	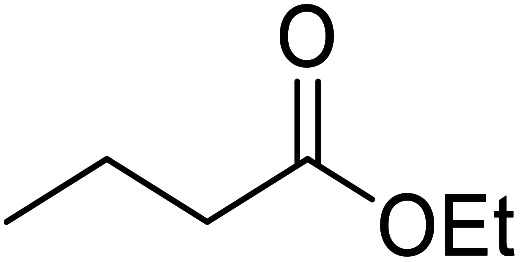		^*n*^Bu	8.0	n. r.
17	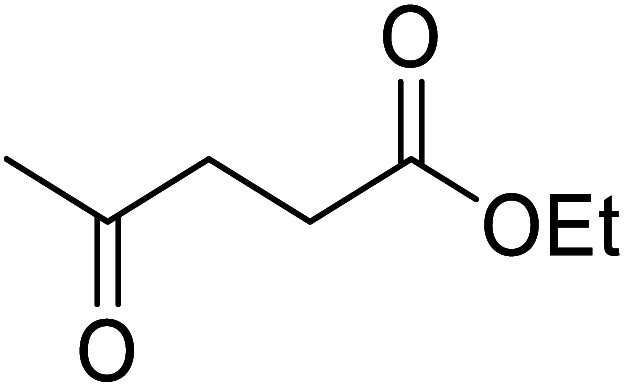		^*n*^Bu	8.0	n. r.
18	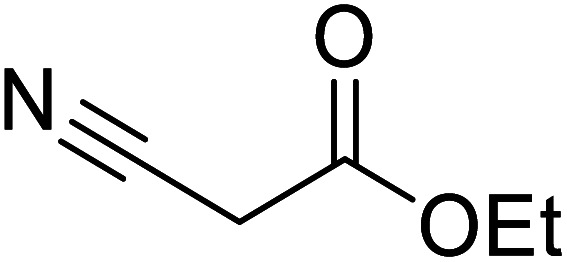		^*n*^Bu	8.0	n. r.
19	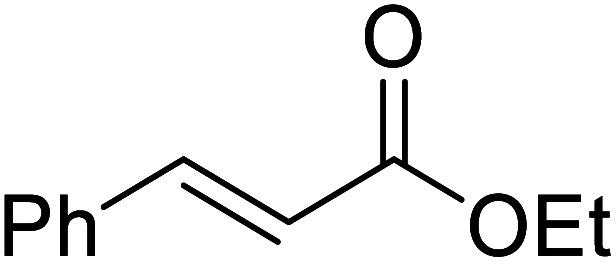		^*n*^Bu	8.0	n. r.
20	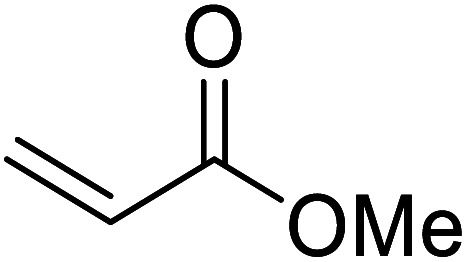		^*n*^Bu	8.0	n. r.

Madje *et al.* have demonstrated how a combination of borate and zirconia may serve as a selective and inexpensive catalyst combination to effect transesterifications under solvent-free conditions (Table 5).^23^ Conversion of methyl/ethyl keto esters to higher homologues proceeded smoothly. The less reactive *tert*-butyl alcohol was converted to the corresponding β-keto ester in moderate yields (entry 2). Unsaturated alcohols (*e.g.*, allyl alcohol) were also successfully transesterified (entry 3). The reaction was specific to β-keto esters as other esters, such as α-keto esters and γ-keto esters, did not react. The catalyst was recovered and reused at least three times without any appreciable loss of activity (entry 6). Transesterification of methyl acetoacetate with cyclohexanol in the presence of sulfated zirconia was lower yielding than with borate/zirconia (entry 1). The authors suggest that the enhanced activity of B_2_O_3_/ZrO_2_ is due to the three-coordinated boron, which has an empty orbital and pulls the electron cloud onto the oxygen of ZrO_2_. The negative charge of boron is diffused into the B_2_O_3_ bulk *via* resonance between the lone pair of oxygen and the empty orbital of boron.

**Table 5:** Borate/zirconia-catalysed transesterification of methyl and ethyl β-keto esters^23^

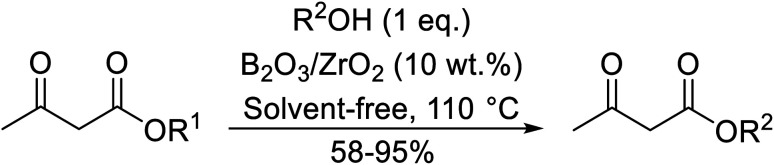				
Entry	R^1^	R^2^	Time (h)	Yield (%)
1	Me	Cy	3	85 (78^a^)
2	Me	^*t*^Bu	5	58
3	Me	H_2_C=CHCH_2_	4	70
4	Et	(−)-Menthyl	3	95
5	Me	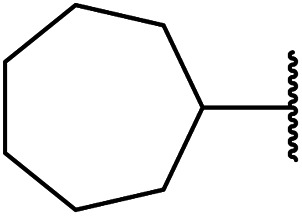	3.5	89
6	Me	Bn	4	84 (82^b^)
7	Me	HO(CH_2_)_3_	3.5	87^c^
8	Et	HO(CH_2_)_3_	4	92^c^
9	Me	^*sec*^Bu	3	81
10	Me	^*n*^Pr	2.5	88
11	Me	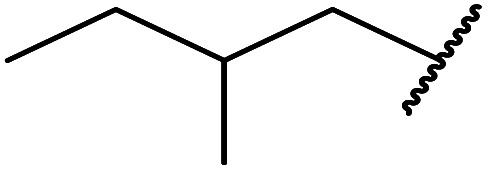	3	78
12	Me	^*n*^Hex	3	86

a Yield with sulfated zirconia.

b Yield after third cycle.

c Two equivalents of ester used.

## 3. Amine catalysts

Amines are among some of the oldest reported transesterification catalysts. In recent years, there has been renewed interest in the development of heterogeneous amine catalysts that possess improved toxicity profiles and the potential to be recovered and reused.

The application of 4-DMAP as a catalyst for the transesterification of β-keto esters was first described by Taber *et al.* in 1985 (Table 6).^24^ Taber compared the use of an excess of either the alcohol (entry 4) or the β-keto ethyl ester (entry 3) with a 30 mol% loading of 4-DMAP in an attempt to force the reactions to completion. Employing an excess of the ester was found to be more effective overall. Non-enolisable β-keto esters (entry 2) and tertiary alcohols (entry 5) were incompatible with these conditions.

**Table 6:** 4-DMAP catalysis in toluene^24^

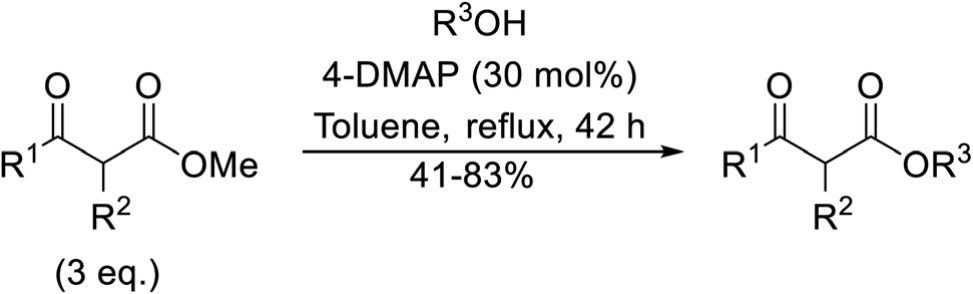				
Entry	R^1^	R^2^	R^3^	Yield (%)
1	Me	H	(−)-Menthyl	83
2	Me	(H_2_C=CH)_2_	(−)-Menthyl	n. r.
3	^*n*^Pen	H	^*n*^Bu	74^a^
4	^*n*^Pen	H	^*n*^Bu	41^b^
5	^*n*^Pen	H	^*t*^Bu	n. r.
6	Me	H	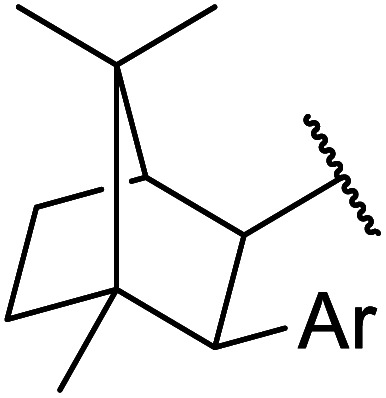	71
7	Me	Me	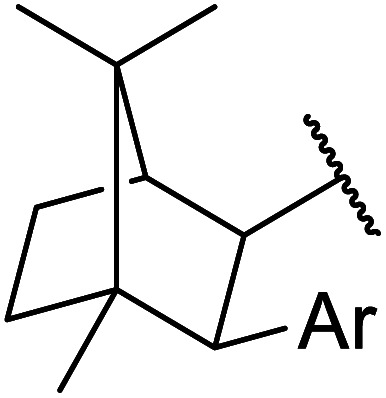	55

a Excess ester.

b Excess alcohol.

Building upon Taber's methodology, Gilbert and Kelly re-examined the 4-DMAP-catalysed transesterification of allylic alcohols in the presence of 4 Å molecular sieves.^25^ Good yields were recorded using stoichiometric amounts of the reagents and lower reaction temperatures (Table 7). Allyl alcohol (entries 1 and 6) furnished poor yields due to absorption into the molecular sieves. However, this problem was easily addressed by replacing the 4 Å sieves with 3 Å sieves (entry 2). Similar to Taber's findings, both tertiary alcohols and non-enolisable ketones proved unreactive. This may, in part, be due to a steric effect as ethyl 2-methyl-3-oxobutanoate (entries 6–9) required a much longer reaction time of 36 hours than ethyl acetoacetate (entry 1).

**Table 7:** Catalysis using 4-DMAP and 4 Å molecular sieves^25^

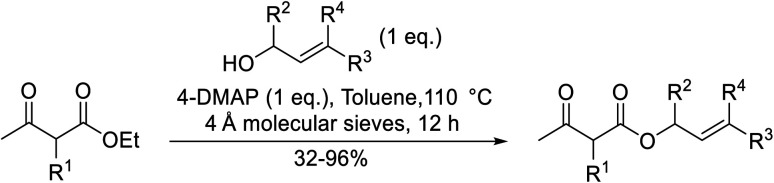					
Entry	R^1^	R^2^	R^3^	R^4^	Yield (%)
1	H	H	H	H	45
2	H	H	H	H	96^a^
3	H	H	Me	H	94
4	H	H	Me	Me	86
5	H	Me	H	H	84
6	Me	H	H	H	32^b^
7	Me	H	Me	H	83^b^
8	Me	H	Me	Me	84^b^
9	Me	Me	H	H	54^b^

a 3 Å molecular sieves, 36 hours reaction time.

b 36 hours reaction time.

Christoffers and Önal further improved upon Taber's methodology by replacing toluene with cyclohexane as the solvent.^26^ Importantly, these conversions proceeded with nearly stoichiometric amounts of starting materials and 5 mol% of 4-DMAP (Table 8). The methanol liberated from the reaction forms an azeotrope with cyclohexane in the vapour phase, allowing for the methanol to be removed by azeotropic distillation. A challenging double transesterification involving 2.2 equivalents of menthol proceeded in high yields (entry 8). By contrast, sterically crowded β-keto esters (entries 5 and 6) afforded lower yields.

**Table 8:** 4-DMAP catalysis in cyclohexane^26^

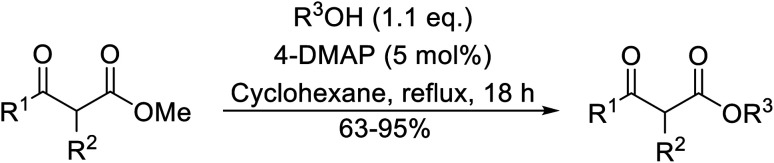			
Entry	β-Keto ester	R^3^	Yield (%)
1	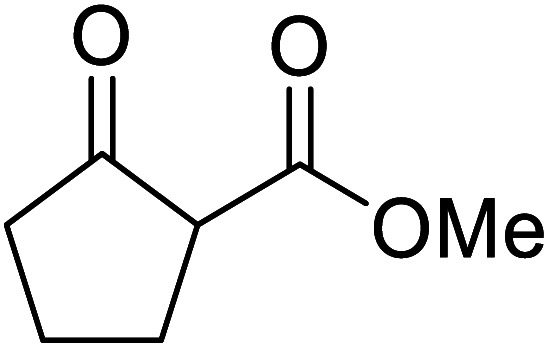	(−)-Menthyl	95
2	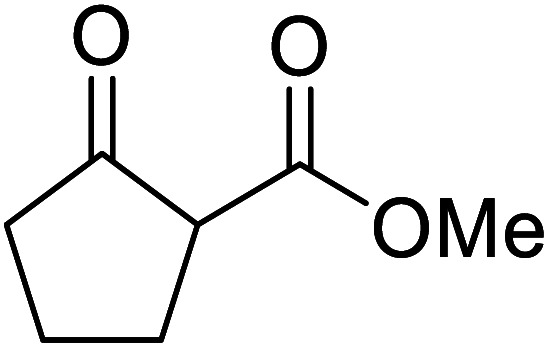	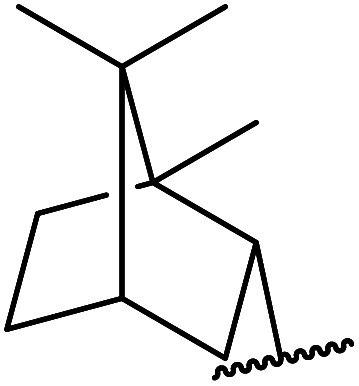	92
3	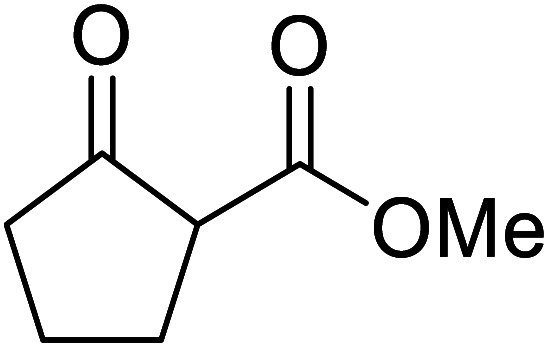	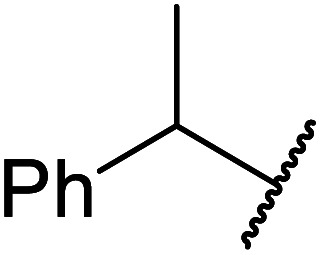	91
4	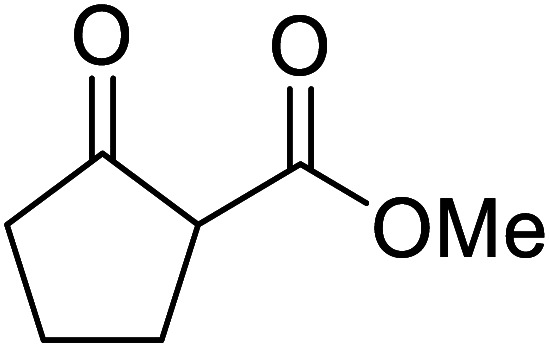	Bn	89
5	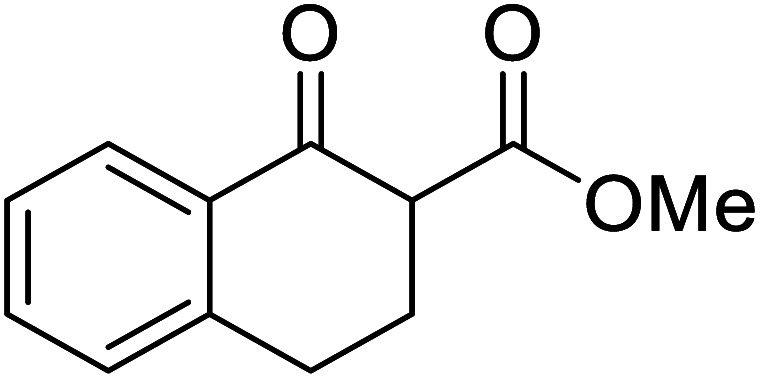	(−)-Menthyl	63
6	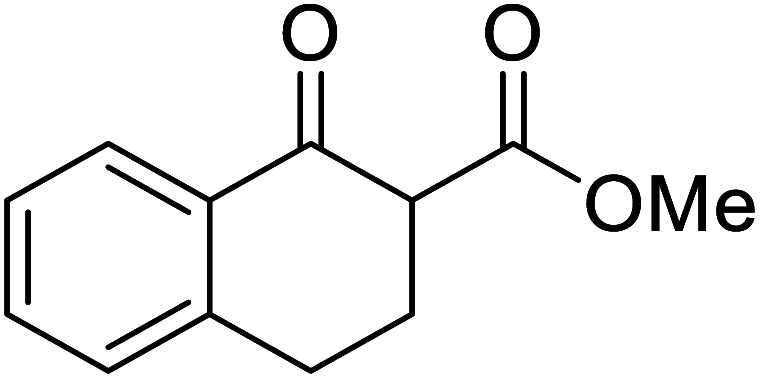	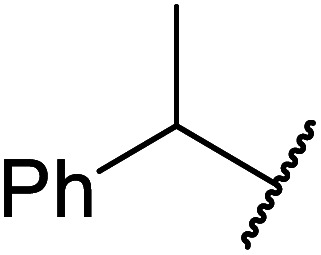	72
7	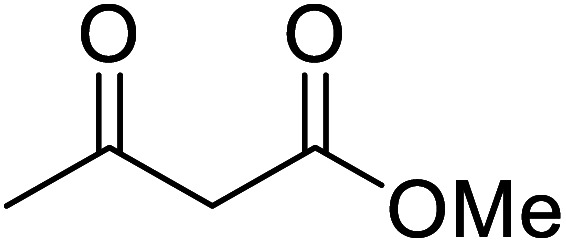	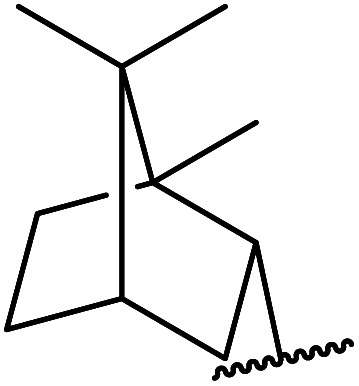	89
8	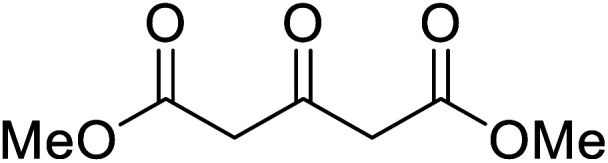	(−)-Menthyl	92^a^

a Di-transesterification product.

An interesting application of this approach is described by Shinoda and Osuka. They explored the chemoselective transesterification of the photosynthetic pigment, methyl pheophorbide-α (6), with complex alcohols (Table 9).^27^ A combination of 2-chloro-1-methylpyridinium iodide (CMPI) and 4-DMAP was a prerequisite for successful conversion. 4-DMAP could be substituted with other bases, such as trimethylamine, affording similar results. Of all the alcohols tested, sterols exhibited the highest reactivity (entries 1 and 2). Esterification of ethylene glycol proceeded at both hydroxyls to generate a pheophorbide dimer (entry 5).

**Table 9:** 4-DMAP and CMPI catalysis^27^

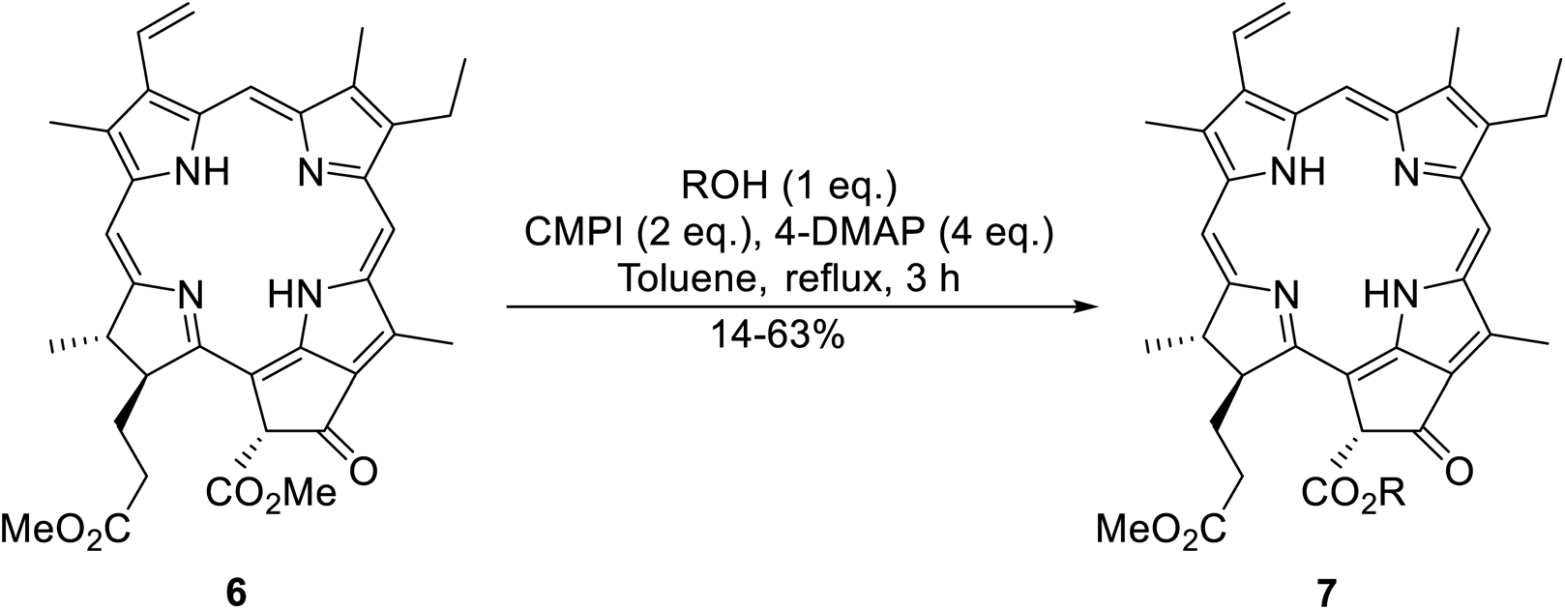		
Entry	R	Yield (%)
1	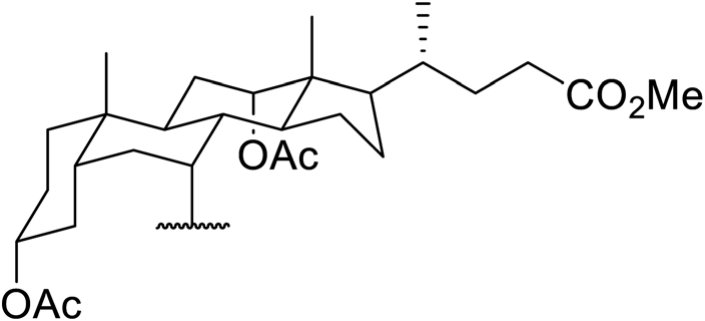	63
2	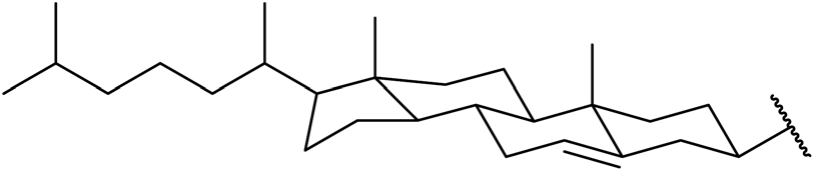	57
3	Et	41
4	^*n*^CH_3_(CH_2_)_17_	38
5	HO(CH_2_)_2_–	14^a^
6	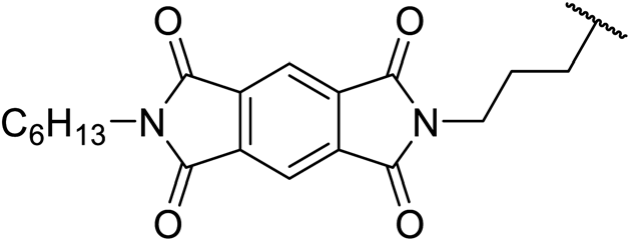	33
7	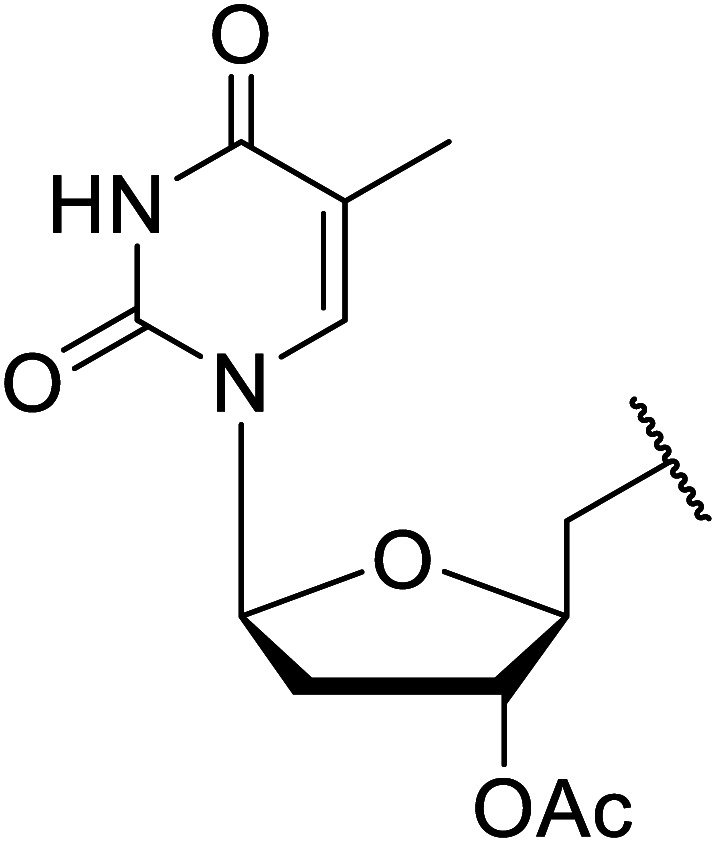	41
8	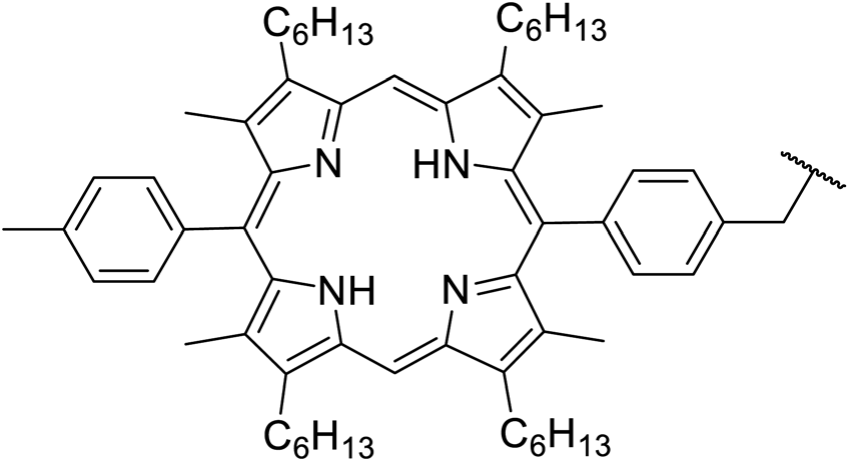	46

a Pheophorbide dimer.

**Scheme 3:**
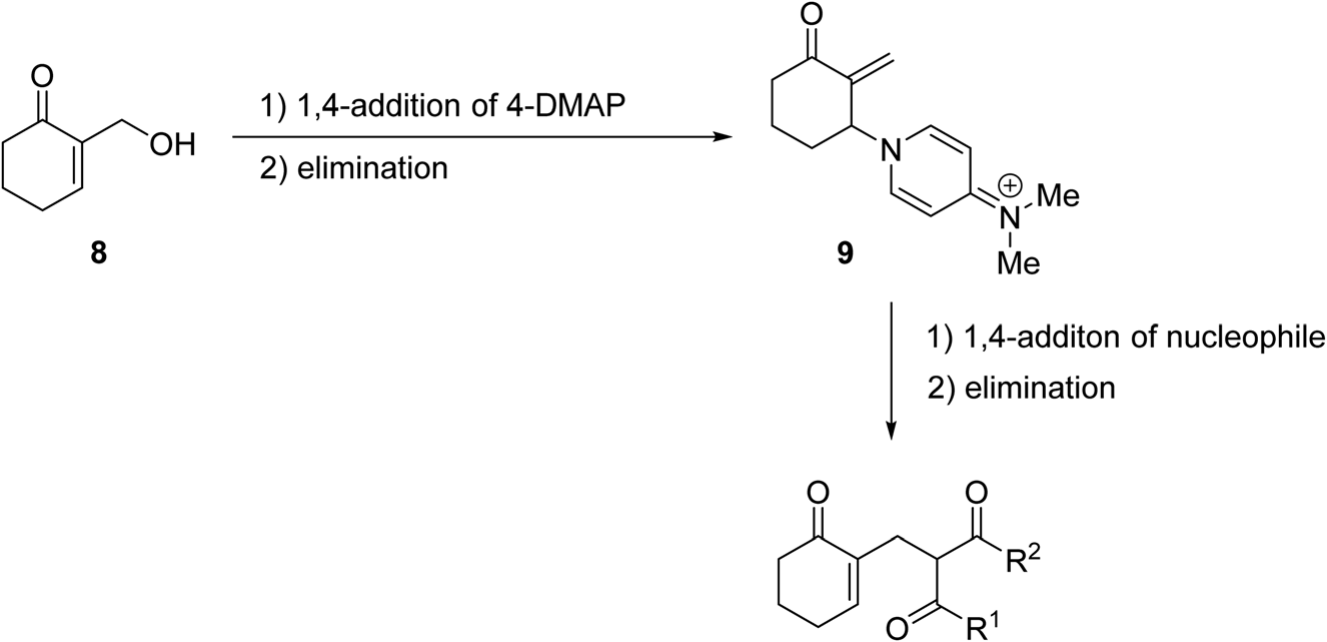
Mechanism of DMAP-catalysed C-allylations.^29^

4-DMAP-catalysed reactions of β-keto esters with cyclic and acyclic Baylis–Hillman alcohols produced the C-allylation adduct exclusively, rather than the expected transesterification or Carroll rearrangement products (Scheme 3).^28,29^ This unexpected outcome is likely due to the presence of an electron-withdrawing group on the activated alkene, which appears to be essential for this transformation. The authors suggest that the mechanism involves conjugate addition of 4-DMAP to the Michael acceptor, followed by the elimination of water to afford an enone intermediate. Subsequent conjugate addition of a β-dicarbonyl enolate and elimination of 4-DMAP provides the final C-allylation product. The overall reaction may be described as a palladium-free Tsuji–Trost-type process.

2,6-Lutidine is a weakly nucleophilic base owing to the steric effect of the two methyl groups on the pyridine ring. Lutidine is a suitable replacement for 4-DMAP in the transesterification of ethyl acetoacetate with Baylis–Hillman alcohols, affording exclusively the desired allylic ester in good yields (Table 10).^28^ No evidence of Carroll decarboxylative rearrangement ketones or C-allylation products was observed.

**Table 10:** 2,6-Lutidine-catalysed transesterification^28^

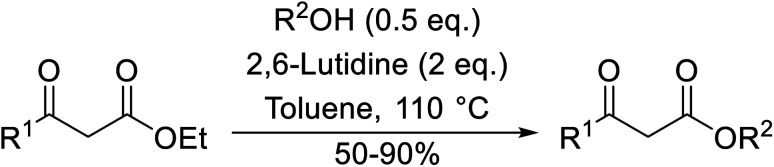				
Entry	R^1^	R^2^	Time (h)	Yield (%)
1	Me	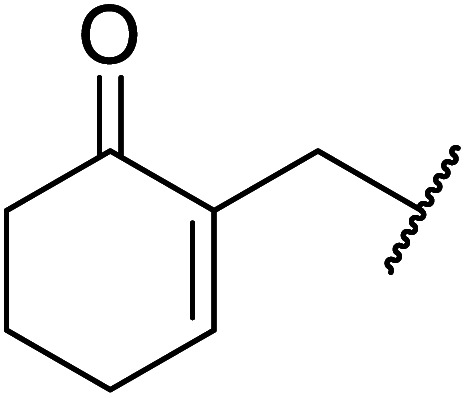	25	90
2	Ph	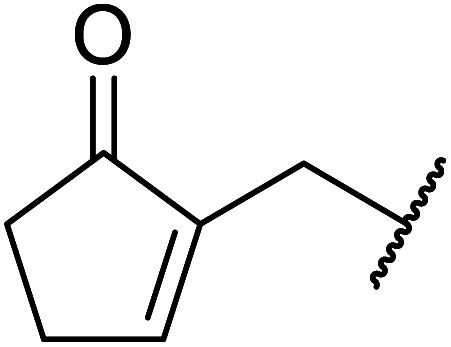	5	50
3	Ph	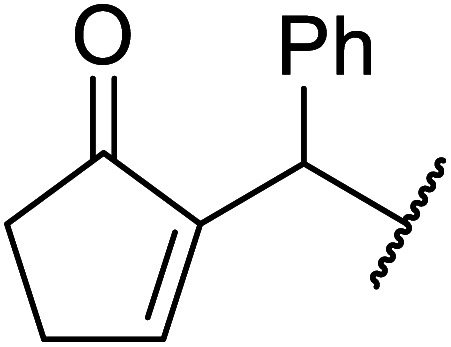	6	63

**Scheme 4:**
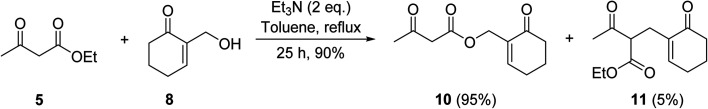
Triethylamine-mediated transesterification of ethyl acetoacetate with Baylis–Hillman alcohol.^28^

The replacement of 2,6-lutidine with triethylamine resulted in a mixture of transesterified and C-alkylated adducts as noted by Mhasni and Rezgui (Scheme 4).^28^ The formation of the minor allylation product was attributed to the lower nucleophilicity of triethylamine which may have instead behaved as a Brønsted base. The use of 4-DMAP in place of triethylamine saw the selective formation of the C-allylation product in a 58% yield.

Triethylamine-catalysed transesterification of a range of β-keto esters with Baylis–Hillman alcohols was subsequently investigated (Table 11). Interestingly, when two possible sites for transesterification were present in the substrate, as with diethyl malonate, only mono-transesterification was observed (entries 4 and 9). When less than two equivalents of triethylamine were employed, the reaction did not reach completion, even after a prolonged reaction time of 72 hours.

**Table 11:** Triethylamine as a catalyst for the transesterification of Baylis–Hillman alcohols^28^

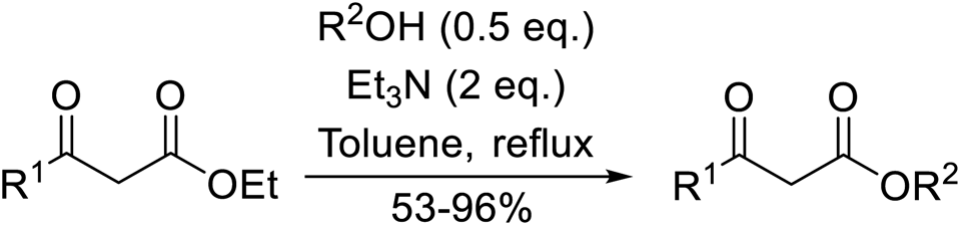				
Entry	R^1^	R^2^	Time (h)	Yield (%)
1	Ph	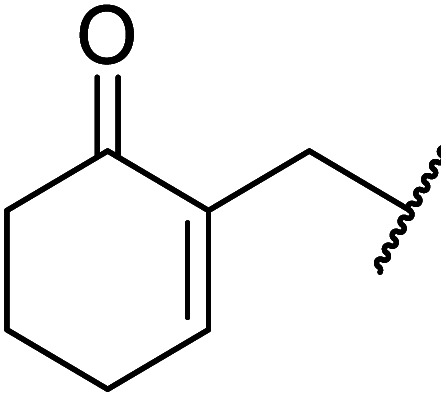	24	85
2	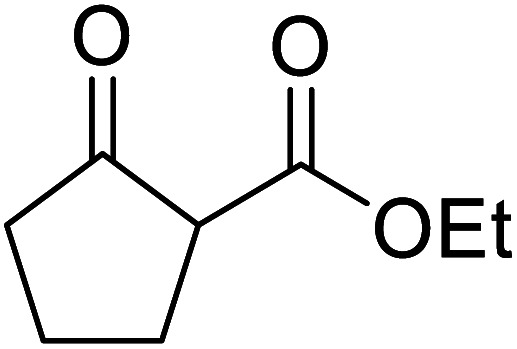	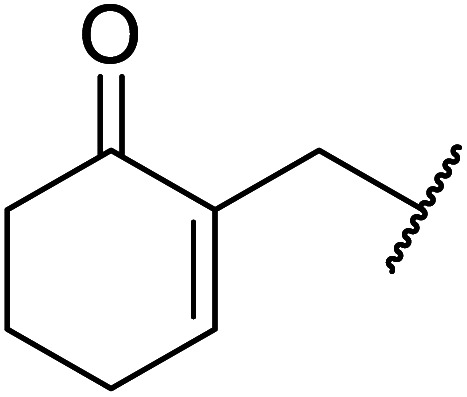	24	54
3	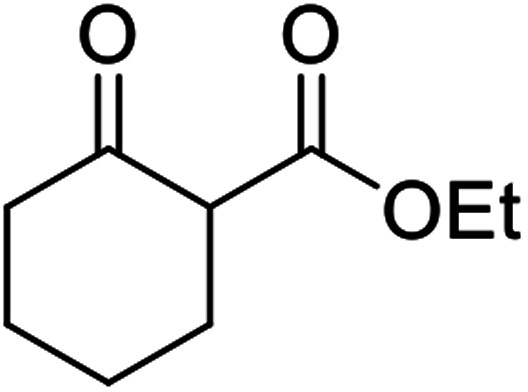	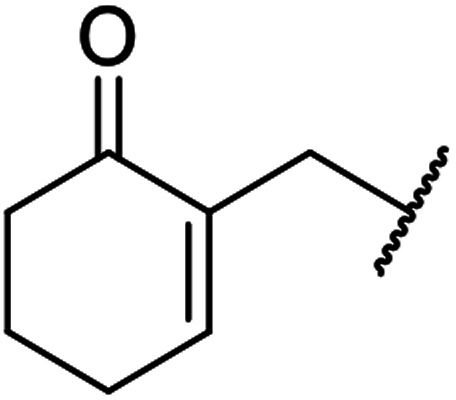	24	90
4	EtO	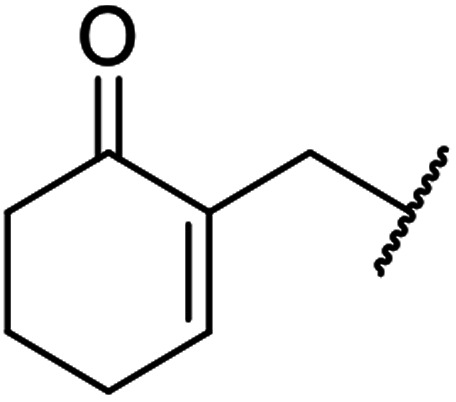	48	72
5	Me	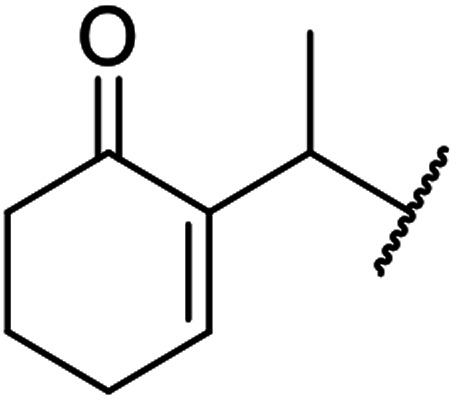	48	76
6	Ph	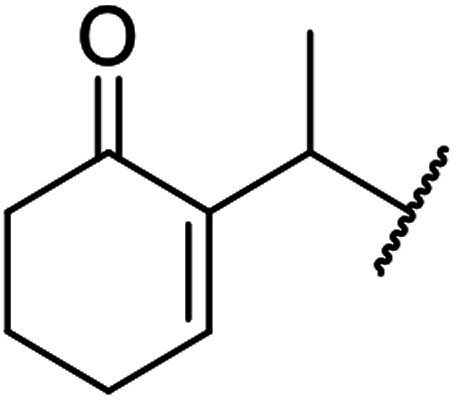	24	96
7	Me	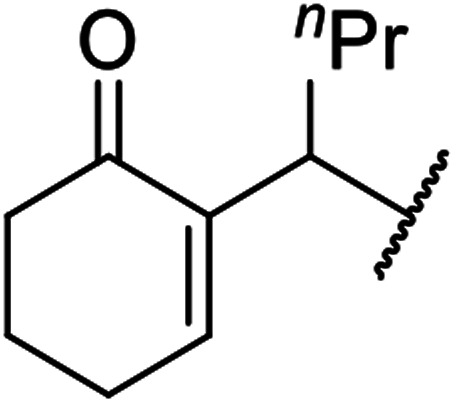	29	89
8	Ph	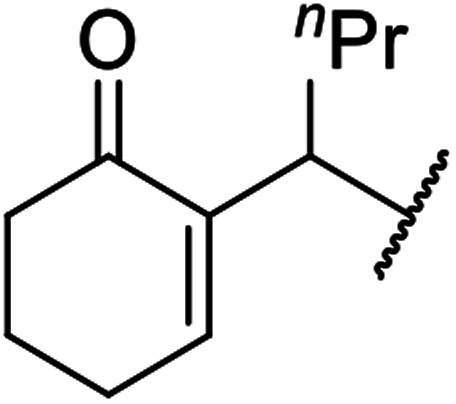	24	90
9	EtO	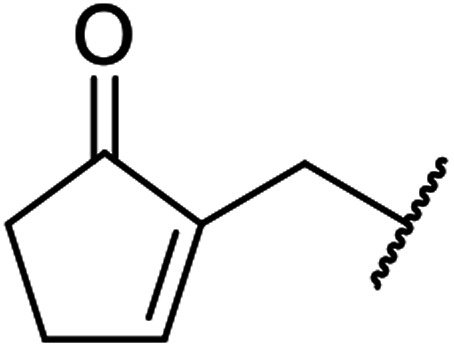	24	53
10	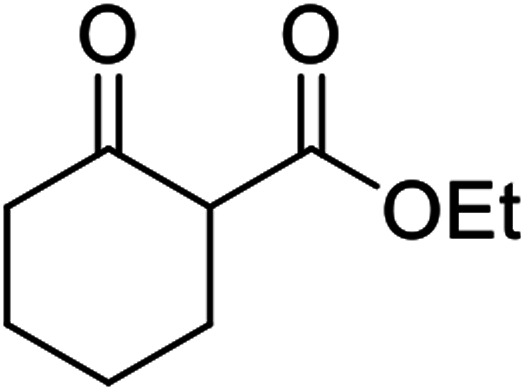	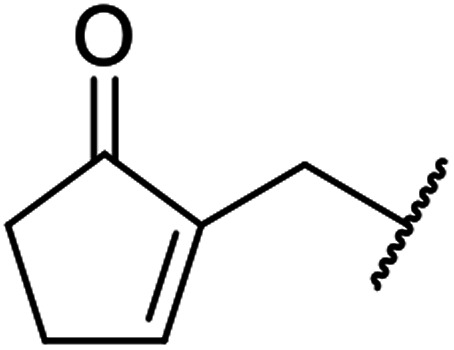	9	56
11	Ph	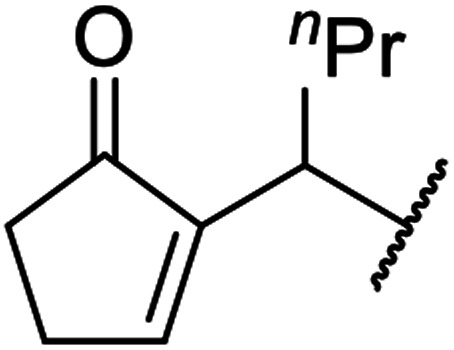	5	89

Further investigations by the same group encompassed a range of allyl (Table 12, entries 1–5), benzyl (entry 6), propargyl (entry 7), and alkyl (entries 8–12) alcohols.^30^ The reactions reached completion within 3–27 hours, affording the target esters in good yields. Carroll decarboxylative rearrangement or C-allylation products were not observed.

**Table 12:** Triethylamine-mediated transesterification of various alcohols^30^

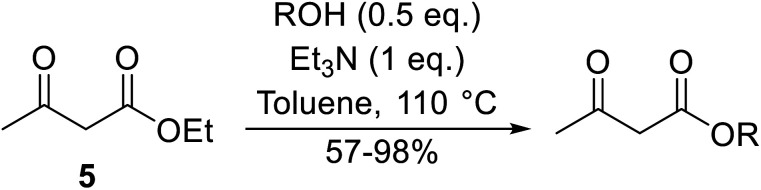			
Entry	R	Time (h)	Yield (%)
1	PhCH=CHCH_2_	5	95
2	CH_3_CH=CHCH_2_	10	91
3	H_2_C=C(CH_3_)CH_2_	5	87
4	H_2_C=CHCH(CH_3_)	6	82
5	H_2_C=CHC(CH_3_)_2_	24	57
6	Bn	3	72
7	HC≡CCH_2_	7	78
8	(CH_3_)_2_CHCH_2_	25	74
9	^*n*^Bu	27	80
10	^*n*^CH_3_(CH_2_)_9_	22	97
11	Cy	8	84
12	Menthyl	21	98

**Fig. 2:**
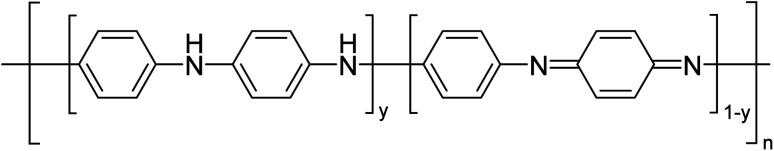
General formula for polyaniline salt.

Palaniappan and Shekhar have reported the replacement of conventional amine catalysts with eco-friendly polyaniline salts including polyaniline-sulfate, polyaniline-nitrate and polyaniline-hydrochloride (Table 13).^31^ Polyaniline is a *para*-linked phenylene amineimine as described by the general formula in Fig. 2. Polyaniline sulfate was more efficient than polyaniline nitrate, which in turn was found to be more efficient than polyaniline hydrochloride. Yields with aliphatic alcohols were excellent, generally reaching over 90% (entries 1–8). Propargyl alcohols reacted smoothly (entry 11) while allylic alcohol proved more problematic with only a 23% yield recorded (entry 15). Some of the advantages of polyaniline salt catalysts include their easy preparation, improved recovery and reusability, and low toxicity. The salts were recovered and reused three times, with no significant loss of yield noted.

**Table 13:** Polyaniline salt catalysis^31^

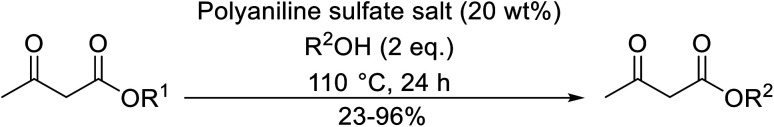			
Entry	R^1^	R^2^	Yield (%)
1	Et	^*n*^Hex	96
2	Me	^*n*^Hex	96
3	Ph	^*n*^Hex	90
4	Et	^*n*^Bu	94
5	Et	^*n*^Oct	94
6	Et	^*n*^CH_3_(CH_2_)_9_	90
7	Et	^*n*^CH_3_(CH_2_)_11_	92
8	Et	^*n*^CH_3_(CH_2_)_21_	94
9	Et	EtO(CH_2_)_2_	96
10	Et	^*n*^BuO(CH_2_)_2_	96
11	Et	HC≡C(CH_2_)_2_	90
12	Et	Menthyl	94
13	Et	Cy	85
14	Et	Bn	72
15	Et	H_2_C=CHCH_2_	23

Hagiwara *et al.* have demonstrated the *N*-methylaminopropylated silica (NMAP) catalysed self-condensation of aldehydes.^32,33^ The same group found that *N*,*N*-diethylaminopropylated silica (NDEAP) efficiently catalysed the transesterification of β-keto esters (Table 14).^34^ Distribution of the *N*,*N*-diethylamino residue over the wide surface area of the silica gel allows for a high turnover number (TON) of 425 and a turnover factor (TOF) of 170. Alcohols containing acid-sensitive or base-sensitive substituents were converted in excellent yields (entries 4–6), highlighting the mild nature of this protocol. Substrates bearing an olefinic moiety, which would normally be sensitive to metal-based catalysis, were converted to the transesterified product in good yields (entry 2). C-2-substituted β-keto esters were similarly compatible (entry 11). 2-(4-Hydroxyphenyl) ethyl alcohol was observed to react exclusively at the primary alcohol group, facilitating a regioselective transformation (entry 16). The catalyst could be reused five times without requiring re-activation.

**Table 14:** NDEAP-SiO_2_ catalysis^34^

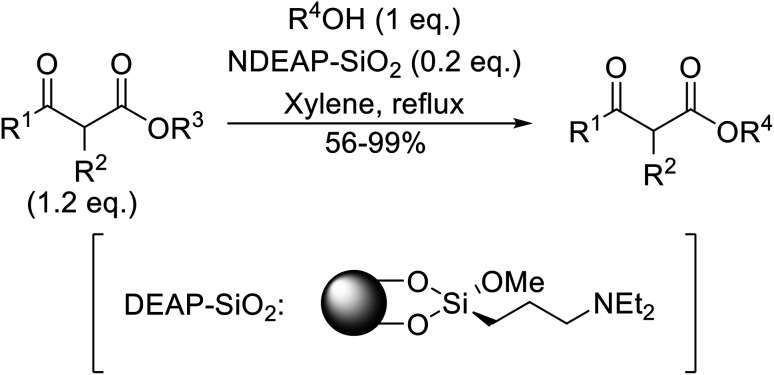						
Entry	R^1^	R^2^	R^3^	R^4^	Time (h)	Yield (%)
1	Me	H	Me	Ph(CH_2_)_2_	24	56
2	Me	H	Me	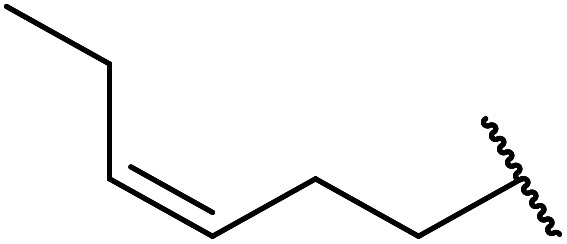	24	70
3	Me	H	Me	PhCH_2_	5.5	75
4	Me	H	Me	AcO(CH_2_)_5_	4	84
5	Me	H	Me	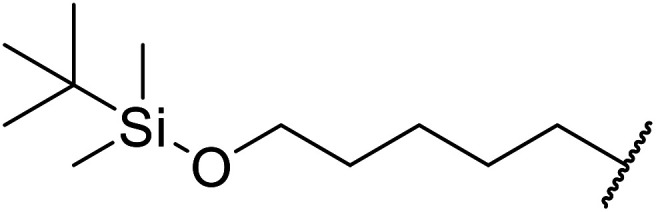	2	88
6	Me	H	Me	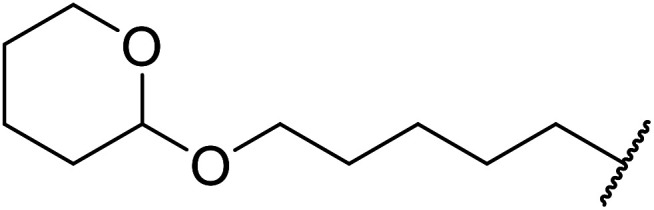	2	85
7	Me	H	Me	Cholester	24	89
8	Me	H	Me	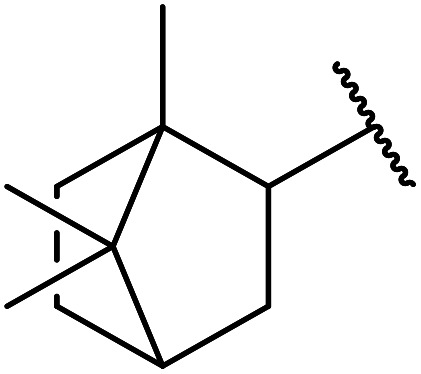	21	72
9	Me	H	Me	Menth	7	99
10	Me	H	^*t*^Bu	Menth	0.5	82
11	Me	Bn	Me	Menth	8.5	76
12	Me	H	Me	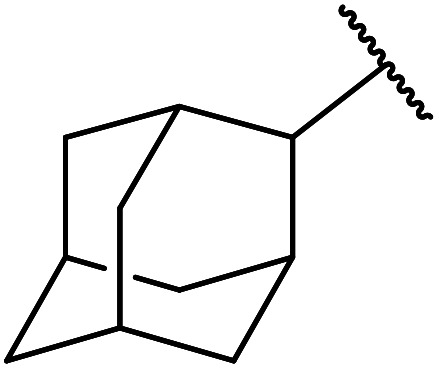	6.5	97
13	Me	H	Me	Terpine	3.5	78
14	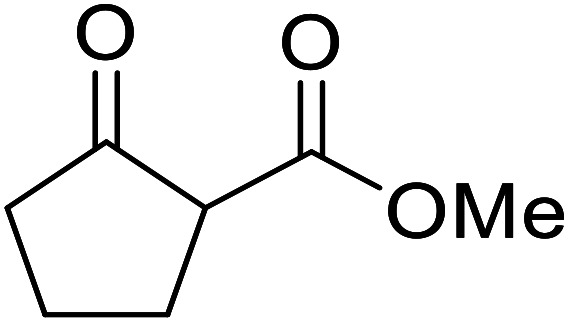			Ph(CH_2_)_2_	7	95
15	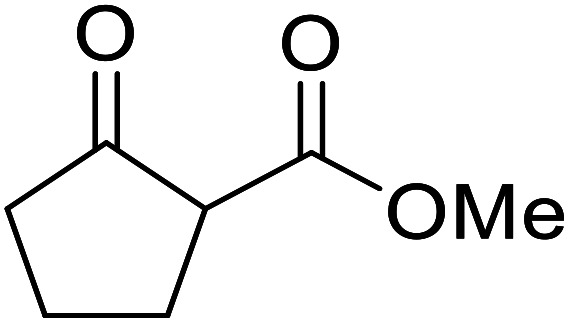			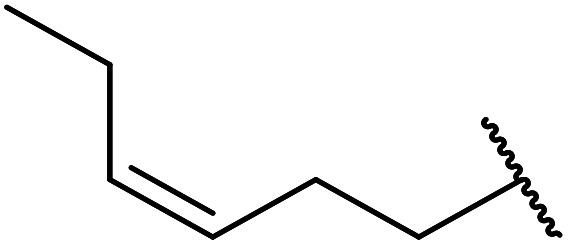	3.5	91
16	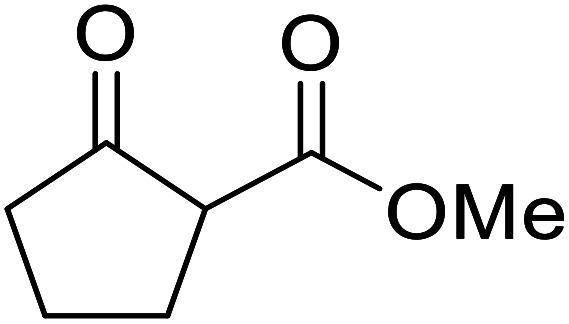			HOC_6_H_4_(CH_2_)_2_	3	99
17	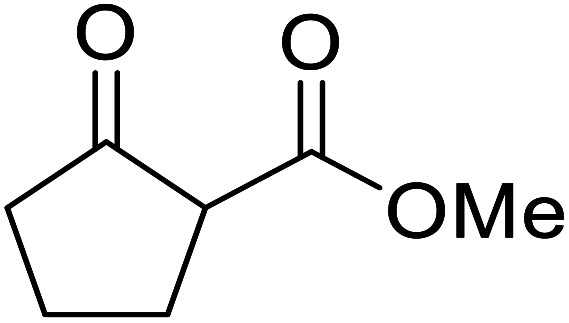			AcO(CH_2_)_6_	4	84
18	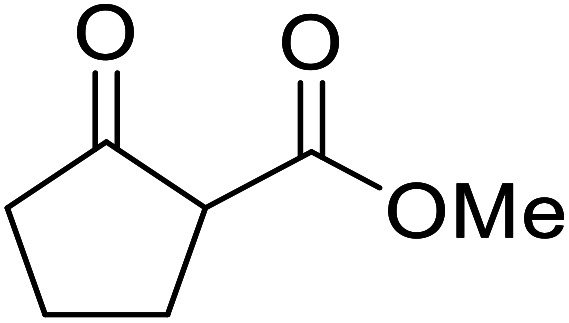			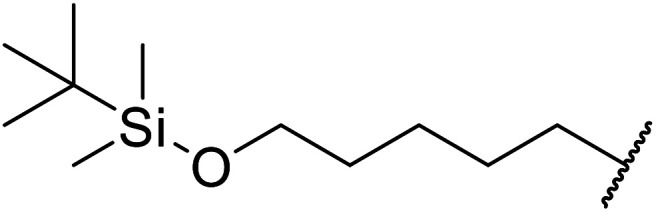	2	88
19	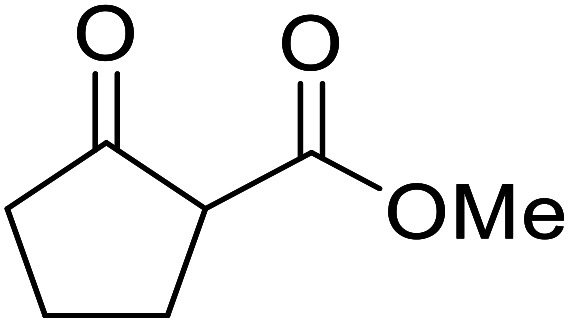			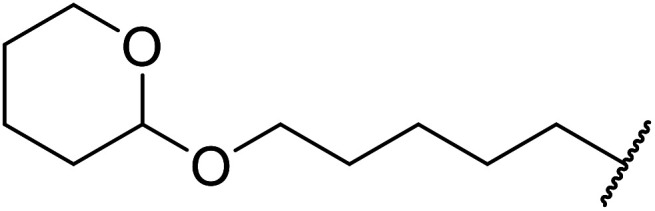	1.5	85
20	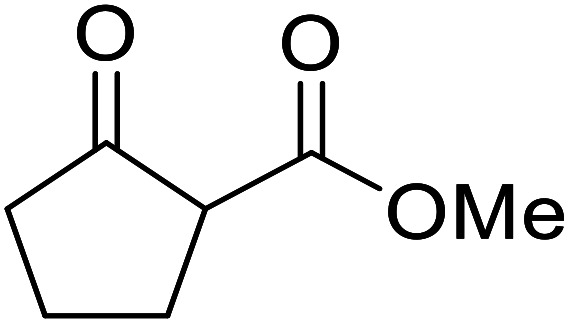			Cholester	4.5	89
21	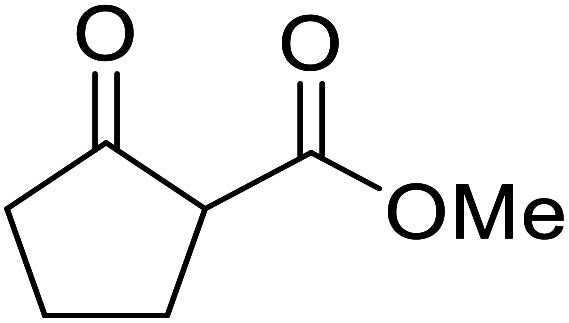			Menth	3.5	94

**Fig. 3:**
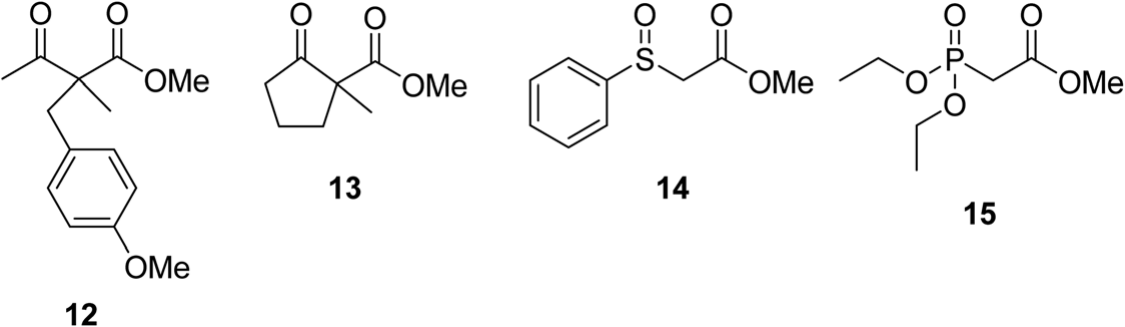
Incompatible transesterification substrates.

When the transesterification of keto esters 12 and 13 with menthol was attempted under these conditions, only starting material was recovered (Fig. 3). The transesterification of methyl α-phenylsulfinylacetate (14) and methyl diethylphosphonoacetate (15) was similarly unsuccessful. The requirement for acidic α-protons would suggest that this transformation proceeds *via* a ketene intermediate.

Hexamethylenetetramine (HMT) has been identified by Ribeiro and co-workers as an efficient transesterification catalyst (Table 15).^35^ HMT is an inexpensive, non-hygroscopic, and stable reagent with low toxicity.^36^ Four different methods were compared utilising different combinations of solvents, substrate loadings or the use of a Dean–Stark trap (Table 15). In general, method C was found to be the most effective. Transesterification of an α-substituted acetoacetate with *tert*-butyl alcohol returned low yields (entry 14), which can be attributed to the bulky nature of the substrate. Introduction of α,α-disubstituted β-keto esters resulted in recovery of starting material only, suggesting reaction *via* a ketene intermediate (entries 15 and 16). Other esters, such as ethyl phenylacetate (entries 18 and 19) or the triglyceride trimyristin (entry 20), proved incompatible.

**Table 15:** Transesterifications catalysed by HMT^35^

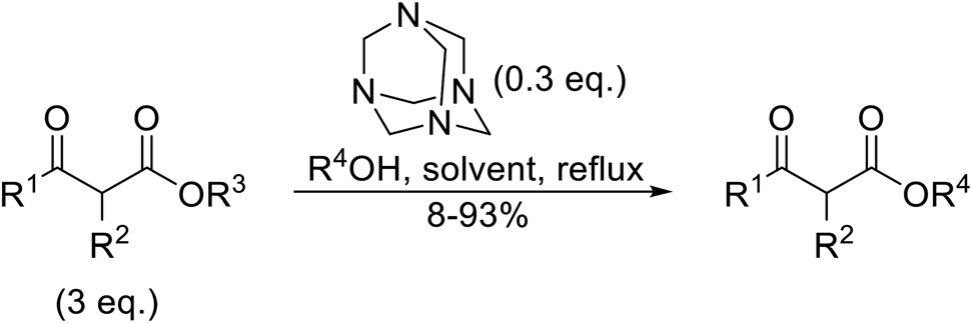						
Entry	R^1^	R^2^	R^3^	R^4^	Method^a^	Yield (%)
1	Me	H	Me	(−)-Menthyl	A	73
2	Me	H	Me	(−)-Menthyl	B	86
3	Me	H	Me	(−)-Menthyl	C	93
4	Me	H	Me	Cy	A	70
5	Me	H	Me	Cy	C	85
6	Me	H	Me	^*n*^CH_3_(CH_2_)_15_	A	78
7	Me	H	Me	^*n*^CH_3_(CH_2_)_15_	C	84
8	Me	H	Me	Bn	C	92
9	Me	H	Me	^*t*^Bu	D	60
10	Ph	H	Et	(−)-Menthyl	C	67^c^
11	Me	H	Et	(−)-Menthyl	B	75
12	Me	H	Et	(−)-Menthyl	C	89
13	Me	H	Et	^*t*^Bu	D	49
14	Me	Me	Et	^*t*^Bu	D	8
15	Me	(Me)_2_	Et	^*t*^Bu	D	n. r.
16	Me	(Me)_2_	Et	(−)-Menthyl	C	n. r.
17	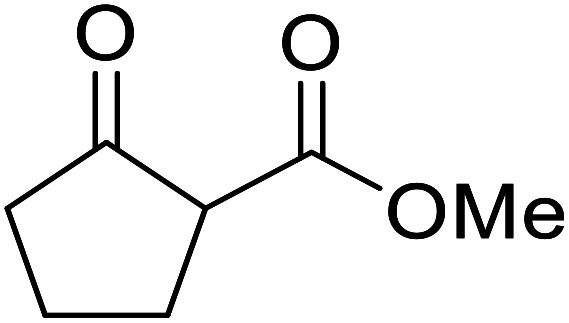			^*n*^Bu	C^b^	63
18	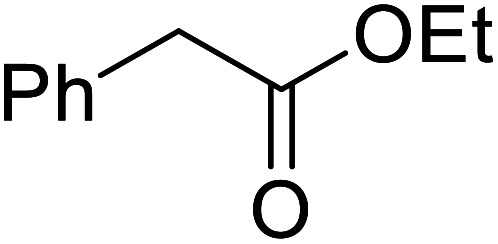			^*n*^Bu	B	n. r.
19	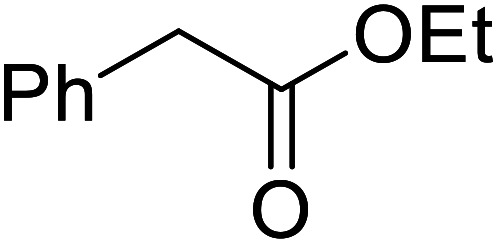			^i^Pr	B	n. r.
20	Trimyristin			Me	B	n. r.

a Reagents and conditions: method A: ester (3 eq.), alcohol (1 eq.), HMT (0.3 eq.), toluene; method B: ester (3 eq.), alcohol (1 eq.), HMT (0.3 eq.), toluene, Dean–Stark trap; method C: ester (3 eq.), alcohol (1 eq.), HMT (0.3 eq.), cyclohexane, Dean–Stark trap; method D: ester (3 eq.), alcohol (excess), HMT (0.3 eq.), toluene, reflux, Dean–Stark trap.

b 1 eq. ester, 1.2 eq. alcohol.

## 4. Lipase catalysts

Conventional transesterification catalysts often require high reaction temperatures. By contrast, lipase-mediated reactions are possible at lower temperatures and do not require organic solvents. Lipases are a class of serine hydrolases which catalyse a range of reactions including ester hydrolysis, esterification and interesterification (ester and acid).^37,38^ Furthermore, these enzymes can typically be recovered and reused.

Córdova and Janda published the first general route for a lipase-catalysed transesterification of β-keto esters (Table 16).^39^*Candida antarctica* lipase B (CALB) was immobilized on a microporous resin allowing the lipase to be recycled without loss of activity. Both primary alcohols (entries 1–6 and 9–12) and secondary alcohols (entries 7 and 8) were compatible substrates, as were allylic alcohols (entries 2, 3, 7, 9 and 11) and propargylic alcohols (entries 4 and 10). CALB also efficiently acetylated the polymer poly(ethylene glycol) monomethyl ether (MeO-PEG5000) (entry 6), which could be used in a polymer-supported synthesis of interesting heterocycles. CALB was found to be selective for aliphatic alcohols over phenols (entry 8). One potential drawback of this methodology is the requirement for a large excess of the β-keto ester.

**Table 16:** Transesterification using CALB^39^

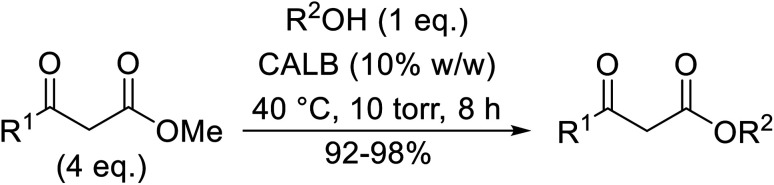			
Entry	R^1^	R^2^	Yield (%)
1	Me	PhCH=CHCH_2_	95
2	Me	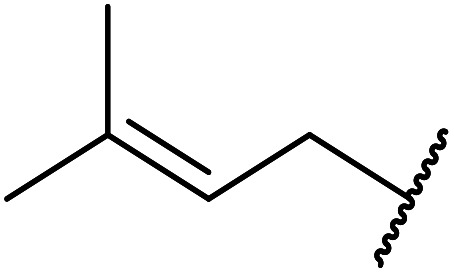	95
3	Me	CH_3_(CH_2_)_2_CH=CHCH_2_	98
4	Me	CH_3_(CH_2_)_2_C≡CCH_2_	92
5	Me	Bn	94
6	Me	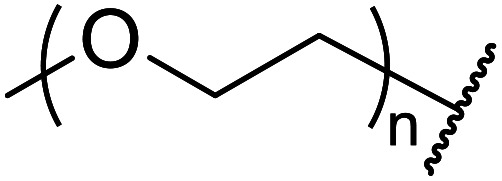	97
7	Me	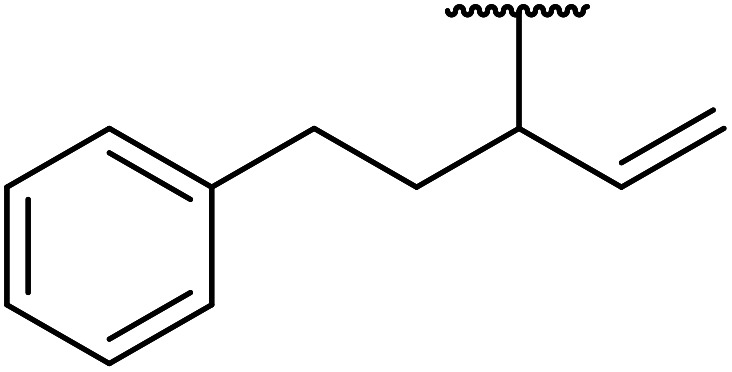	95
8	Me	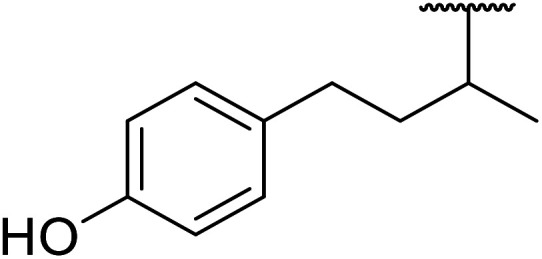	95
9	PhCH_2_CH_2_	PhCH=CHCH_2_	92^a^
10	PhCH_2_CH_2_	CH_3_(CH_2_)_2_C≡CCH_2_	93^a^
11	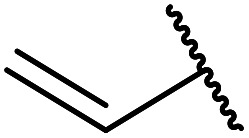	PhCHCHCH_2_	94^a^
12	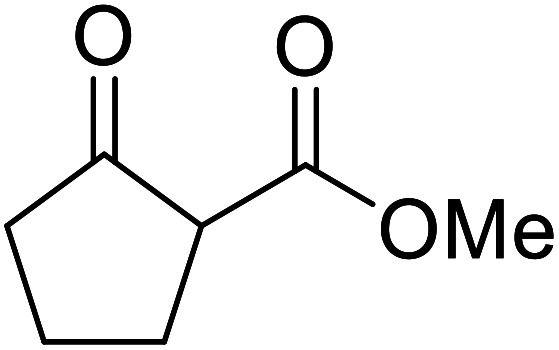	Bn	92^a^

a 1.2 : 1 ratio of alcohol/β-keto.

The same group applied this methodology to the resolution of secondary alcohols *via* their enantioselective transesterification with methyl acetoacetate (Table 17).^39^ Good yields and excellent stereoselectivities were observed with CALB.

**Table 17:** Resolution of secondary alcohols using CALB^39^

Entry	Substrate	Products		Conversion	ee (yield)	ee (yield)
		Alcohol	Ester			
1	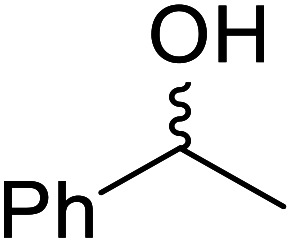	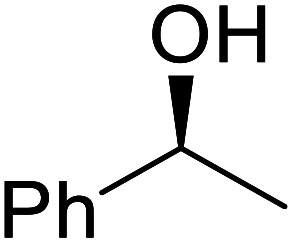	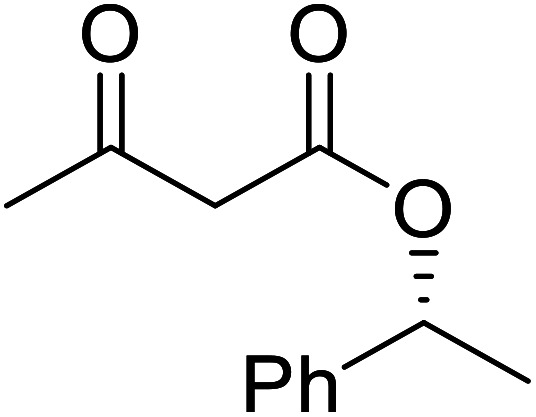	51%	98% (45%)	96% (41%)
2	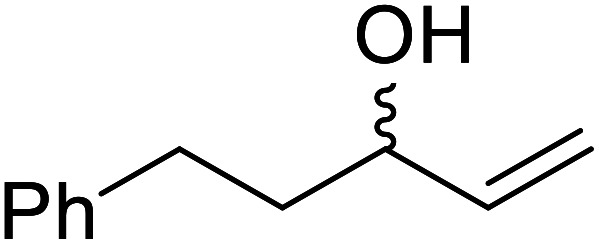	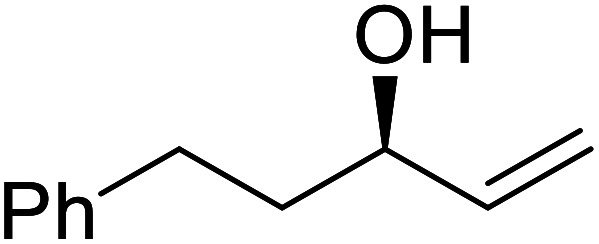	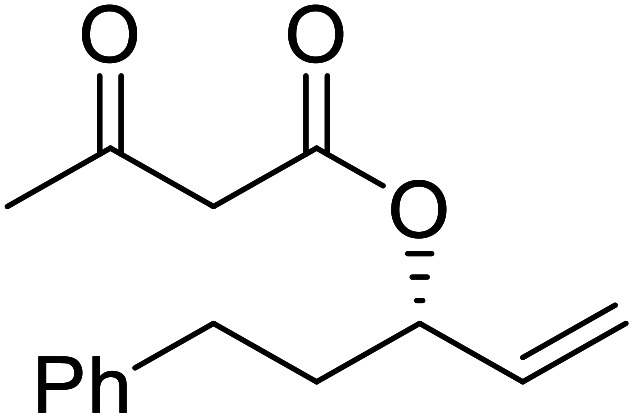	51%	96% (44%)	92% (42%)
3	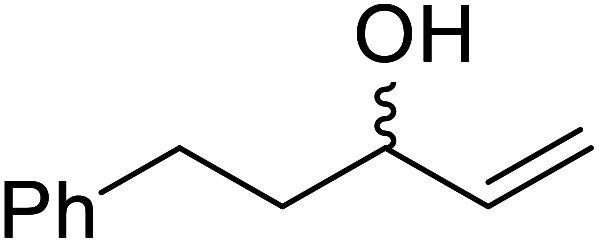	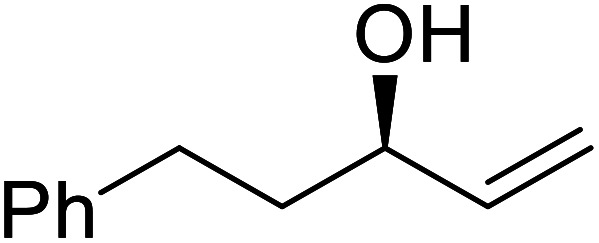	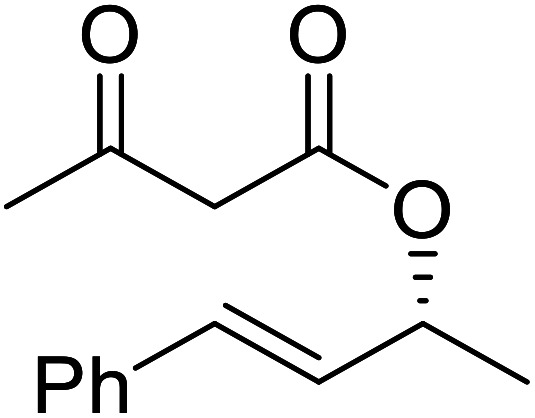	48%	90% (38%)	97% (41%)
4	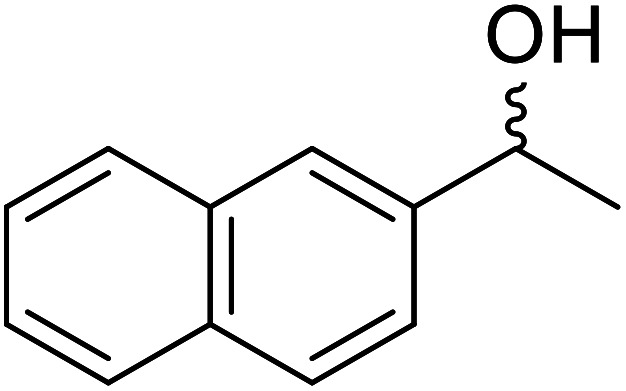	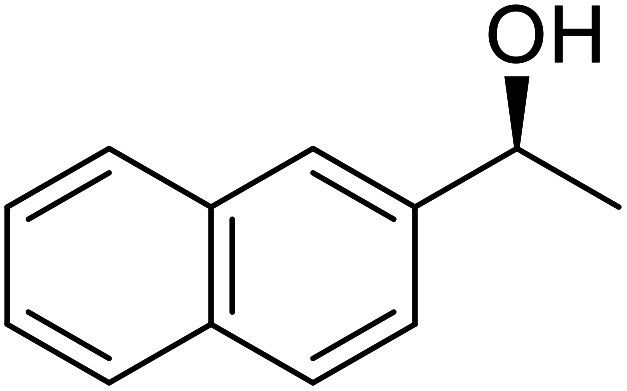	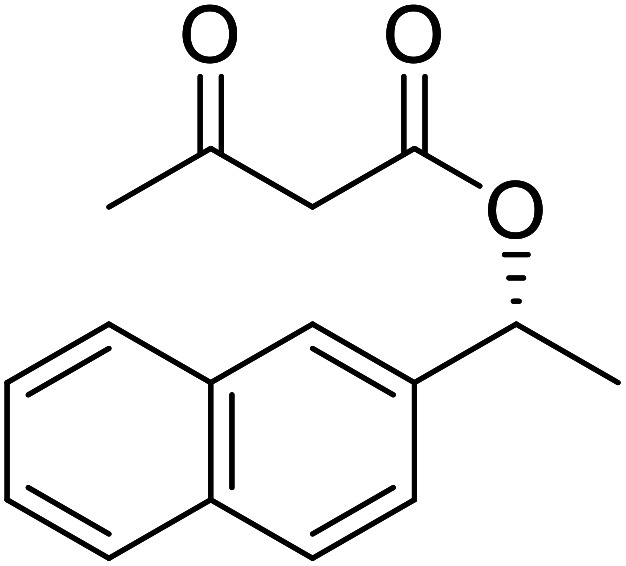	51%	96% (46%)	93% (40%)

Various lipases have been screened by Yadav and Lathi for the transesterification of methyl acetoacetate with *n-*butanol.^40^ Of the three biocatalysts studied, namely Novozym 435 (*Candida antarctica*), Lipozyme RM IM (*Mucor miehei*) and Lipozyme TL IM (*Thermomyces lanuginosus*), Novozym 435 was the most effective at 3% enzyme loading. Yadav and Lathi subsequently developed a novel methodology combining enzyme and microwave catalysis (Table 18).^41^ Microwave-mediated transesterifications were significantly faster than conventional heating. The same three enzymes were compared and Novozyme was again found to be superior. Both primary (entries 1–6) and secondary alcohols (entries 7–9) were examined and in both cases, increased chain length led to decreased conversions. The lipase could be reused with negligible loss of activity, confirming that microwave irradiation does not deactivate the enzyme.

**Table 18:** Novozyme 435 catalysis^41^

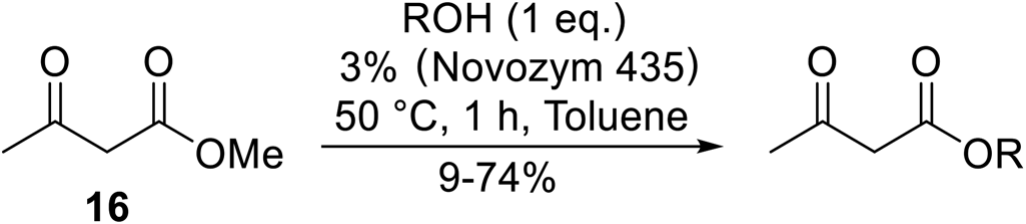			
Entry	R	Conversion (%)	
		Microwave	Conventional
1	^*n*^Pr	74	57
2	^*n*^Bu	72	53
3	^*n*^Pen	66	42
4	^*n*^Hex	61	34
5	^*n*^Oct	55	17
6	^*n*^Dec	33	9
7	^i^Pr	68	48
8	^i^Bu	64	43
9	^*sec*^Pen	57	38

A synergistic combination of enzymes produced good to excellent yields of transesterified β-keto esters in research conducted by Wiśniewska and colleagues.^42^ The enzymes CALB (Novozym 435) from *Candida antarctica*, Novozyme proteases from *Carica papaya*, lipase from *Rhizopus niveus* and lipoprotein lipase from *Pseudomonas* sp*.,* afforded higher yields in combination than individually (Table 19). In the case of 1-phenylethanol, a slightly higher yield was obtained with ethyl acetoacetate (entry 5) than for *tert*-butyl acetoacetate (entry 6). Conversely, for aromatic alcohols containing electron-withdrawing groups, the yields produced were higher for *tert-*butylacetoacetate (entries 8 and 10) than ethyl acetoacetate (entries 7 and 9).

**Table 19:** Transesterification of β-keto esters using a combination of enzymes^42^

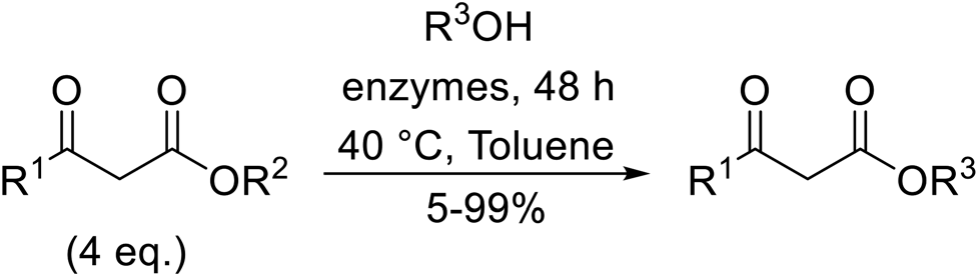				
Entry	R^1^	R^2^	R^3^	Yield (%)
1	Me	Et	Ph(CH_2_)_2_	>99
2	Me	^*t*^Bu	Ph(CH_2_)_2_	>99
3	Me	Et	Bn	98
4	Me	^*t*^Bu	Bn	96
5	Me	Et	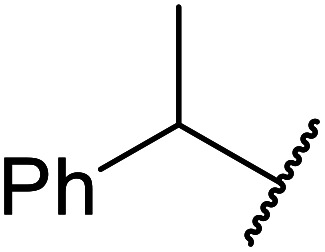	>99
6	Me	^*t*^Bu	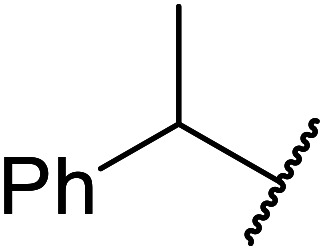	94
7	Me	Et	4-NO_2_–C_6_H_4_(CH_2_)_2_	89
8	Me	^*t*^Bu	4-NO_2_–C_6_H_4_(CH_2_)_2_	>99
9	Me	Et	4-MeO–C_6_H_4_(CH_2_)_2_	83
10	Me	^*t*^Bu	4-MeO–C_6_H_4_(CH_2_)_2_	>99
11	Me	Et	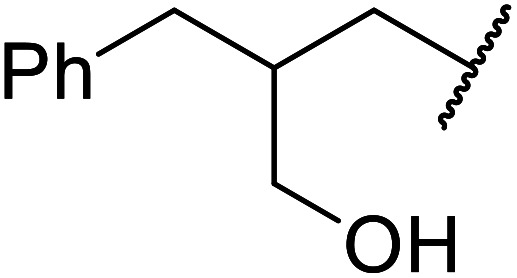	72
12	Me	^*t*^Bu	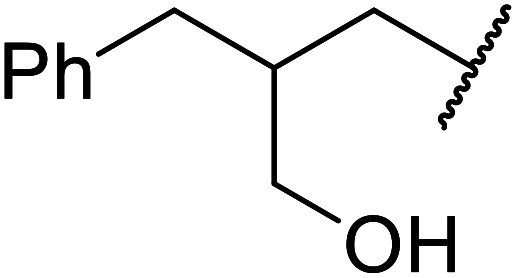	<5
13	Me	Et	PhCH=CHCH_2_	92
14	Me	^*t*^Bu	PhCH=CHCH_2_	<5
15	4-MeO-C_6_H_4_	Et	Ph(CH_2_)_2_	>99
16	4-MeO-C_6_H_4_	Et	Bn	>99

## 5. Non-lanthanide metal catalysts

### 5.1 Transition metals

Reddy *et al.* have described the application of molybdenum–zirconium oxide (Mo–ZrO_2_) as a solid superacid catalyst (Table 20).^43^ The high acidity of Mo–ZrO_2_ is due to its electron deficient state resulting from the introduction of Mo-cations into the ZrO_2_ solid lattice. The wet catalyst was found to be recyclable after a simple filtration with no loss of activity. Neither *tert*-butanol (entry 3) nor phenol (entry 9) proved suitable substrates. However, other aromatic alcohols were compatible (entry 10). Secondary alcohols (entries 2, 5 and 7) and long chain primary alcohols (entries 4 and 6) produced relatively low yields. A notable exception to this trend was cyclohexanol which returned a high 97% yield (entry 11).

**Table 20:** Molybdenum–zirconium oxide-catalysed transesterification of methyl acetoacetate^43^

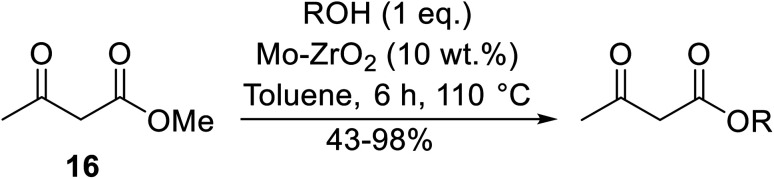		
Entry	R	Yield (%)
1	^*n*^Bu	98
2	^*sec*^Bu	90
3	^*t*^Bu	n. r.
4	^*n*^Pen	75
5	^*sec*^Pen	59
6	^*n*^Oct	62
7	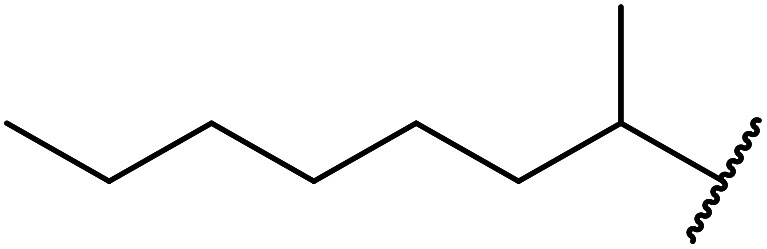	43
8	H_2_C=CHCH_2_	98
9	Ph	n. r.
10	Bn	86
11	Cy	97
12	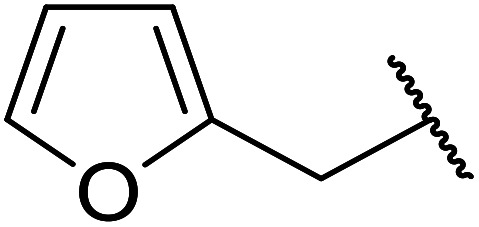	73

Niobium oxide is a proven catalyst for esterification,^44,45^ hydration,^46^ oxidation^47^ and acylation^48^ reactions. Donate and co-workers recently reported the use of niobium oxide for the rapid transesterification of β-keto esters with a range of alcohols (Table 21).^49^ Full conversion was only achieved with *n*-butanol (entry 7). Primary alcohols (entries 1–7, 10) afforded superior conversions and yields compared to secondary (entries 9 and 11) or tertiary alcohols (entries 8). Less than 50% conversion was recorded when benzyl alcohol was introduced (entry 10), possibly due to concomitant dehydration and the formation of benzyl ether. As volatile alcohols may be prone to evaporation, gradual addition of the alcohols significantly increased substrate conversion in these cases. The catalyst may be reused three times without loss of activity.

**Table 21:** Niobium oxide-mediated catalysis^49^

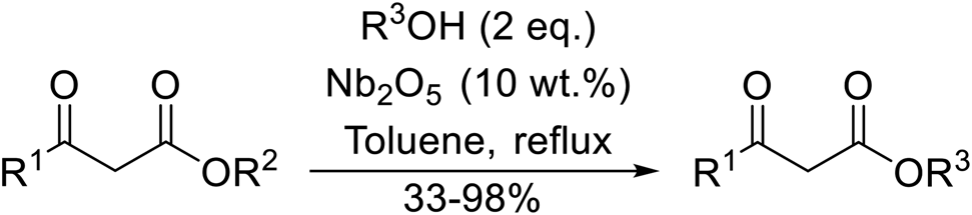						
Entry	R^1^	R^2^	R^3^	Time (h)	Conversion^a^ (%)	Yield (%)
1	Me	Et	H_2_C=CHCH_2_	6	65	52
2	Me	Me	H_2_C=CHCH_2_	6.5	70	58
3	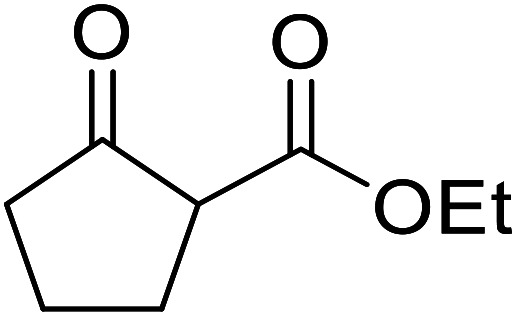		H_2_CCHCH_2_	8	85	57
4	Me	Et	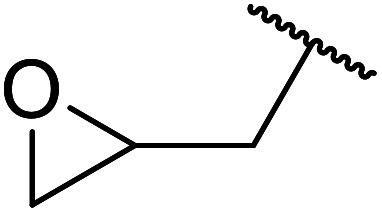	5	75	58
5	Me	Me	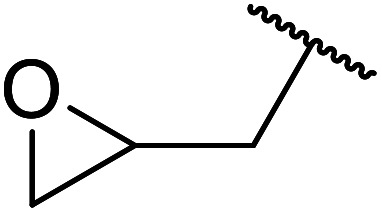	5.5	80	76
6	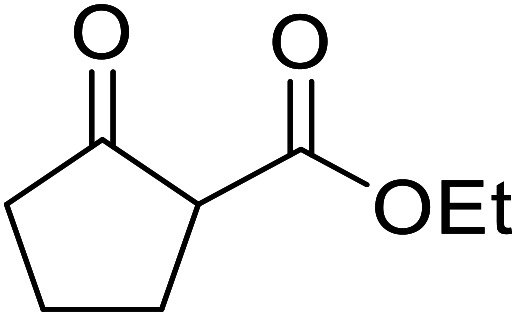		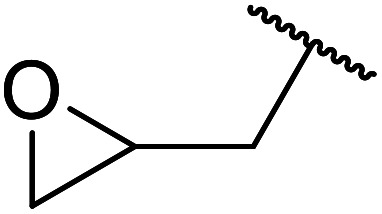	8	70	63
7	Me	Me	^*n*^Bu	5.5–7	100 (100, 100)^b^	98 (93, 87)^b^
8	Me	Me	^*t*^Bu	8	93	60
9	Me	Me	^i^Pr	8	81	63
10	Me	Me	Bn	12	50	33
11	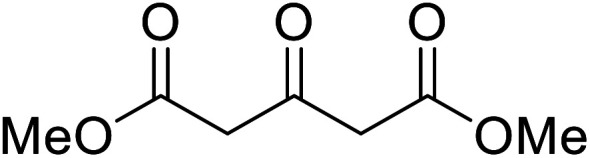		(−)-Menthyl	8	65^c^	61^c^

a Conversion determined by GC of the crude reaction mixture.

b Catalyst was recovered and reused three times without appreciable activity loss.

c Diester product.

[*N*,*N*′-Ethylene bis(salicylideneaminato)]manganese(iii) chloride (Mn(iii) salen complex) has been successfully utilized in Knoevenagel condensations.^50^ Similarly, vanadyl(iv) acetate has been found to be effective for the catalysis of acetylations.^51^ These catalysts are also useful in the transesterification of β-keto esters (Table 22).^52^ The reactions proceeded cleanly with no by-product isolated. Mn(iii) salen consistently outperformed the vanadyl(iv) acetate complex, affording higher yields and slightly shorter reaction times, at a lower catalyst loading of 7 mol% compared to 13.5 mol% of vanadyl(iv) acetate. With this catalyst system, unsaturated alcohols such as propargyl (entry 1), crotyl (entry 4) and cinnamyl alcohol (entry 7) were readily transformed affording esters in high yields. By contrast, tertiary alcohols (entry 10) failed to react in the presence of vanadyl(iv) acetate. Aryl alcohols (entries 7, 8 and 9), cyclic alcohols (entries 2 and 6) and long chain primary alcohols (entries 3 and 11) were converted to the corresponding esters in high yields. When the ambident substrate 2-amino-1-butanol was tested (entry 15), successful transesterification *via* the hydroxyl was observed while the amine group remained intact. Both catalysts were recyclable up to five times without any significant loss in activity.

**Table 22:** Mn(iii) salen and VO(OAc)_2_ catalysis^52^

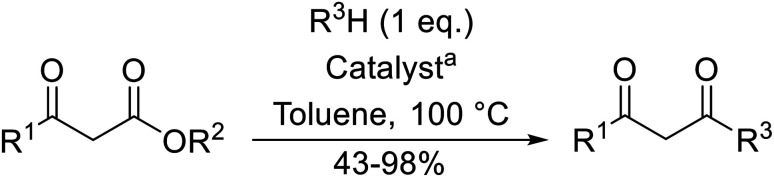						
Entry	R^1^	R^2^	R^3^	Catalyst^a^	Time (h)	Yield^b^ (%)
1	Me	Me	HC≡CCH_2_O	Mn(iii) salen	6	90
				VO(OAc)_2_	8	70
2	Me	Me	Menthol	Mn(iii) salen	8	98
				VO(OAc)_2_	12	95
3	Me	Me	CH_3_(CH_2_)_11_O	Mn(iii) salen	4	98
				VO(OAc)_2_	6	98
4	Me	Me	CH_3_CH=CHCH_2_O	Mn(iii) salen	6	95
				VO(OAc)_2_	8	65
5	Me	Me	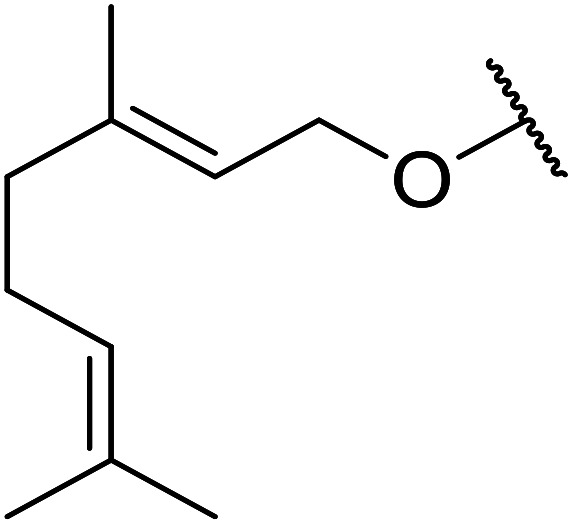	Mn(iii) salen	6	94
				VO(OAc)_2_	8	43
6	Me	Me	CyO	Mn(iii) salen	3	94
				VO(OAc)_2_	6	87
7	Me	Me	PhCH=CHCH_2_O	Mn(iii) salen	8	90
				VO(OAc)_2_	7	50
8	Me	Me	BnO	Mn(iii) salen	8	93
				VO(OAc)_2_	8	75
9	Me	Me	Ph(CH_2_)_2_O	Mn(iii) salen	8	98
				VO(OAc)_2_	8	45
10	Me	Me	^*t*^BuO	Mn(iii) salen	6	56
				VO(OAc)_2_	12	0
11	Me	Me	^*n*^OctO	Mn(iii) salen	2	97
				VO(OAc)_2_	6	80
12	Me	Me	^*n*^HexO	Mn(iii) salen	2	98 (95^c^)
				VO(OAc)_2_	5	91 (90^c^)
13	Me	Me	^*sec*^PenO	Mn(iii) salen	4	88
				VO(OAc)_2_	6	81
14	Me	Me	CH_3_O(CH_2_)_2_O	Mn(iii) salen	3	95
				VO(OAc)_2_	4	87
15	Me	Me	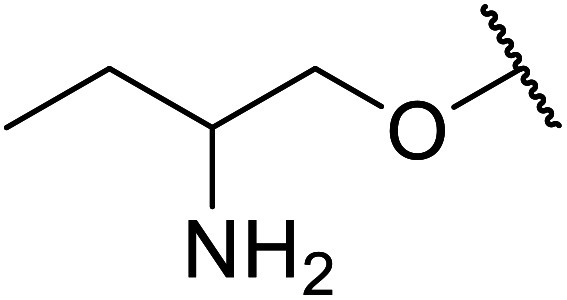	Mn(iii) salen	8	90
				VO(OAc)_2_	8	77
16	Me	Me	BnNH	Mn(iii) salen	4	98
				VO(OAc)_2_	4	98
17	Me	Et	^*n*^HexO	Mn(iii) salen	6	98
				VO(OAc)_2_	8	98
18	Ph	Et	^*n*^HexO	Mn(iii) salen	12	95
				VO(OAc)_2_	12	80

a Mn(iii) salen loading: 7 mol%, VO(OAc)_2_ loading: 13.5 mol%.

b Yields determined by ^1^H-NMR, based on β-keto ester.

c Yield after 5th cycle.

Li *et al.* investigated the utility of manganese chloride (Table 23).^53^ MnCl_2_·4H_2_O catalysed the reaction of *N*-(2-hydroxy)ethylindole and sterically challenging *tert*-butyl acetoacetate in a 90% yield (entry 6). Anhydrous MnCl_2_ also performed well, but a slightly poorer yield of 80% was recorded. Interestingly, addition of 0.4 equivalents of water afforded yields comparable to that of MnCl_2_·4H_2_O. During the reaction, both anhydrous MnCl_2_ and MnCl_2_·4H_2_O were fully soluble in toluene, indicating that solubility does not account for the difference in activity. The authors suggest that a small amount of water might play a role in tuning the acidity of the manganese catalyst.

**Table 23:** Transesterification using manganese chloride^53^

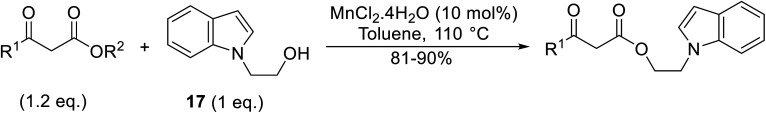				
Entry	R^1^	R^2^	Time (h)	Yield (%)
1	^i^Pr	Me	11	82
2	^*t*^Bu	Et	11	86
3	^*n*^Pr	Et	14	87
4	Ph	Et	11	81
5	4-MeO–C_6_H_4_	Et	11	88
6	Me	^*t*^Bu	11	90

**Scheme 5:**
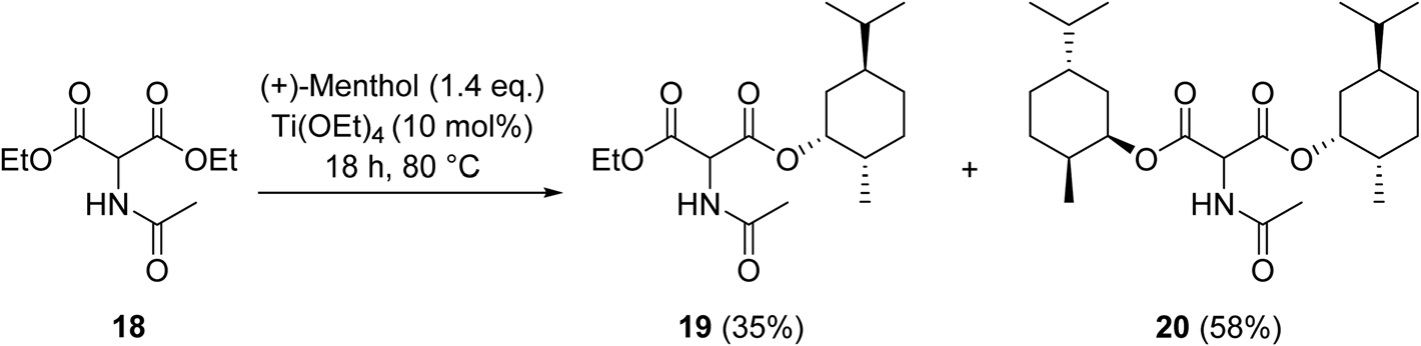
Titanium-catalysed transesterification of diethyl acetamidomalonate (18).

Krasik has demonstrated how titanium ethoxide may be employed to catalyse the transesterification of the sterically hindered β-keto ester diethyl acetamidomalonate (18) generating both the monoester and diester products (Scheme 5).^54^ Titanium-mediated transesterifications are not solely limited to β-keto esters but also encompass other ester substrates.^55–57^

Silver nitrate catalyses the transesterification of β-keto esters under both conventional and non-conventional conditions.^58^ The use of sonication and microwave irradiation afforded moderate to high yields and reduced reaction times (Table 24). Solvent-free, microwave reactions were faster than sonicated reactions in toluene, which were in turn faster than conventionally heated reactions. Yields remained consistent regardless of the conditions employed. Transesterification was successful with primary (entries 1–7, 9 and 10), secondary (entries 8 and 13) and aromatic alcohols (entry 14). Aromatic alcohols with electron-donating substituents (entry 2–5) reacted faster and in higher yields than electron poor phenols (entry 6).

**Table 24:** Transesterification of ethyl acetoacetate using silver nitrate^58^

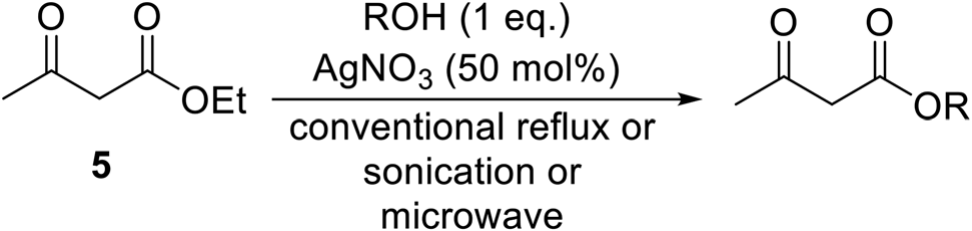							
Entry	R	Conventional		Sonication		Microwave	
		Time (h)	Yield (%)	Time (min)	Yield (%)	Time (min)	Yield (%)
1	Bn	8	89	30	90	3	91
2	4-Me–C_6_H_4_CH_2_	9	82	35	83	3.5	83
3	4-MeO–C_6_H_4_CH_2_	11	83	39	85	4	86
4	3,4-(MeO)_2_–C_6_H_4_CH_2_	11.5	83	40	84	4	86
5	4-Cl–C_6_H_4_CH_2_	9	80	40	83	5	84
6	2-NO_2_–C_6_H_4_CH_2_	13	71	45	72	6	74
7	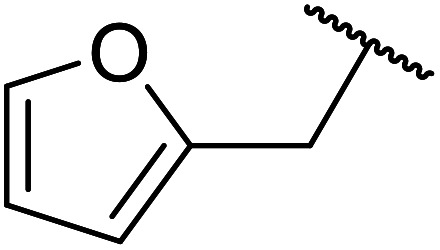	12	80	36	83	5.5	84
8	^i^Pr	12	75	43	77	5	79
9	^*n*^Bu	11	76	44	77	5	78
10	^i^Bu	10	76	45	77	5	79
11	^*t*^Bu	10.5	78	45	79	5.5	81
12	CH_2_=CHCH_2_	11.5	79	43	81	6	81
13	Menthyl	10.5	81	38	83	4.5	84
14	Ph	8.5	81	40	82	5	85

The same research group also investigated ferrous ammonium sulfate (FAS), also known as Mohr's salt, and ammonium nickel sulfate (ANS) as potential transesterification catalysts (Table 25).^59^ Better results were obtained with FAS rather than ANS. While both catalysts exist in different oxidation states, the nickel catalyst slowly oxidizes over time. The reaction times were similar to those observed with silver nitrate in the order of microwave < sonication < conventional.

**Table 25:** Comparison of ferrous ammonium sulfate and ammonium nickel sulfate catalysis^59^

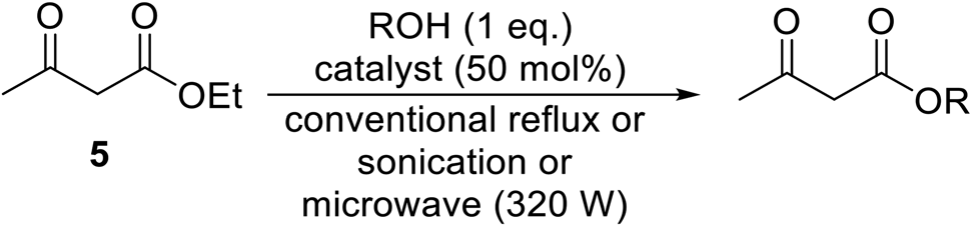								
Entry	R	Catalyst	Conventional		Sonication		Microwave	
			Time (h)	Yield (%)	Time (min)	Yield (%)	Time (min)	Yield (%)
1	Bn	FAS	10	86	45	87	5	90
		ANS	12	85	46	86	5	88
2	4-Me–C_6_H_4_CH_2_	FAS	12	84	48	85	6	88
		ANS	14	82	50	83	7	86
3	4-MeO–C_6_H_4_CH_2_	FAS	14	81	50	84	8	86
		ANS	15	80	54	81	8	83
4	3,4-(MeO)_2_–C_6_H_4_CH_2_	FAS	14	80	52	81	9	83
		ANS	16	77	56	79	9	80
5	4-Cl–C_6_H_4_CH_2_	FAS	13	80	50	80	7	81
		ANS	14	78	53	80	7	81
6	2-NO_2_–C_6_H_4_CH_2_	FAS	14	77	53	79	9	80
		ANS	15	77	58	79	9	80
7	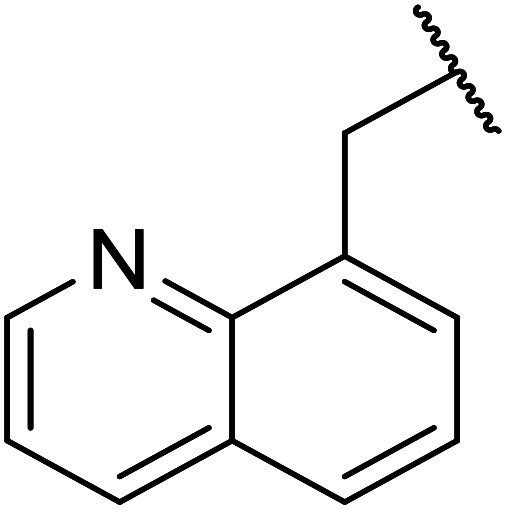	FAS	13	78	55	78	8	79
		ANS	14	79	55	78	8	79
8	Ph(CH_2_)_2_	FAS	10	85	46	86	6	88
		ANS	12	85	46	86	6	88
9	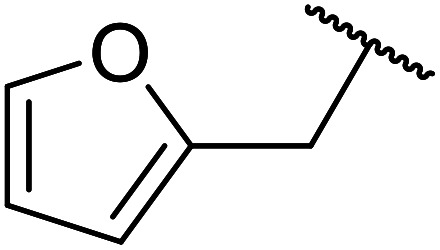	FAS	13	80	53	81	8	83
		ANS	25	78	55	82	8	83
10	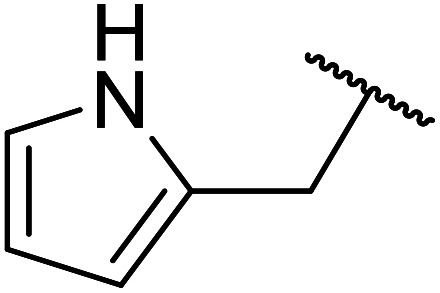	FAS	14	78	56	79	9	81
		ANS	16	76	60	79	10	81
11	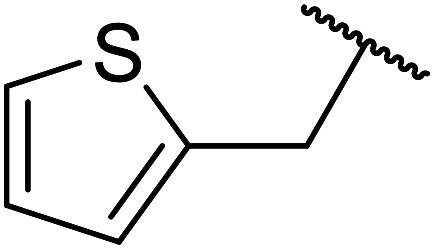	FAS	14	76	58	78	10	80
		ANS	16	74	60	78	10	79
12	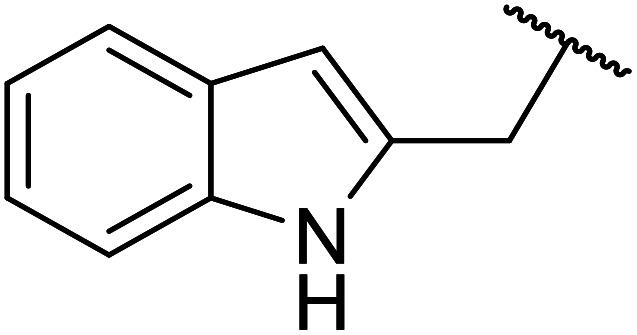	FAS	12	77	55	80	9	83
		ANS	13	75	56	81	9	82

Ag–Cu catalysts supported on hydrotalcite-like material (Ag–Cu/HTs) have recently been developed (Table 26).^60^ Ag–Cu has vacant orbitals which likely interact with the two carbonyl groups of the β-keto esters to form a cyclic intermediate during the reaction.^61^ The Ag : Cu molar ratio in the catalyst had a major influence on yields. When monometallic Cu/HTs was employed for cinnamyl acetoacetate, the final yield was 72%. By contrast, a maximum yield of 94% was recorded using an Ag : Cu catalyst with a molar ratio of 1.2 : 1. Likewise, when monometallic Ag/HTs was introduced, a lower 86% yield was achieved. These results suggest that bimetallic Ag–Cu nanoparticles are more active than monometallic Ag or Cu nanoparticles, most likely due to an interaction between Cu and Ag. Electron transfer from Cu to Ag within these bimetallic nanoparticles provides better resistance to oxidation than monometallic Cu nanoparticles. The yields from allylic alcohols (entries 2 and 6) were significantly higher than from aliphatic alcohols (entries 3 and 7). Simple esters afforded lower yields in comparison to β-keto esters. The transesterification of ethyl acetoacetate with benzyl alcohol, for example, afforded a 97% yield after five hours (entry 1). By contrast, reaction of ethyl acetate and benzyl alcohol returned a reduced yield of 80% (entry 12).

**Table 26:** Catalysis of ethyl acetoacetate using silver-copper nanoparticles^60^

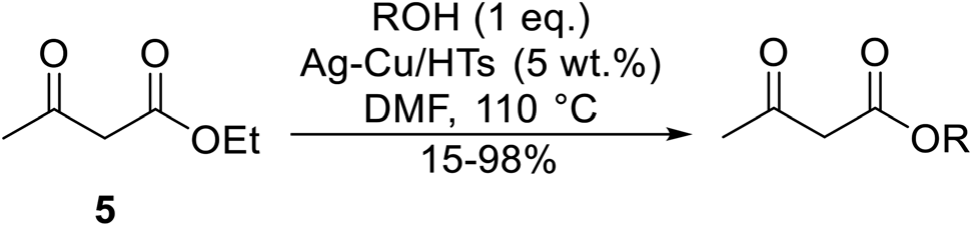			
Entry	R	Time (h)	Yield^a^ (%)
1	Bn	5	97
2	PhCH=CHCH_2_	6	98^b^
3	Ph(CH_2_)_3_	24	89
4	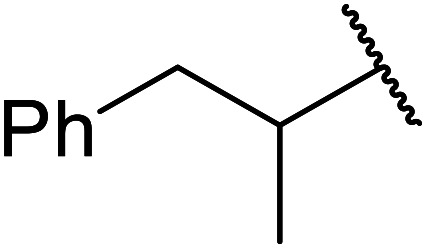	24	52
5	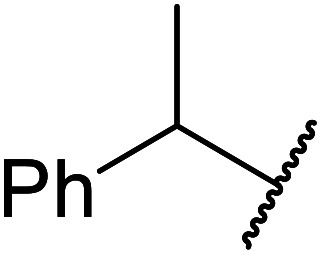	24	15
6	CH_3_CH=CHCH_2_	24	88
7	^*n*^Bu	24	66
8	CH_2_=CHCH_2_	24	76
9	^*n*^Pr	24	67
10	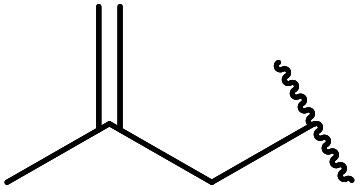	24	68
11	^*n*^Hex	24	64
12	Bn	24	80^c^

a Determined by ^1^H-NMR analysis of the crude reaction mixture.

b Yield of isolated products after column chromatography.

c Ethyl acetate starting material.

Silver triflate has met with some success in the catalysis of transesterifications.^62^ When aliphatic and aromatic alcohols were subjected to esterification with ethyl acetoacetate, the expected products were obtained in high yields (Table 27, entries 1–13). Reactions with aromatic alcohols containing electron-donating substituents (entries 2–4) afforded the corresponding acetoacetates in 6–8 hours in 90% yield, whereas alcohols bearing electron-withdrawing substituents reacted more slowly (entry 5). Allylic alcohols also furnished β-keto esters in good yields, with no evidence of Carroll rearrangement (entries 7 and 8). As expected, increasing the steric bulk of the alcohol resulted in increased reaction times and reduced yields. Primary alcohols (entries 1–10) proved more amenable than secondary alcohols (entries 12 and 13), which were in turn more reactive than tertiary alcohols (entry 11). Surprisingly, *tert-*butyl acetoacetate (entry 17) afforded a higher yield than secondary or primary β-keto esters (entries 14–16) in contrast to previous studies.

**Table 27:** Silver triflate-mediated catalysis^62^

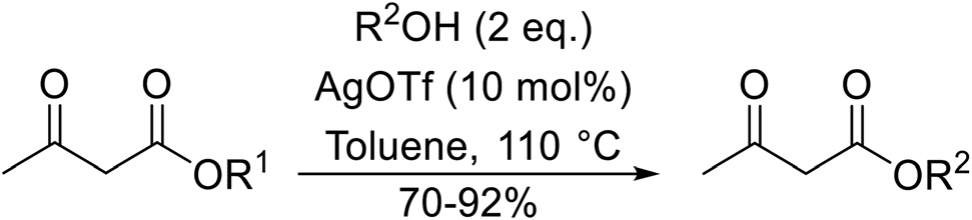				
Entry	R^1^	R^2^	Time (h)	Yield (%)
1	Et	Bn	7	90
2	Et	4-MeO–C_6_H_4_CH_2_	6.5	92
3	Et	3,4-(MeO)_2_–C_6_H_3_CH_2_	6	90
4	Et	4-Cl–C_6_H_4_CH_2_	8	84
5	Et	1-NO_2_–C_6_H_4_CH_2_	19	70
6	Et	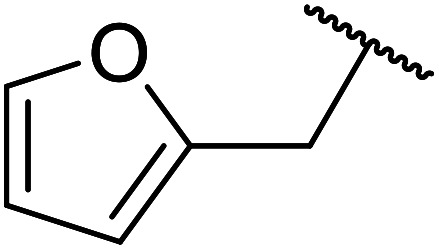	10	80
7	Et	CH_3_CH=CHCH_2_	9	85
8	Et	PhCH=CHCH_2_	8	87
9	Et	^*n*^Bu	6	81
10	Et	^i^PrCH_2_	9	79
11	Et	^*t*^Bu	15	78
12	Et	^i^Pr	10	77
13	Et	Cy	12	78
14	Me	Bn	10	79
15	^i^Bu	Bn	7	83
16	CH_2_CH=CH_2_	Bn	8	82
17	^*t*^Bu	Bn	6	85

Both iron sulfate and copper sulfate constitute efficient, inexpensive and neutral transesterification catalysts.^63^ Methyl and ethyl β-keto esters were successfully transesterified with one equivalent of alcohol in the presence of anhydrous iron sulfate or copper sulfate at 80 °C (Table 28). Primary (entries 1, 2, 5–7 and 9), secondary (entries 3, 4, 10–12), tertiary (entry 8), aromatic (entries 5–7) and unsaturated alcohols (entries 6, 11 and 12) were all compatible with these conditions. These catalysts are selective towards β-keto esters, with simple esters remaining unreacted. Sterically crowded *tert*-butanol was converted to the corresponding *tert-*butyl ester in a moderate yield (entry 8). In general, the yields obtained using either iron sulfate or copper sulfate were comparable.

**Table 28:** Catalysis with iron sulfate or copper sulfate^63^

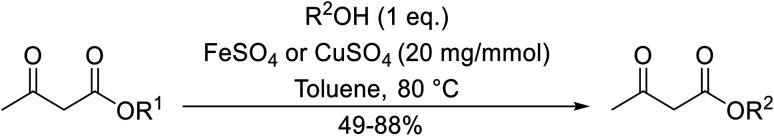					
Entry	R^1^	R^2^	Catalyst	Time (h)	Yield (%)
1	Et	^*n*^Bu	FeSO_4_	2	85
			CuSO_4_	2	88
2	Et	^*n*^Hex	FeSO_4_	2	80
			CuSO_4_	2	78
3	Et	Cy	FeSO_4_	2.5	87
			CuSO_4_	3	83
4	Et	Menthyl	FeSO_4_	2.5	76
			CuSO_4_	3	75
5	Et	4-MeO–C_6_H_4_CH_2_	FeSO_4_	2	79
			CuSO_4_	2	82
6	Me	PhCH=CHCH_2_	FeSO_4_	2.5	78
			CuSO_4_	3	75
7	Et	4-NO_2_–C_6_H_4_CH_2_	FeSO_4_	2.5	75
			CuSO_4_	2.3	73
8	Et	^*t*^Bu	FeSO_4_	8	53
			CuSO_4_	8.5	49
9	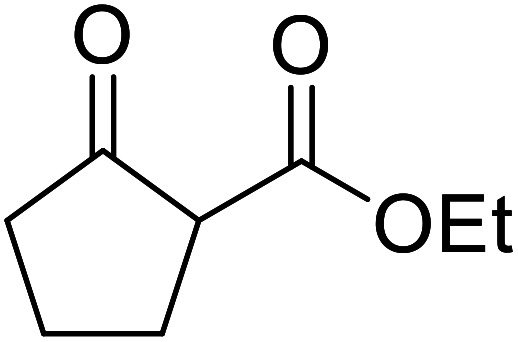	^*n*^Bu	FeSO_4_	2	77
			CuSO_4_	2.5	80
10	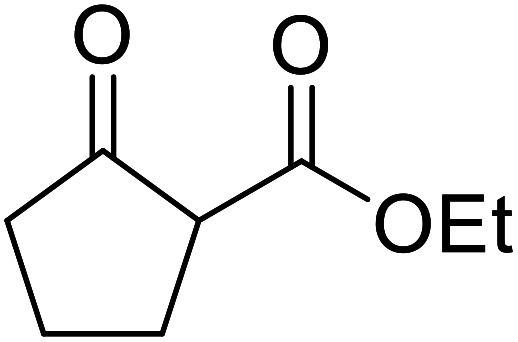	Menthyl	FeSO_4_	3	75
			CuSO_4_	3.5	77
11	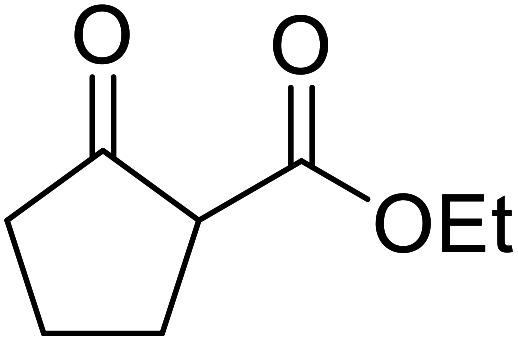	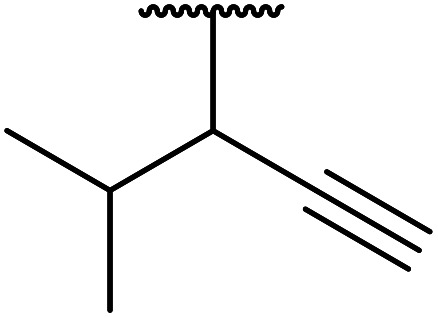	FeSO_4_	2.5	78
			CuSO_4_	3	80
12	Et	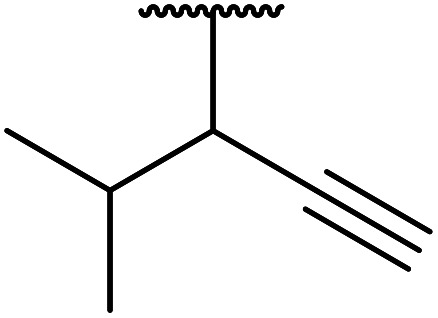	FeSO_4_	2	82
			CuSO_4_	2.5	80

**Scheme 6:**
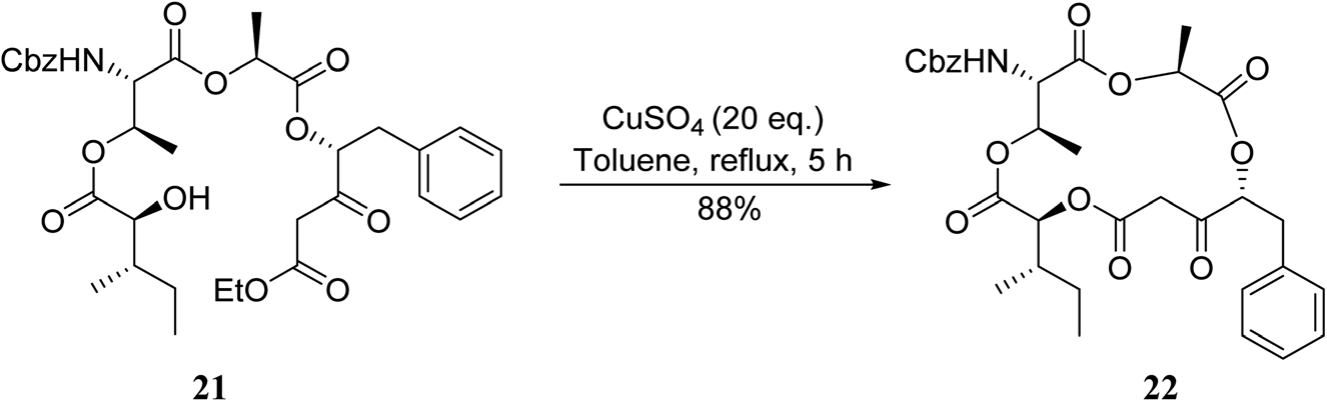
Copper-catalysed intramolecular transesterifications.

An application of this methodology can be seen in the synthesis of prunustatin A, a GRP78 molecular chaperone down-regulator, where the key step is a copper sulfate-mediated intramolecular transesterification to install the 15-membered tetralactone ring skeleton into 22 (Scheme 6).^3^

Magnetic copper ferrite nanoparticles (CuFe_2_O_4_) catalyse a variety of organic transformations^64^ and were shown to be similarly effective transesterification catalysts by Bezuidenhoudt and colleagues.^65^ The main attraction of these nanoparticles is their magnetic character, allowing for simple removal by a magnet. The scope of the reaction was evaluated by employing a variety of alcohols (Table 29). Both electron-withdrawing and electron-donating substituents on benzyl alcohol substrates (entries 2–5) gave comparable yields, although the less nucleophilic 4-nitrobenzyl alcohol required an additional two hours to reach completion (entry 2). The reaction of 2- and 4-methoxybenzyl alcohol (entries 5 and 3 respectively) proceeded smoothly despite the tendency of these substrates to undergo dehydroxylation *via* a benzylic carbocation intermediate.^66^ Aliphatic alcohols proved highly amenable and furnished the corresponding β-keto esters in excellent yields (entries 11–17). Propargyl alcohol (entry 9) and crotyl alcohol (entry 10) returned the lowest yields. The lower yields are likely due to tendency of the substrates to polymerise in the presence of acids. CuFe_2_O_4_ was successfully recycled eight times, with comparable results on each occasion.

**Table 29:** Copper iron oxide-catalysed transesterification of methyl acetoacetate^65^

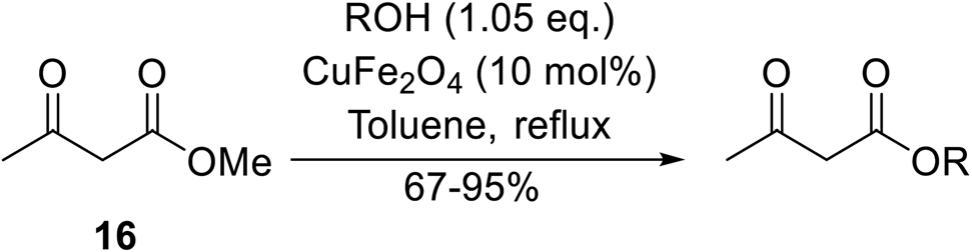			
Entry	R	Time (h)	Yield (%)
1	Bn	6	94
2	4-NO_2_–C_6_H_4_CH_2_	8	90
3	4-MeO–C_6_H_4_CH_2_	6	91
4	3-MeO–C_6_H_4_CH_2_	6	91
5	2-MeO–C_6_H_4_CH_2_	6	84
6	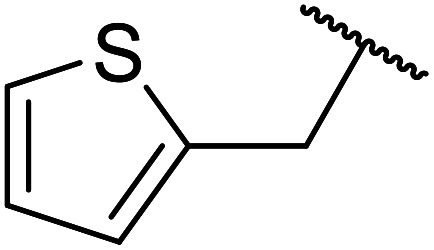	6	80
7	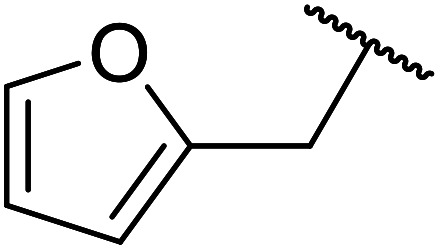	6	78
8	PhCH=CHCH_2_	8	85
9	HC≡CCH_2_	8	70
10	CH_3_CH=CHCH_2_	8	67
11	Cy	8	90
12	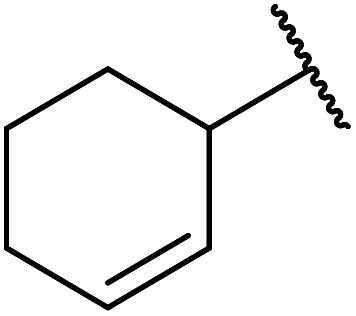	8	90
13	^*sec*^Bu	8	80
14	^*n*^Pen	8	95
15	^*n*^Hex	8	94
16	^*n*^Hep	8	92
17	^*n*^Oct	8	90

A ceramic catalyst, which consists of zirconia (ZrO_2_) with a yttria (Y_2_O_3_) stabilised cubic structure,^67^ accelerates the transesterification of β-keto esters with primary and secondary alcohols (Table 30).^68^ Conversion to a *tert*-butyl ester (entry 3), which is often problematic, was also realised by this route albeit in moderate yields. Similarly, unsaturated alcohols reacted smoothly affording the corresponding products in good yields (entries 4 and 7). While conversion from methyl/ethyl esters to higher homologues was facile, the reverse transformation (*e.g.*, menthyl ester to ethyl ester (entry 10)) could also be achieved in somewhat lower yields. This catalyst was selective for β-keto esters and other esters, such as α-keto esters, γ-keto esters, unsaturated esters or simple esters, failed to react. The conversion of β-keto esters to their thioester or amide derivatives, which often results in decarboxylation upon hydrolysis, proceeded in moderate yields (entries 11 and 12). Reaction occurred at the primary hydroxyls in the case of 1,2-diols, although a mixture of monoester and diester products was obtained (entry 13). In the case of 2-mercaptoethanol, the hydroxyl reacted preferentially over the thiol (entry 14). For aliphatic amino alcohols, reaction occurred exclusively at the amine in excellent yields (entry 15).

**Table 30:** Yttria–zirconia catalysis^68^

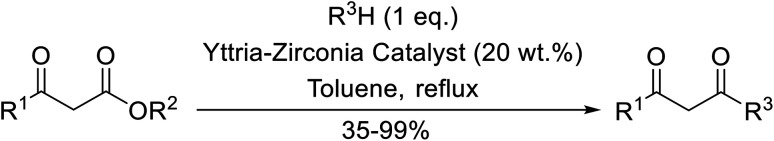					
Entry	R^1^	R^2^	R^3^	Time (h)	Yield (%)
1	Me	Me	^*n*^BuO	10	67
2	Me	Me	(−)-Menthol	18	98
3	Me	Me	^*t*^BuO	15	39
4	Me	Me	HC≡CCH_2_O	15	67
5	Me	Me	BnO	14	99
6	Me	Me	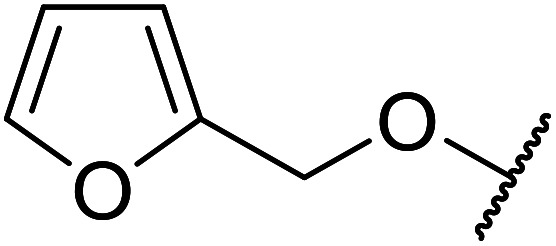	13	95
7	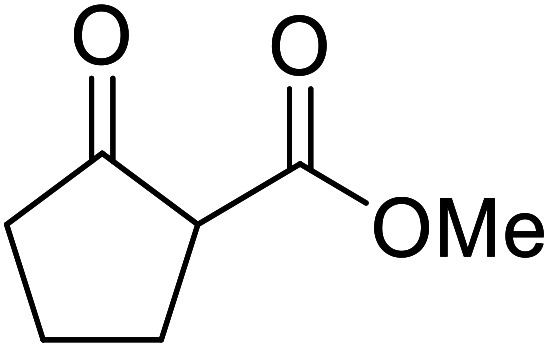		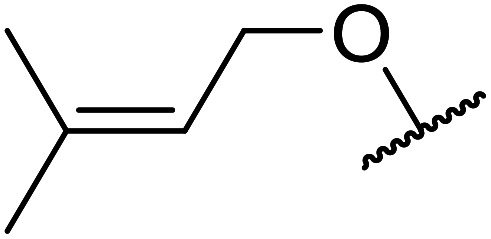	6	82
8	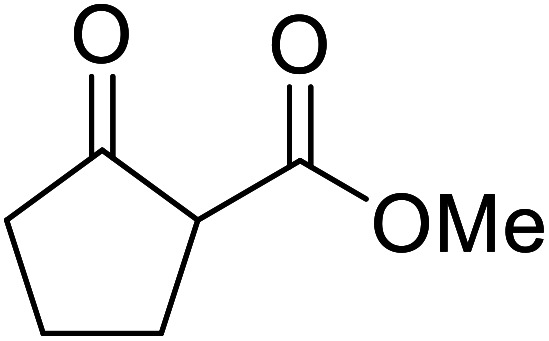		CyO	8	94
9	Ph	Et	^*n*^HexO	10	93
10	Me	Menthyl	EtO	18	35
11	Me	Me	PhS	10	42
12	Me	Me	PhNH	3	59
13	Me	Me	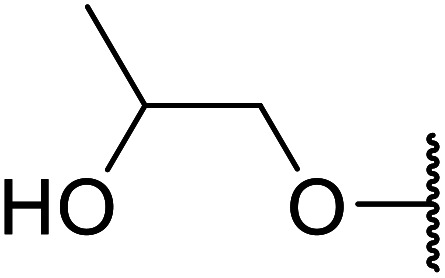	8	68^a^
14	Me	Me	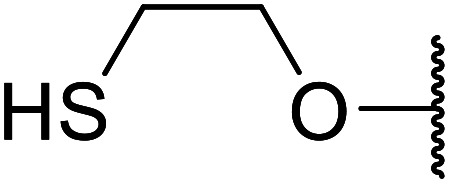	9.5	86
15	Me	Me	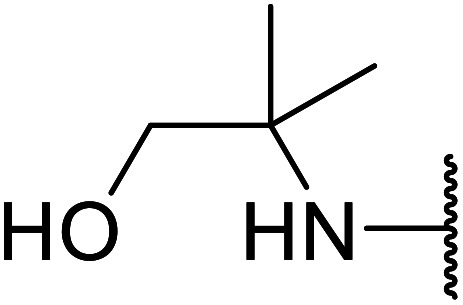	10	91

a 15% di-transesterification product isolated.

Krishnaiah *et al.* identified Mn(ii) salts as efficient catalysts for the selective transesterification of β-keto esters with various alcohols (Table 31).^69,70^ Long reaction times were required for Mn(ii) sulfate and Mn(ii) carbonate, even at reflux temperatures, but good yields were eventually obtained. The use of ultrasonic conditions reduced reaction times from 16–24 hours to 2.5 hours, while microwave-mediated transformations reached completion within 45 minutes. A loading of 1 equivalent was sufficient across a wide range of alcohols.

**Table 31:** Mn(ii), Cs(ii) and Prussian blue catalysis^69–71^

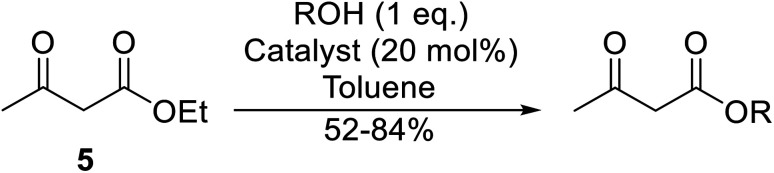								
Entry	R	Catalyst	Conventional^a^		Ultrasonic^b^		Microwave^c^	
			Time (h)	Yield (%)	Time (h)	Yield (%)	Time (h)	Yield (%)
1	Bn	MnSO_4_	18	72	2	73	0.75	72
		MnCO_3_	18	75	2.15	74	0.75	74
		CsCO_3_	20	75	1	74	0.1	84
		(Fe_4_(Fe(CN)_6_)_3_·*y*H_2_O)	19	75	2.15	74	0.13	84
2	Tol	MnSO_4_	24	76	3.5	74	0.83	73
		MnCO_3_	24	73	3.5	75	0.83	75
		CsCO_3_	20	72	1	74	0.1	78
		(Fe_4_(Fe(CN)_6_)_3_·*y*H_2_O)	19	72	2.15	74	0.16	78
3	PhCH=CHCH_2_	MnSO_4_	24	76	3.15	73	0.83	72
		MnCO_3_	24	72	3.15	71	0.83	75
		CsCO_3_	20	75	1	75	0.1	80
		(Fe_4_(Fe(CN)_6_)_3_·*y*H_2_O)	20	75	2.15	75	0.16	80
4	Ph	MnSO_4_	24	68	3.5	67	0.75	70
		MnCO_3_	24	69	3.15	71	0.75	69
		CsCO_3_	20	75	1	78	0.1	78
		(Fe_4_(Fe(CN)_6_)_3_·*y*H_2_O)	24	68	2.15	70	0.16	75
5	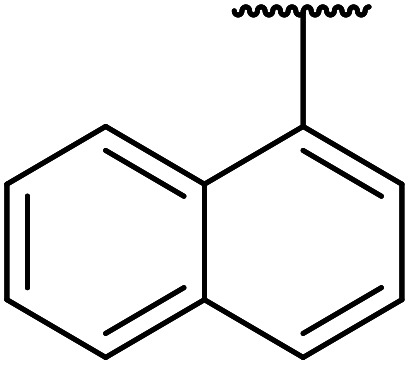	MnSO_4_	24	62	3.5	60	0.75	65
		MnCO_3_	24	60	3.4	63	0.75	65
		CsCO_3_	20	66	1	73	0.1	80
		(Fe_4_(Fe(CN)_6_)_3_·*y*H_2_O)	24	66	2.0	73	0.2	80
6	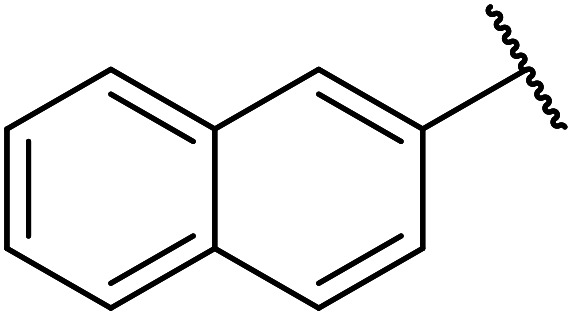	MnSO_4_	24	58	3.3	61	0.91	64
		MnCO_3_	24	63	3.3	64	0.91	67
		CsCO_3_	20	68	1	74	0.1	75
		(Fe_4_(Fe(CN)_6_)_3_·*y*H_2_O)	24	68	2.15	74	0.18	75
7	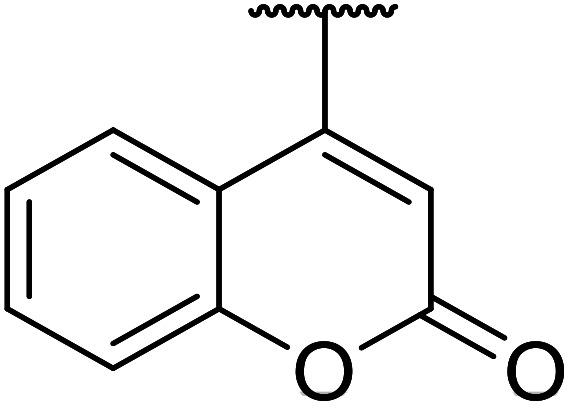	MnSO_4_	24	57	2.5	59	0.66	65
		MnCO_3_	24	52	2.5	55	0.66	69
		CsCO_3_	20	66	1	72	0.1	80
		(Fe_4_(Fe(CN)_6_)_3_·*y*H_2_O)	22	66	2.15	72	0.16	80
8	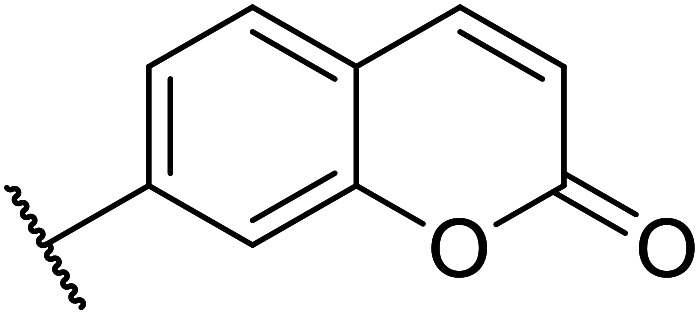	MnSO_4_	24	58	2.3	59	0.58	68
		MnCO_3_	24	53	2.4	61	0.58	65
		CsCO_3_	20	70	1	72	0.1	75
		(Fe_4_(Fe(CN)_6_)_3_·*y*H_2_O)	22	70	2.15	72	0.16	75
9	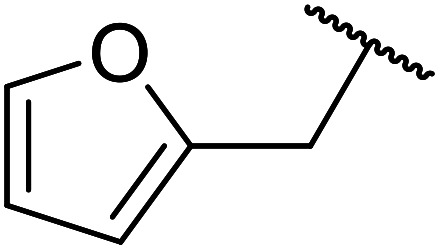	MnSO_4_	18	60	2.15	69	0.63	68
		MnCO_3_	18	60	2.15	65	0.66	65
		CsCO_3_	20	75	1	75	0.1	80
		(Fe_4_(Fe(CN)_6_)_3_·*y*H_2_O)	19	75	3.0	75	0.13	80
10	^i^Pr	MnSO_4_	15	68	3	73	0.75	72
		MnCO_3_	15.5	69	3.15	72	0.70	70
		CsCO_3_	20	70	1	72	0.1	80
		(Fe_4_(Fe(CN)_6_)_3_·*y*H_2_O)	19	70	3.0	72	0.13	8
11	^*n*^Pr	MnSO_4_	16	71	3	75	0.91	75
		MnCO_3_	16	71	3	79	0.90	75
		CsCO_3_	20	75	1	75	0.1	82
		(Fe_4_(Fe(CN)_6_)_3_·*y*H_2_O)	20	75	2.45	75	0.13	82
12	^*n*^Pen	MnSO_4_	16	63	3.15	67	0.83	70
		MnCO_3_	16	64	3	66	0.83	70
		CsCO_3_	20	78	1	75	0.1	80
		(Fe_4_(Fe(CN)_6_)_3_·*y*H_2_O)	20	78	3.0	75	0.15	80
13	^*t*^Bu	MnSO_4_	19	58	2.15	60	0.66	57
		MnCO_3_	19	60	2.15	60	0.66	58
		CsCO_3_	20	65	1	70	0.1	75
		(Fe_4_(Fe(CN)_6_)_3_·*y*H_2_O)	24	65	3.15	70	0.2	75
14	Cy	MnSO_4_	16	67	2.15	72	0.7	68
		MnCO_3_	16	65	2.3	75	0.7	70
		CsCO_3_	20	72	1	72	0.1	80
		(Fe_4_(Fe(CN)_6_)_3_·*y*H_2_O)	23	72	3.15	72	0.16	80
15	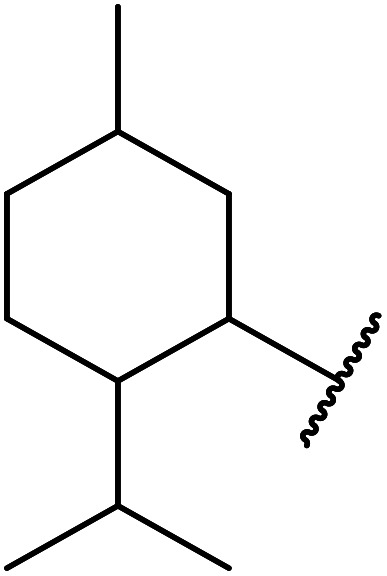	MnSO_4_	18	57	2.15	69	0.66	70
		MnCO_3_	18	54	2.15	70	0.66	75
		CsCO_3_	20	70	1	75	0.1	82
		(Fe_4_(Fe(CN)_6_)_3_·*y*H_2_O)	23	70	3.10	75	0.16	82

a Conventional conditions: 100–110 °C.

b Ultrasonic conditions: sonicator bath, r.t.

c Microwave conditions: MW irradiation source consisting of magnetron tube operating at 2.45 GHz.

Further work within the same group confirmed caesium carbonate to be an effective catalyst under conventional, microwave and ultrasonically-assisted conditions.^70^ Infrared studies suggested that the Brønsted base facilitates generation of an enolate intermediate. As alkoxides are generally more nucleophilic than alcohols, the most likely mechanism proceeds by deprotonation of the alcohol to form an alkoxide nucleophile which then attacks the β-keto ester. Less sterically hindered primary alcohols reacted faster and returned higher yields compared to secondary or tertiary alcohols.

Prussian blue (iron hexacyanoferrate) is a frequently employed blue-coloured pigment which is also an effective transesterification catalyst.^71^ It exists in water soluble (KFe_4_(Fe(CN)_6_)·*x*H_2_O) and water insoluble (Fe_4_(Fe(CN)_6_)_3_·*y*H_2_O) forms and its microporous character makes it an ideal catalytic agent.^72^ A HSAB theory approach supports a reaction mechanism similar to caesium carbonate where iron behaves as a hard acid to form an adduct with ethyl acetoacetate.

All three catalysts returned similar yields with comparable reaction times.

### 5.2 Non-transition metals

**Scheme 7:**
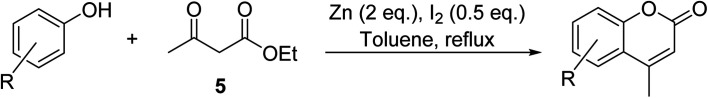
Pechmann condensation using zinc and iodine.

A zinc/iodine-catalysed transesterification of methyl acetoacetate with primary and secondary alcohols was developed by Chavan and colleagues (Table 32).^73^ It was not possible to transesterify tertiary alcohols under these conditions, possibly due to the increased steric bulk. Introducing a diol led to the crosslinking of esters to generate the doubly transesterified product (entry 10). Notably, 4-methylcoumarins were recovered as the major product when phenols were employed as the nucleophile. These were likely the result of a Pechmann condensation (Scheme 7).

**Table 32:** Zinc/iodine catalysis^73^

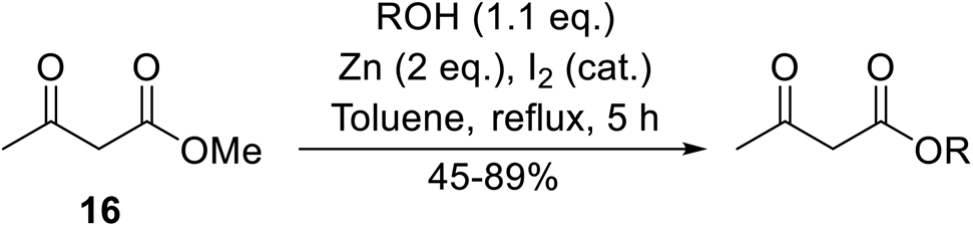		
Entry	R	Yield (%)
1	^*t*^Bu	85
2	^i^Pr	62
3	Bn	66
4	^*n*^Bu	78
5	H_2_C=CHCH_2_	45
6	Cy	60
7	HC≡CCH_2_	71
8	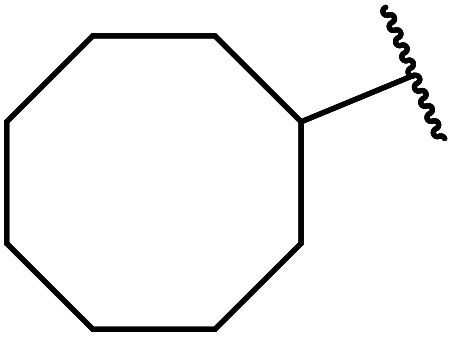	79
9	(−)-Menthol	89
10	HO(CH_2_)_5_	66^a^

a 2 eq. of methyl acetoacetate were used. Diester product isolated.

**Fig. 4:**
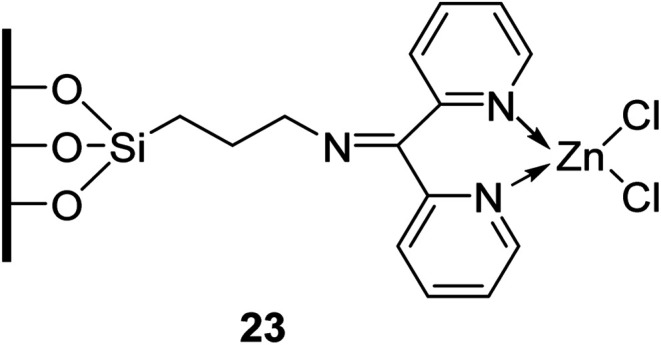
Zinc chloride immobilised on functionalised silica.

Homogenous catalysts are often characterised by poor chemical and thermal stability which makes catalyst separation and recycling challenging. A strategy to overcome this is to covalently incorporate an organic entity onto inorganic solids. The ideal immobilised catalyst would possess easily accessible active sites which are well dispersed on its surface. Generally, this requires the support to have a reasonably high surface area (typically >100 m^2^ g^−1^).^74^ Sharma and Rawat have developed a recyclable silica-based inorganic–organic hybrid zinc catalyst (Fig. 4).^75^ They coupled a modified silica gel to zinc chloride to create an inorganic–organic interphase catalyst, thus allowing for subsequent separation and recycling. Surface area analysis of this hybrid catalyst revealed a surface area of 116.6 m^2^ g^−1^. Transesterification proceeded smoothly in the presence of this catalyst in toluene at refluxing conditions (Table 33). The immobilized catalyst displayed higher catalytic activity compared to the homogeneous zinc chloride and was reused with no loss in catalytic activity (entry 1).

**Table 33:** Zinc on silica catalysis^75^

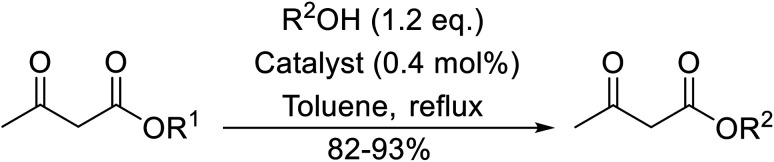				
Entry	R^1^	R^2^	Time (h)	Yield (%)
1	Et	^*n*^Bu	4.0	92 (60)^a^
2	Et	^*sec*^Pen	4.5	88
3	Et	Cy	4.0	90
4	Et	Bn	4.5	89
5	Me	H_2_C=CHCH_2_	4.5	90
6	Et	PhHC=CHCH_2_	5.0	82
7	Me	^*n*^Pr	4.0	93

a Homogenous catalyst used.

Chemoselective transesterifications employing zinc oxide were investigated by Vallribera and co-workers (Table 34).^76^ Reactions reached completion within 24 hours or less, with the exception of isopropanol (entry 2) and *tert-*butanol (entry 5) which required 48 and 72 hours respectively. In the case of menthol, changing the stoichiometry from 10 equivalents to 1.5 equivalents afforded comparable yields (entry 10). Both α-keto esters (entry 13) and simple esters (entry 14) failed to react under these conditions. Further studies indicated that the reaction is selective for β-keto esters, as substrates containing both ester and β-keto ester functionalities reacted exclusively at the β-keto ester site (entry 15). More recently, Soliman *et al.* successfully optimised the transesterification of α-keto esters and simple esters utilising zinc oxide nanoparticles, achieving yields up to 97%.^77^ The modification of both the structure and morphology of the oxide significantly improved activity.

**Table 34:** Zinc oxide-catalysed transesterification^76^

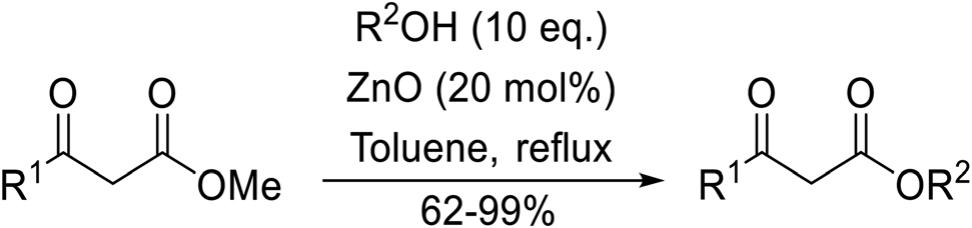				
Entry	R^1^	R^2^	Time (h)	Yield (%)
1	3,4-(MeO)_2_–C_6_H_3_	^i^Bu	5	93
2	3,4-(MeO)_2_–C_6_H_3_	^i^Pr	48	93
3	3,4-(MeO)_2_–C_6_H_3_	(Et)_2_CH	6	99
4	3,4-(MeO)_2_–C_6_H_3_	(^i^Pr)_2_CH	1	96
5	3,4-(MeO)_2_–C_6_H_3_	^*t*^Bu	72	62
6	3,4-(MeO)_2_–C_6_H_3_	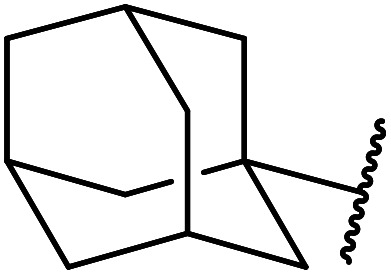	24	87^a^
7	3,4-(MeO)_2_–C_6_H_3_	Bn	2	82
8	3,4-(MeO)_2_–C_6_H_3_	H_2_C=CHCH_2_	3.5	90
9	3,4-(MeO)_2_–C_6_H_3_	HC≡CCH_2_	5	81
10	3,4-(MeO)_2_–C_6_H_3_	(+)-Menthyl	1	99 (92)^b^
11	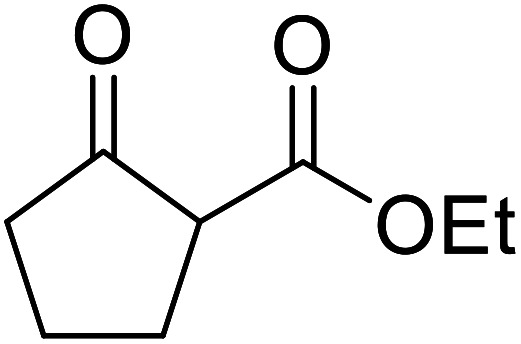	(Et)_2_CH	2	98
12	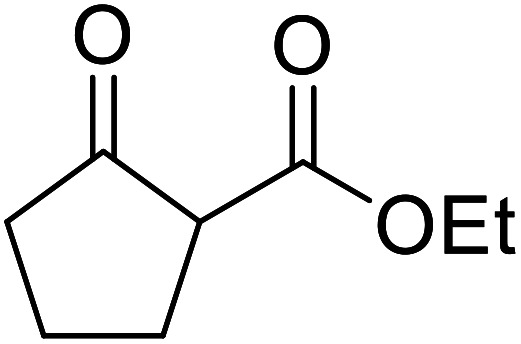	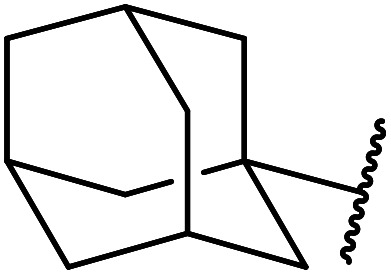	5	83
13	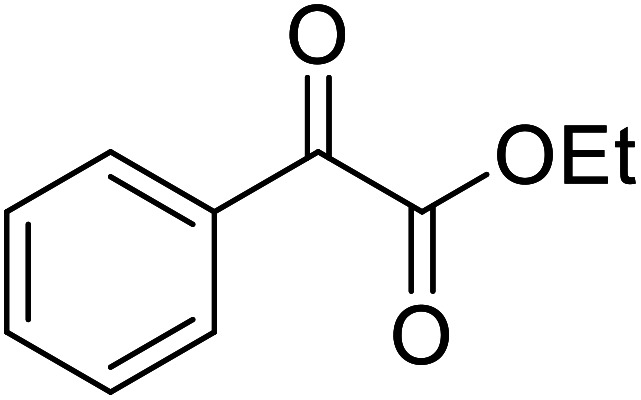	(^i^Pr)_2_CH	24	n. r.
14	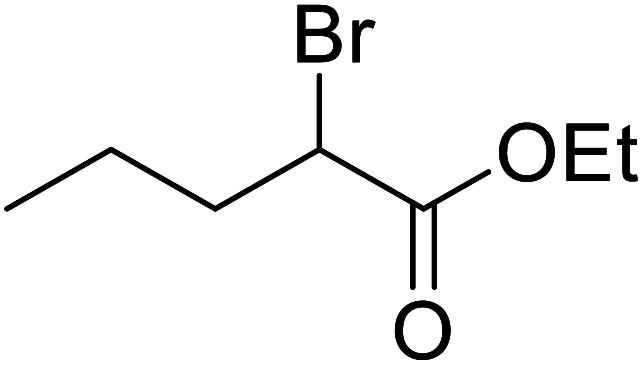	(^i^Pr)_2_CH	24	n. r.
15	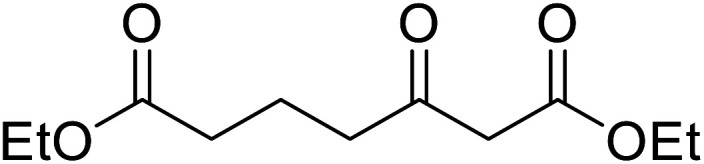	(^i^Pr)_2_CH	2	92

a 1.3 eq. ROH.

b 1.5 eq. menthol.

The successful transesterification of β-keto esters using primary (entries 1, 4–6, 9, 11, 13 and 15), secondary (entries 2, 8, 10, 14 and 16), tertiary (entries 3, 7 and 12), allylic (entries 4, 11, 13 and 15) and benzylic (entry 1) alcohols with catalytic amounts of zinc sulfate at 80 °C was reported by Bandgar and colleagues (Table 35)*.*^78^ The nature of the alcohol had a significant effect on reaction kinetics, with reaction times ranging from 5.5 hours (entry 2) to 14 hours (entry 3) with the same β-keto ester. A drawback of this catalyst was the requirement for a relatively high catalyst loading of 20 mol%.

**Table 35:** Zinc sulfate catalysis^78^

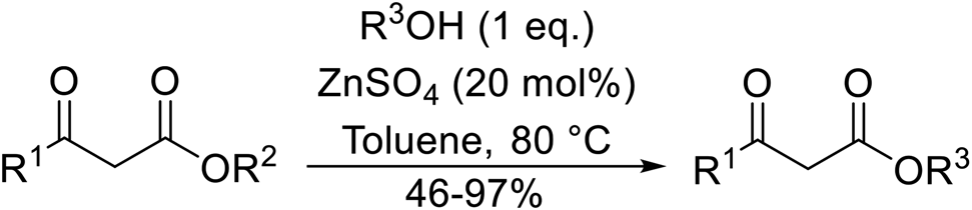						
Entry	R^1^	R^2^		R^3^	Time (h)	Yield (%)
1	Me	Me		Bn	2.75	89
2	Me	Me		(−)-Menthyl	5.5	95
3	Me	Me		^*t*^Bu	14	78
4	Me	Me		PhCH=CHCH_2_	4.25	97
5	Me	Me		^*n*^Bu	5.5	91
6	Me	Me		HO(CH_2_)_2_	6	81^a^
7	Me	Et		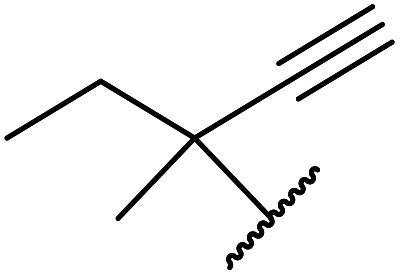	11.25	58
8	Me	Et		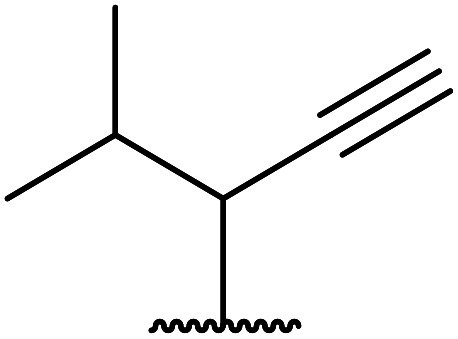	8	92
9	Me	Me		Et	6	87
10	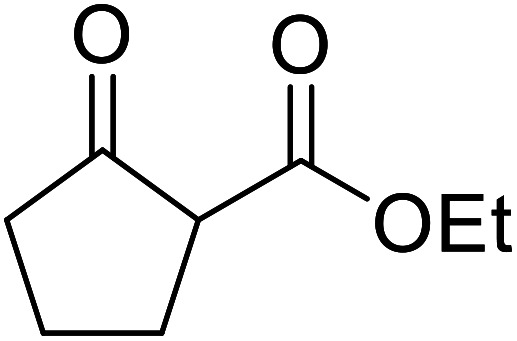			(−)-Menthyl	1	93
11	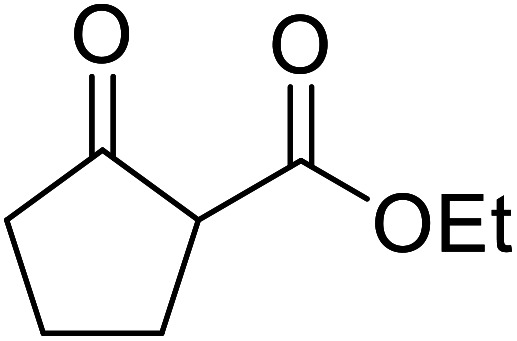			PhCHCHCH_2_	1	81
12	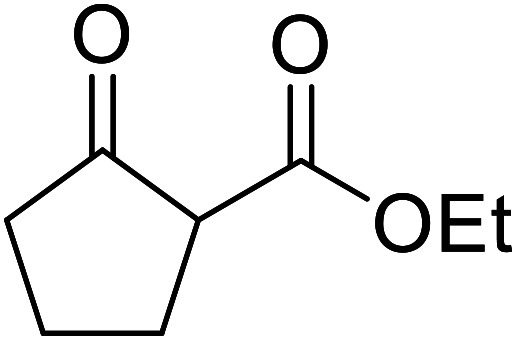			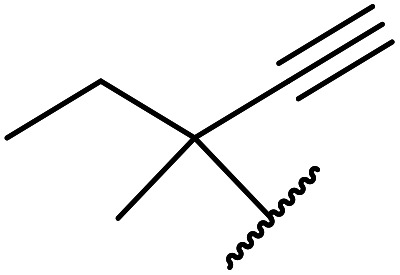	12	46
13	3,4,5-(MeO)_3_–C_6_H_2_		Et	PhCH=CHCH_2_	11	95
14	3,4,5-(MeO)_3_–C_6_H_2_		Et	(−)-Menthyl	7.5	66
15	Ph		Et	PhCH=CHCH_2_	4	72
16	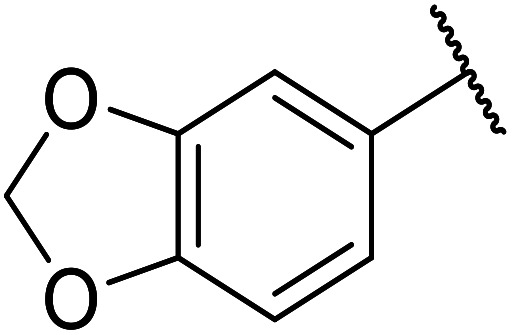		Et	(−)-Menthyl	6	82

a Di-transesterification product.

**Fig. 5:**
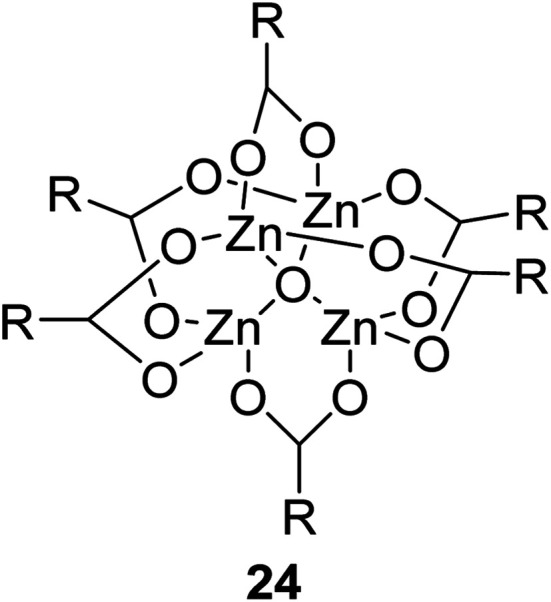
General structure of tetra-nuclear zinc clusters.

The synthesis of an acetate-bridged tetra-nuclear zinc cluster was pioneered for the first time by Auger and Robin in 1924 *via* vacuum distillation of zinc acetate hydrate (Fig. 5).^79^ Other tetra-nuclear zinc clusters with different carboxylate ligands, such as Zn_4_(OCOR)_6_O (where R = Et, *n-*Pr, *t-*Bu, Ph, *etc.*), were subsequently prepared using similar synthetic or pyrolytic methods.^80^

Extensive studies on the application of these zinc clusters in organic transformations have been reported by Ohshima and colleagues.^81–85^ Zinc cluster catalysts accelerate the transesterification of both simple methyl esters and β-keto methyl esters (Table 36). Even sterically congested alcohols such as adamantyl (entry 18) and menthyl alcohol (entry 16) reacted smoothly, with no elimination side-products observed. α-Methyl-substituted β-keto ester was converted to the transesterified product in only moderate yields, even after extended reaction times (entry 3), but β-keto esters derived from 5- or 6-membered cyclic ketones were readily transformed in high yields (entries 4 and 5). At reflux, cinnamyl alcohol underwent transesterification to the desired allylic ester but the formation of 1,3-diphenyl-1-oxo-4-pentene was also noted. These side reactions could be avoided by reducing the reaction temperature to 69 °C (entry 10). Alcohols containing pivaloyl ester (entry 12), silylether (entry 13) or tetrahydropyran (entry 14) protecting groups reacted smoothly, affording the corresponding esters in high yields. Mildly acidic phenol was unreactive, likely due to the formation of the inactive zinc phenoxide species (entry 20).^82^ This zinc catalyst was highly compatible with dicarbonyl derivatives of β-keto esters, leading to double transesterification (entries 6 and 7). In the case of dimethyl malonate and Meldrum's acid, both ester groups were transformed using 2.6 equivalents of alcohol to give dihexyl malonate in good yields (entries 6 and 7). When methyl-*N*-hexyl malonamide was investigated, only the ester group reacted, leaving the remaining amide intact (entry 8). While a highly acid-sensitive tetrahydropyranyl (THP) ether group was prone to degradation, the addition of 10 mol% of 4-DMAP suppressed decomposition to furnish a 94% yield (entry 14).

**Table 36:** Zinc cluster catalysis^81,84^

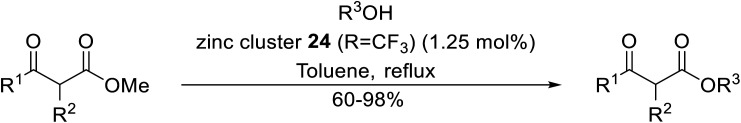						
Entry	R^1^	R^2^	R^3^	Alcohol (eq.)	Time (h)	Yield (%)
1	Me	H	^*n*^Hex	1.2	48	81
2	^*t*^Bu	H	^*n*^Hex	1.2	48	81
3	Ph	Me	^*n*^Hex	1.2	48	60
4	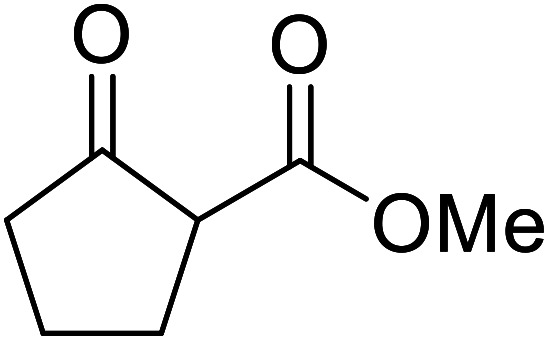		^*n*^Hex	1.2	42	87
5	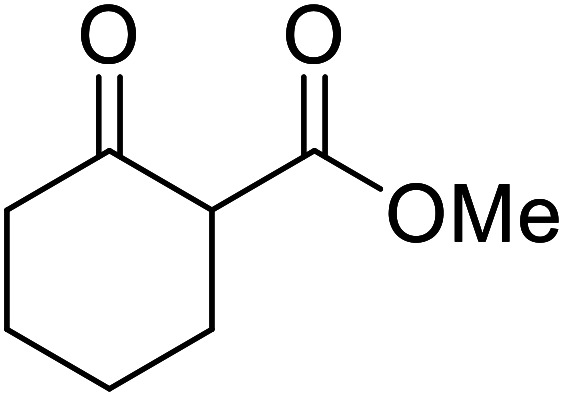		^*n*^Hex	1.2	48	97
6	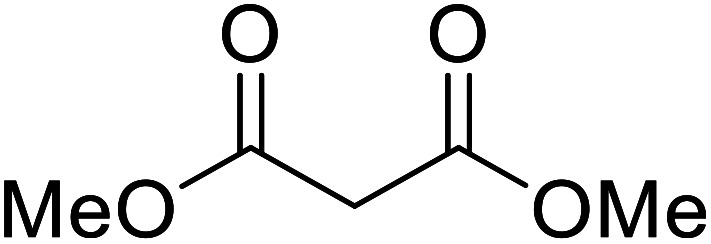		^*n*^Hex	2.6	68	98
7	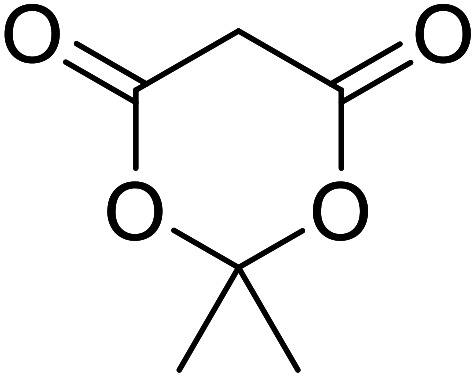		^*n*^Hex	2.6	68	73^a^
8	^*n*^Hex-NH	H	^*n*^Hex	1.2	60	77
9	Ph	H	^*t*^BuCH_2_	1.2	44	89
10	Ph	H	PhCH=CHCH_2_	1.2	65	86^b^
11	Ph	H	CH_3_C≡CCH_2_	1.2	48	82
12	Ph	H	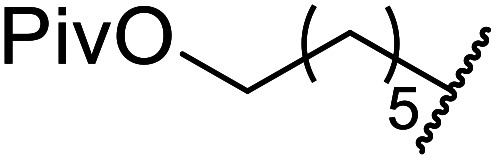	1.2	60	89
13	Ph	H	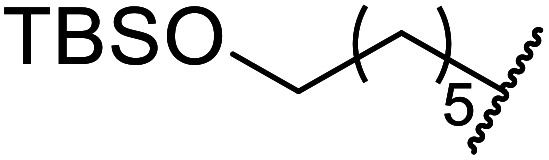	1.2	48	86
14	Ph	H	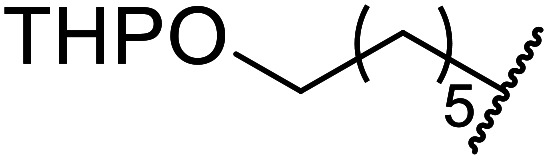	1.2	48	94^c^
15	Ph	H	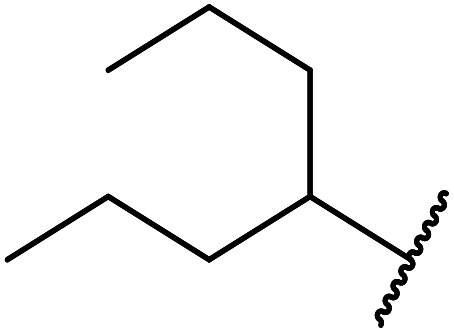	1.2	44	87
16	Ph	H	(−)-Menthyl	1.2	44	93
17	Ph	H	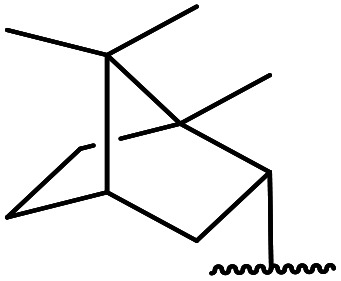	1.5	44	97
18	Ph	H	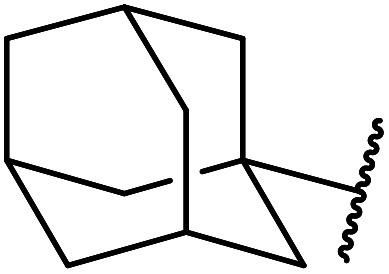	2.0	72	85
19	Ph	H	^*t*^Bu	5.0	72	82
20	Ph	H	Ph	1.2	45	n. r.
21	Ph	H	Bn	1.2	45	89
22	Ph	H	^*n*^Hex	1.2	48	86
23	Ph	H	Cy	1.2	45	95

a Dihexyl malonate isolated as major product.

b ^i^Pr_2_O was used as the solvent.

c 10 mol% of 4-DMAP was added.

An extremely rapid transesterification methodology was developed by Goswami *et al.* using the environmentally benign and inexpensive catalyst Al(H_2_PO_4_)_3_.^86^ The significance of the catalyst lies in its ease of preparation. Upon heating Al(H_2_PO_4_)_3_ to 200 °C, acidic aluminium triphosphate (AlH_2_P_3_O_10_)·2H_2_O is formed, which condenses to give cyclic hexametaphosphate Al_2_P_6_O_18_.^87^ The catalyst can be recovered *via* filtration and reactivated by heating to 200–220 °C for 30 minutes without loss of activity. As can be seen from Table 37, long chain alkyl (entries 4 and 5), cyclic (entries 7–9), benzylic (entries 10–13) and, notably, allylic (entry 1–3) alcohols reacted rapidly in good yields, despite the extremely low catalyst loadings. Methyl acetoacetate afforded doubly transesterified products on reaction with various diols (entries 14 and 15). Unfortunately, tertiary alcohols did not react even after prolonged reaction times. The catalyst is selective for β-keto esters and α-keto esters. The reaction likely proceeds by way of an acyl ketene intermediate as equimolar amounts of methyl acetoacetate and ethyl benzoylacetate afforded methyl benzoylacetate and ethyl acetoacetate respectively *via* exchange of the alcohol fragments of both β-keto esters.^10^

**Table 37:** Al(H_2_PO_4_)_3_ catalysis^86^

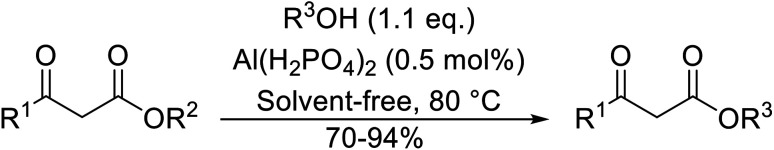					
Entry	R^1^	R^2^	R^3^	Time (min)	Yield (%)
1	Me	Me	H_2_C=CHCH_2_	10	93
2	Me	Me	CH_3_CH=CHCH_2_	15	90
3	Me	Me	CH_3_CH=CHCH_2_CH=CHCH_2_	20	91
4	Me	Me	^*n*^Dec	20	89
5	Me	Me	CH_3_(CH_2_)_15_	25	82
6	Me	Me	Cy	20	94
7	Me	Me	(−)-Menthyl	20	85
8	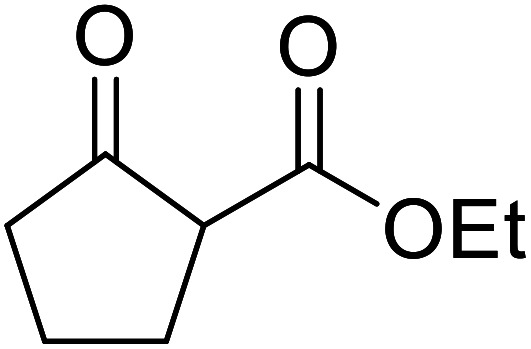		(−)-Menthyl	20	89
9	Et	Et	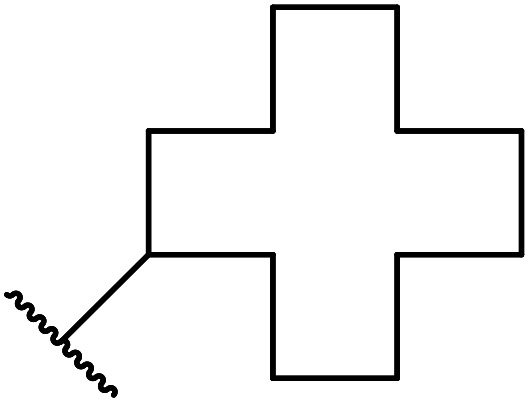	15	88
10	Me	Me	Bn	20	92
11	Me	Me	Ph(CH_2_)_3_	15	88
12	Me	Me	Ph(CH_2_)_4_	20	90
13	Me	Me	5-NO_2_–C_6_H_4_CH_2_	20	91
14	Me	Me	HO(CH_2_)_8_	30	81^a^
15	Me	Me	HOCH_2_C≡CCH_2_	30	70^a^

a Diester isolated.

Having demonstrated the catalytic properties of Mg–Al–O–^*t*^Bu hydrotalcite for aldol condensations and the epoxidation of olefins,^88,89^ Choudary and colleagues subsequently investigated its utility for transesterifications (Table 38).^90^ Unsaturated alcohols, such as allyl alcohol (entry 5) and cinnamyl alcohol (entries 1 and 2), were converted in high yields with no decarboxylative Carroll rearrangement observed. The catalytic system also accelerated transamidations (entry 7). The catalyst is not selective for β-keto esters as simple esters were also susceptible to reaction under these conditions (entries 3 and 13).

**Table 38:** Mg–Al–O–^*t*^Bu hydrotalcite catalysis^90^

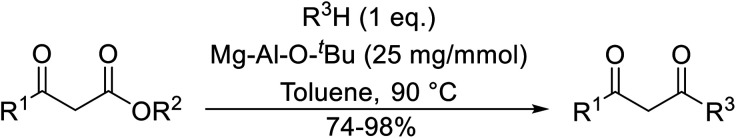					
Entry	R^1^	R^2^	R^3^	Time (h)	Yield (%)
1	Me	Et	PhCH=CHCH_2_O	2	74
2	Ph	Me	PhCH=CHCH_2_O	2	90
3	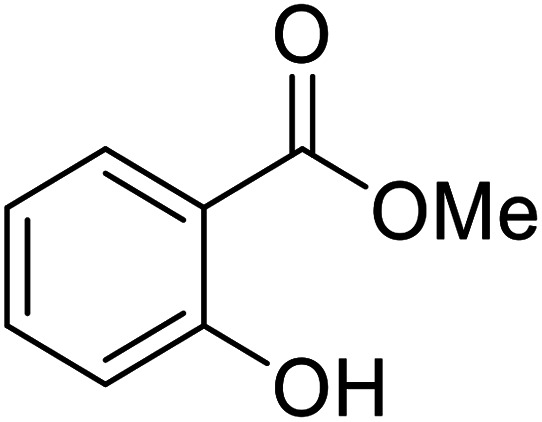		BnO	3	98
4	Me	Et	CyO	2	97
5	Me	Me	H_2_C=CHCH_2_O	1.5	95
6	Me	Me	^*n*^PrO	1	96
7	Me	Me	PhNH	2	95
8	Me	Me	CyO	2	98
9	Me	Et	^*n*^PenO	3	97
10	Me	Et	^*n*^OctO	2.5	97
11	Me	Me	(−)-Menthyl	2	90
12	Me	Me	^*n*^HexO	2	98 (97)^a^
13	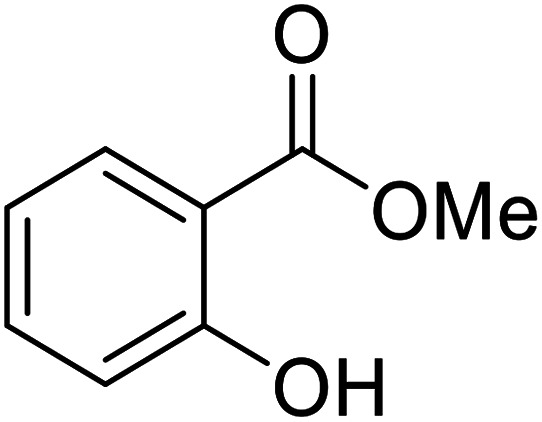		^*n*^OctO	2	92

a Yield after 6th cycle.

Bismuth halides are relatively non-toxic, inexpensive and easily handled. Due to the weak shielding of the 4f electrons (lanthanide contraction), bismuth(iii) compounds exhibit Lewis acidity.^91,92^ Bismuth chloride facilitates many different transformations including aza-Diels–Alder reactions and one-pot Biginelli reactions.^93,94^ The bismuth chloride-catalysed transesterification of a range of methyl and ethyl β-keto esters proceeded smoothly with aliphatic (entries 1 and 3), allylic (entries 7 and 12), propargyl (entry 6) and aromatic (entries 2, 4, 5, 8, 10 and 11) alcohols in good to excellent yields (Table 39).^95^ Comparable yields were recorded regardless of the nature of the substituents on the β-keto ester substrates.

**Table 39:** Bismuth(iii) chloride-catalysed transesterification^95^

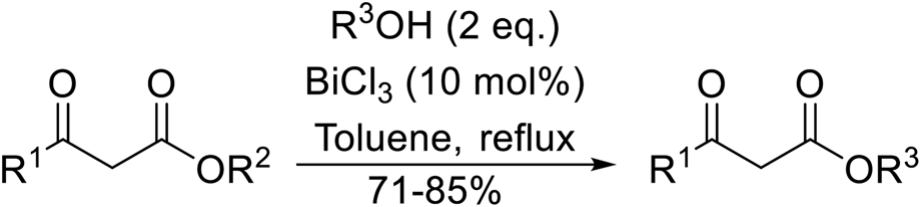					
Entry	R^1^	R^2^	R^3^	Time (h)	Yield (%)
1	Me	Me	Bu	3	85
2	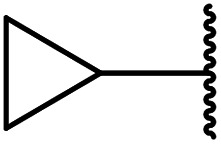	Me	Bn	4	75
3	^*t*^Bu	Et	(+)-Menthyl	4.5	71
4	^i^Pr	Et	Bn	3	72
5	^*t*^Bu	Et	Bn	3.5	78
6	Ph	Et	HC≡CCH_2_	3	81
7	^i^Pr	Et	H_2_C=CH(CH_2_)_2_	3	79
8	^i^Pr	Et	Ph(CH_2_)_3_	2.5	84
9	^i^Pr	Et	(−)-Menthyl	4.5	73
10	Ph	Et	Bn	3	85
11	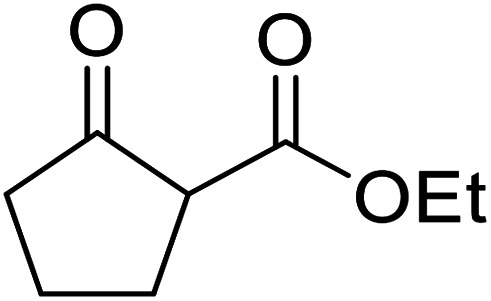		Bn	4	82
12	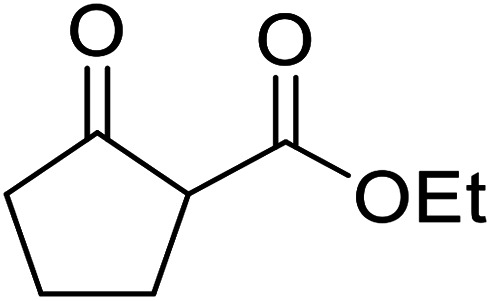		H_2_CCHCH_2_	4.5	85

## 6. Lanthanide catalysts

The catalytic potential of lanthanide catalysts has yet to be fully realised, with only limited examples of their use in synthetic chemistry currently available. The successful application of a ceria–yttria-based Lewis acid (CeO_2_–Y_2_O_3_) as an acylation catalyst has recently been reported.^96^ Transesterification of β-keto esters using this system was found to proceed in good to excellent yields (Table 40).^97^ Primary (entries 1, 3, 5–7, 10–12), secondary (entries 2 and 8), tertiary (entry 4), unsaturated (entry 9) and aromatic alcohols (entries 5 and 6) were readily transformed under these conditions. As expected, a reduction in yield was observed with bulky nucleophiles (entry 9). Conversion from methyl/ethyl esters to higher homologues proved straightforward. By contrast, the reverse transformation (*e.g.*, menthyl ester to ethyl ester), afforded only moderate yields (entry 11). α-Keto esters and γ-keto esters were not compatible with this system. The catalyst may be reactivated by heating to 500 °C in air without loss of activity.

**Table 40:** Transesterification catalysis using a ceria-yttria-based Lewis acid^97^

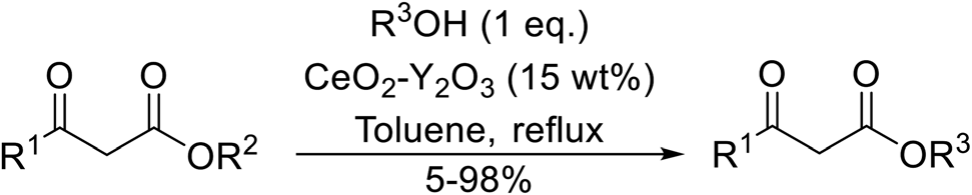					
Entry	R^1^	R^2^	R^3^	Time (h)	Yield (%)
1	Me	Me	^*n*^Hex	10	95
2	Me	Me	Cy	8	95
3	Me	Me	Me	9	98
4	Me	Me	^*t*^Bu	15	30
5	Me	Me	Bn	10	95
6	Me	Me	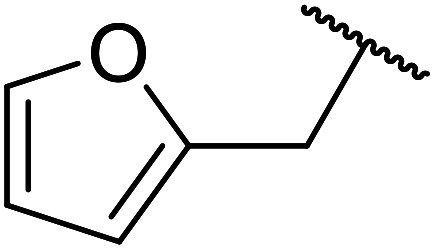	15	65
7	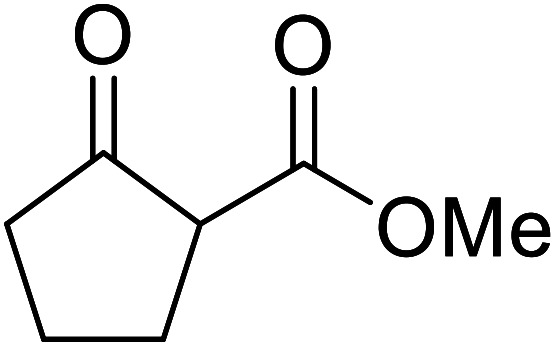		^*n*^Bu	9	82
8	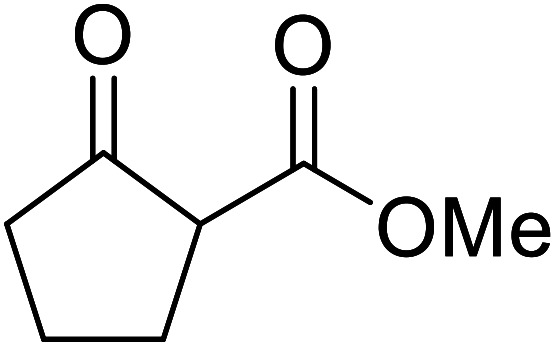		Cy	8	97
9	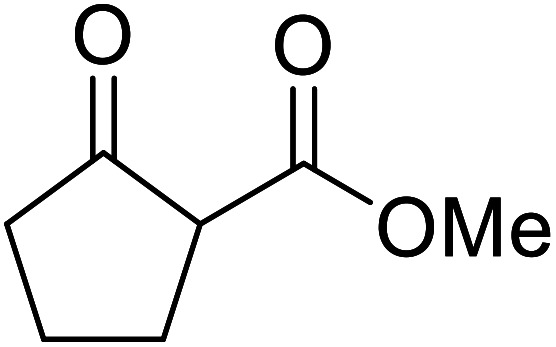		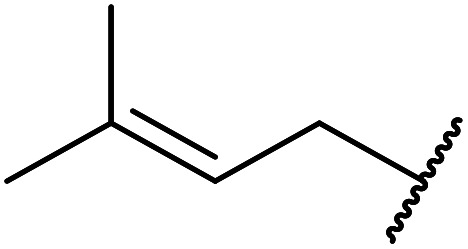	10	85
10	Ph	Me	^*n*^Hex	8	93
11	Me	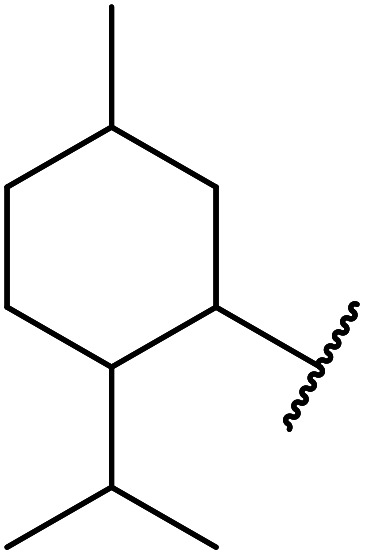	Et	24	5 (18^a^)
12	Me	Me	^*n*^Bu	8	95

a EtOH used as solvent.

Ytterbium(iii) triflate is an established Lewis acid catalyst for various organic transformations.^98^ Primary (Table 41 entries 1–5, and 10–14) and secondary alcohols (entries 6–8) were rapidly converted to the corresponding β-keto esters. A reduced yield was recorded in the case of tertiary alcohols, due to steric hindrance (entry 9). The catalyst is selective for alcohols over amines (entry 5), which is unexpected, as usually the more nucleophilic amine reacts preferentially.

**Table 41:** Ytterbium triflate-mediated transesterifications^98^

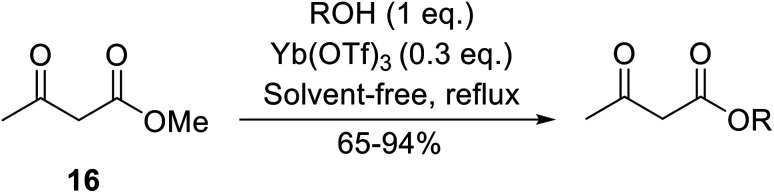			
Entry	R	Time (h)	Yield (%)
1	Et	3	94
2	^*n*^Bu	3	94
3	(CH_3_)_2_CHCH_2_	3	90
4	^*n*^Oct	3	92
5	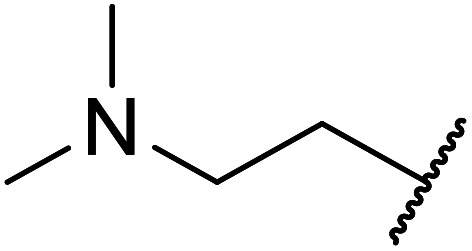	3	90
6	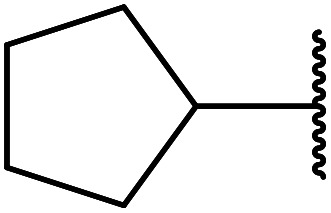	3	89
7	Cy	3	91
8	Menthyl	3	85
9	^*t*^Bu	4.5	65
10	Bn	3	90
11	Ph(CH_2_)_2_	3	87
12	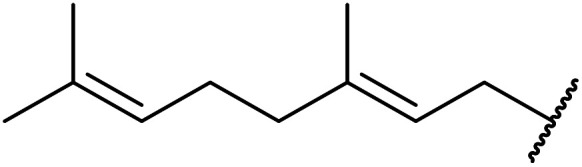	4	80
13	H_2_C=CHCH_2_	3	80
14	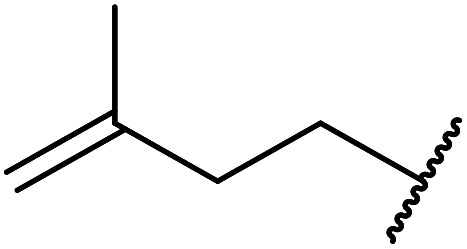	4	82

## 7. Clay catalysts

Natural clays are typically non-corrosive and low cost, making them popular catalysts in organic synthesis.^99^

Montmorillonite K-10 is a type of stratified silicate with acidic properties which can replace conventional acids.^100^ This mineral clay is made up of a three-layered structure: one octahedral aluminate layer sandwiched between two octahedral silicate layers. The interlayer cations are exchangeable and in their natural and ion-exchanged form both Brønsted and Lewis acidic catalytic sites are available. Montmorillonite-mediated transesterifications may be conducted in relatively benign hydrocarbon solvents and the catalyst is easily separated and reused (Table 42). Primary (entries 1, 2, 5, 6 and 8–14) and secondary (entries 3 and 7) alcohols were transesterified in good yields without issue. However, a significant reduction in yield with tertiary alcohols was recorded (entry 4).

**Table 42:** Montmorillonite K-10 catalysis^100^

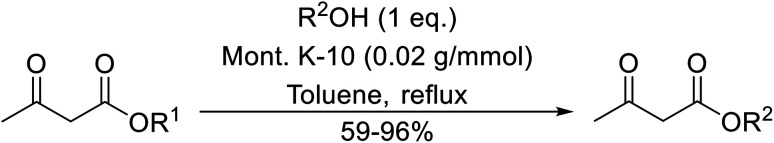				
Entry	R^1^	R^2^	Time (h)	Yield (%)
1	Me	^*n*^Bu	3	96
2	Me	^*n*^Pen	3	91
3	Me	^*sec*^Pen	2.5	87
4	Me	^*t*^Bu	4	59
5	Me	^*n*^Hep	3	92
6	Me	^*n*^Dec	3	93
7	Me	Cy	2.5	89
8	Me	H_2_C=CHCH_2_	2.5	92
9	Me	Bn	3.5	89
10	Me	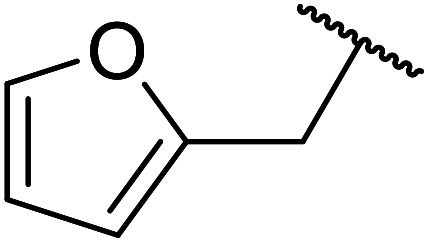	4	87
11	Et	^*n*^Bu	3	82
12	Et	^*n*^Dec	3	86
13	Et	Cy	2.5	84
14	Et	Bn	3.5	80

**Fig. 6:**
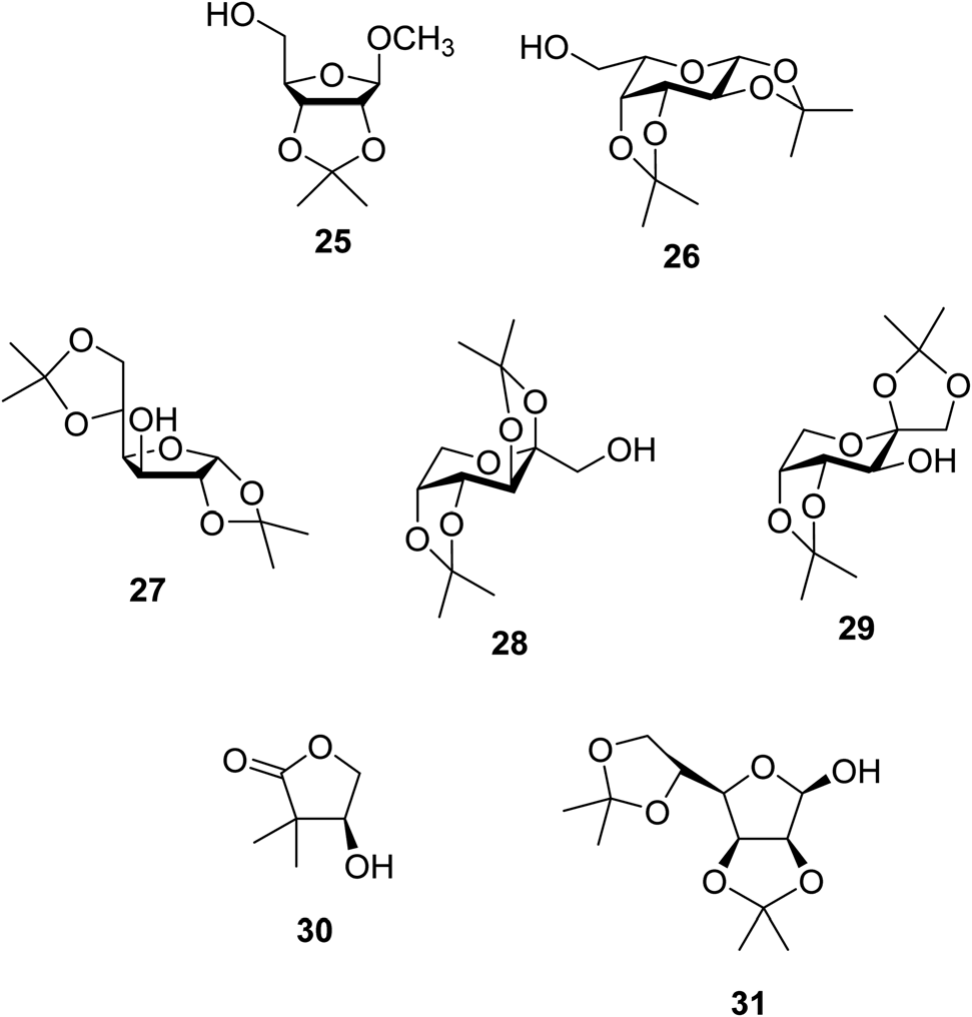
Carbohydrate-like alcohols for transesterification.

Further studies on montmorillonite K-10 by Perriera and co-workers led to the discovery that this catalyst degraded the carbohydrate-like alcohol 25, returning the transesterified product in only 34% yield. Screening of other clays, such as smectite, attapulgite and vermiculite (Fig. 6), identified several promising candidates (Table 43).^101^ The degree of degradation was greatly reduced with these other clays. In general, attapulgite and vermiculite performed better than smectite (entries 1–3). The transformation of anomeric alcohols was unsuccessful as the corresponding esters were too labile (entry 14). Variation of the ketone substituent (R^1^) of the β-keto esters had a negligible impact on yields. The catalysts could be recovered and reused three times with no appreciable loss in activity.

**Table 43:** Comparison of smectite, attapulgite or vermiculite clays^101^

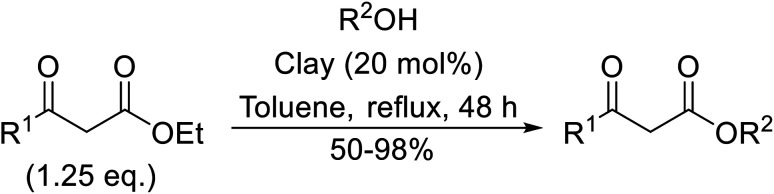					
Entry	R^1^	Alcohol	Smectite yield (%)	Attapulgite yield (%)	Vermiculite yield (%)
1	Me	25	73	93	93
2	Ph	25	72	75	76
3	Me	26	76	86	98
4	Ph	26	89	82	82
5	Me	27	50	50	51
6	Ph	27	78	80	82
7	Me	28	93	98	93
8	Ph	28	91	87	98
9	CF_3_	25	—	—	98
10	CF_3_	26	—	—	71
11	CF_3_	28	—	—	98
12	Me	29	—	—	84
13	Me	30	—	—	94
14	Me	31	—	—	0

**Fig. 7:**
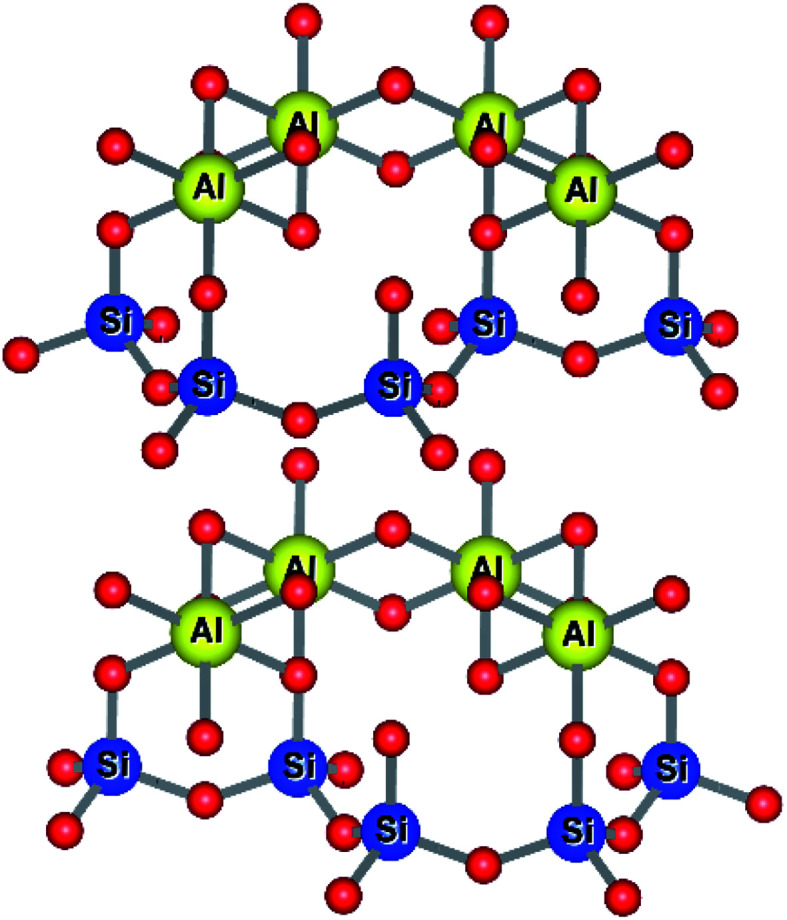
Structure of kaolinitic clays.^103,104^

Kaolinites are 1 : 1 clays consisting of one tetrahedral and one octahedral layer as shown in Fig. 7. The aluminium(iii) cations are bonded to an octahedral arrangement of oxygen anions. Likewise, a tetrahedral layer results from the repeating SiO_4_ silicate units.^102^

Ponde *et al.* have described the application of kaolinitic clay as a reusable, heterogenous catalyst for the selective transesterification of methyl β-keto esters.^105^ A variety of alcohols were transesterified using either montmorillonite K-10 clay or kaolinite clay with similar selectivity and reactivity (Table 44). Additionally, aniline was converted to the corresponding amide in 60% yield (entry 30). When the keto group at the β-position was protected, the transesterification was unsuccessful (entry 32). Kaolinitic clay also catalysed the transthioesterification (entries 17–23) and transamidation (entries 24 and 30) of β-keto esters. The authors further noted that kaolinitic clay facilitated the protection of carbonyls with ethane-1,2-diol, hydroxy thiol, dithiol or propane-1,3-dithiol.

**Table 44:** Mont. K-10 and kaolinitic clay catalysis^105^

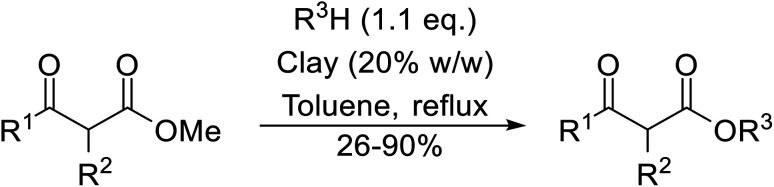						
Entry	R^1^	R^2^	R^3^	Time (h)	Kaolinite yield (%)	Mont. K-10 yield (%)
1	Me	H	BnO	3	85	86
2	Me	H	Ph(CH_2_)_2_O	3	87	80
3	Me	H	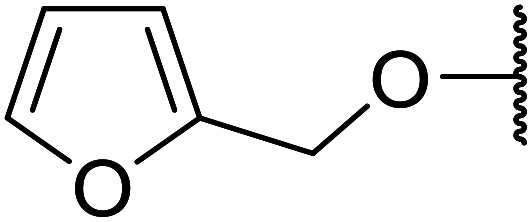	4	84	85
4	Me	H	PhCH=CHCH_2_O	6	80	72
5	Me	H	H_2_C=CHCH_2_O	6	75	—
6	Me	H	CyO	12	0	—
7	Me	H	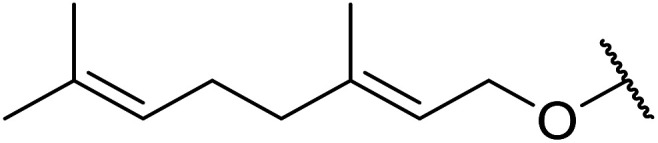	8	70	—
8	Me	H	CH_3_CH=CHCHO	8	80	75
9	Me	H	HC≡CCH_2_O	7	79	80
10	Me	H	^*n*^CH_3_(CH_2_)_11_O	10	75	70
11	Me	H	Menthol	12	n. r.	n. r.
12	Me	H	^*t*^BuO	12	n. r.	n. r.
13	Me	H	^*n*^CH_3_(CH_2_)_17_O	11	71	70
14	Me	H	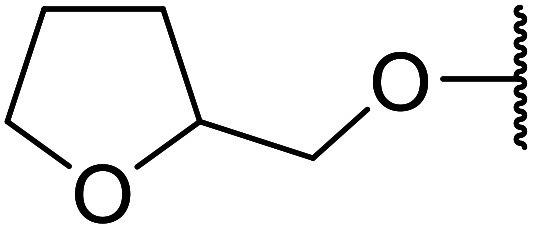	4	90	84
15	Me	H	Cl(CH_2_)_2_O	4	84	75
16	Ph	H	HC≡CCH_2_O	9	51	—
17	Me	H	PhS	8	70	—
18	Me	H	4-Cl–C_6_H_4_S	6	75	—
19	Me	H	2-EtO–C_6_H_4_S	8	64	—
20	Me	H	BnS	6	71	53
21	Me	H	4-MeO–C_6_H_4_S	8	69	—
22	Ph	H	BnS	12	42	26
23	Ph	H	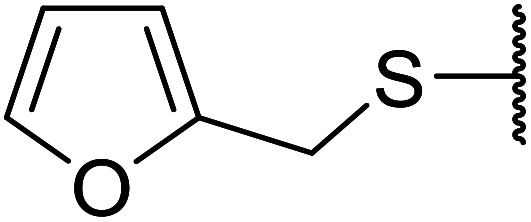	12	51	—
24	Me	H	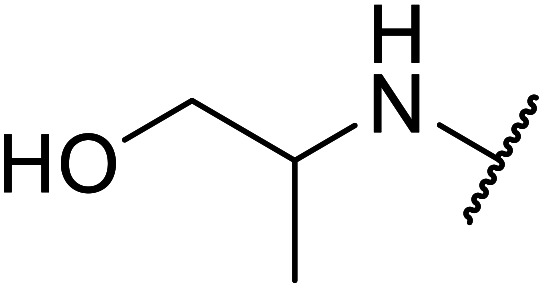	Not specified	61	58
25	Me	H	HS(CH_2_)_2_O	Not specified	49	48
26	Me	H	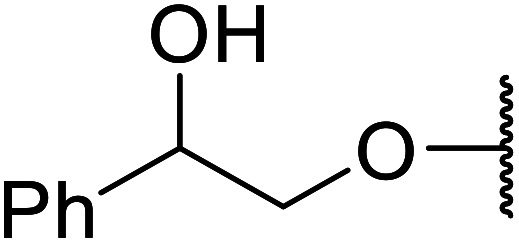	Not specified	48	—
27	Me	H	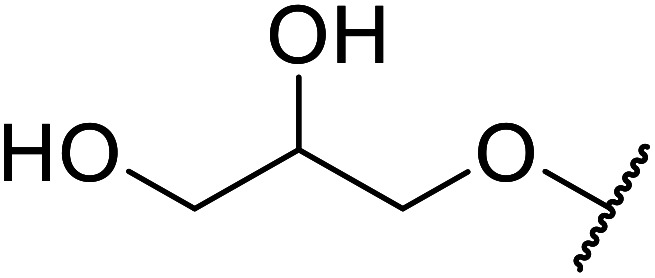	Not specified	55^a^	—
28	Me	H	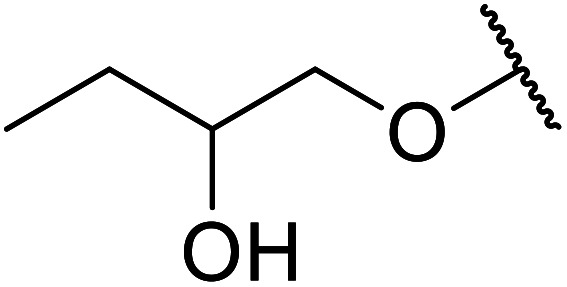	Not specified	60	54
29	Me	H	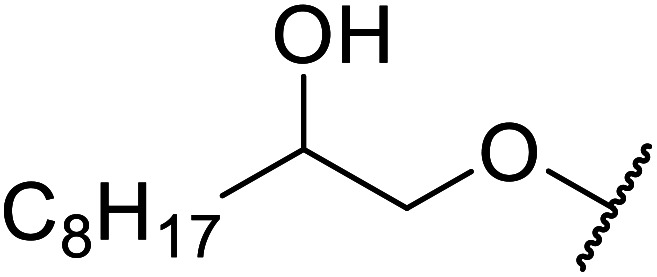	Not specified	55	53
30	Me	H	PhNH	Not specified	60	—
31	Me	Me	BnO	Not specified	35	—
32	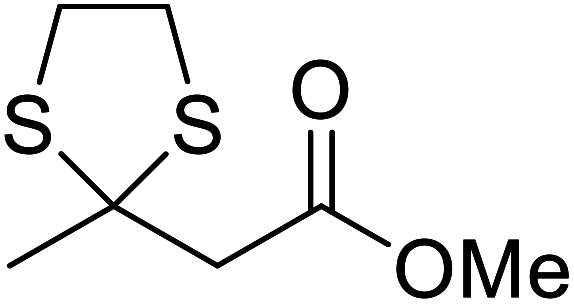		BnO	Not specified	n. r.	—

a Di-transesterification product isolated.

Envirocat EPZG® is a non-toxic, solid-supported reusable reagent with both Lewis acid and Lewis base properties. Its first reported use as a transesterification catalyst was by Bandgar and co-workers*.* (Table 45).^106^ Primary (entries 1–4, 8, 9, 12 and 14), secondary (entries 5, 6, 10, 11 and 13) and unsaturated alcohols (entries 8, 10, 11 and 14) furnished good yields, with a lower yield resulting from tertiary alcohols (entry 7). Lignan-type substrates were also compatible with this methodology (entry 14).

**Table 45:** Comparison of Envirocat EPZG® and natural clay (NC)^106^

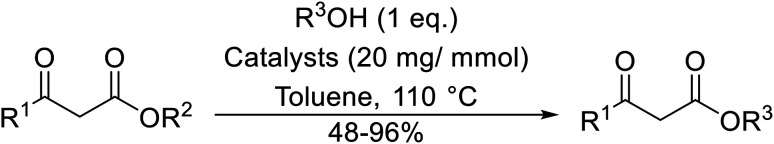						
Entry	R^1^	R^2^	R^3^	Time (h)	Catalyst	Yield (%)
1	Me	Me	Et	2	EPZG®	91
				2	NC	93
2	Me	Et	Me	2	EPZG®	91
				2	NC	92
3	Me	Et	^*n*^Bu	2	EPZG®	96
				2	NC	95
4	Me	Et	^*n*^Pen	2	EPZG®	94
				2	NC	89
5	Me	Me	Cy	3	EPZG®	88
				3.5	NC	89
6	Me	Et	Menthyl	2.5	EPZG®	88
				3	NC	89
7	Me	Me	^*t*^Bu	8	EPZG®	52
				8	NC	48
8	Me	Et	PhCH=CHCH_2_	2	EPZG®	63
				2	NC	60
9	Me	Et	4-MeO-C_6_H_4_CH_2_	2	EPZG®	82
				2	NC	84
10	Me	Et	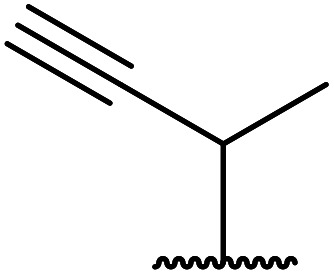	3	EPZG®	75
				3	NC	78
11	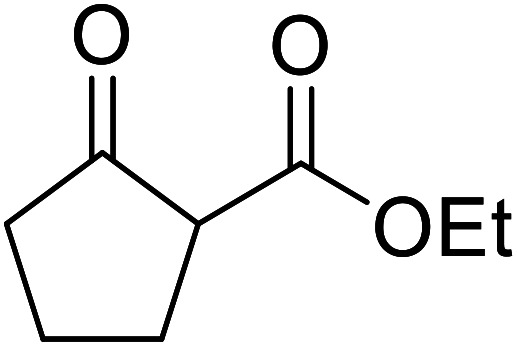		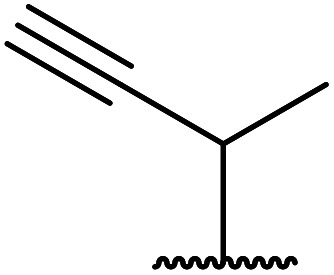	4	EPZG®	82
				4	NC	77
12	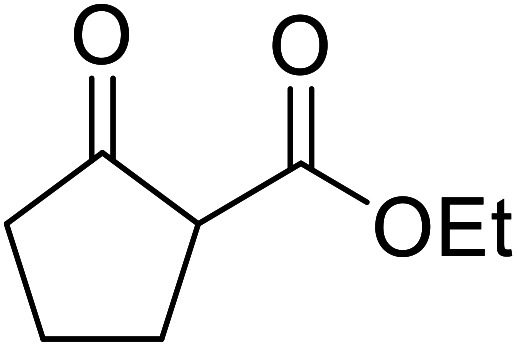		^*n*^Bu	2.5	EPZG®	76
				3	NC	72
13	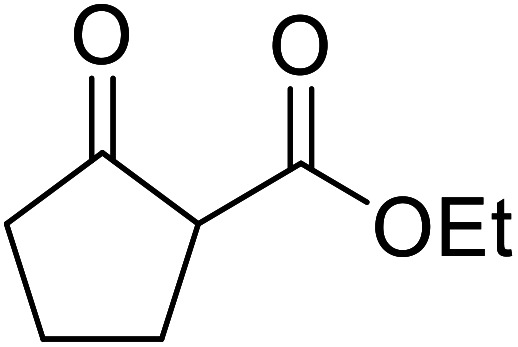		Menthyl	3.5	EPZG®	83
				3	NC	80
14	3,4,5-(MeO)_3_–C_6_H_2_	Me	PhCH=CHCH_2_	6	EPZG®	72
				6	NC	79

A heterogeneous nanocomposite catalytic system (HNCS) has been developed by Ratti and colleagues.^107^ This is a solid acid catalyst which was prepared by ion exchange of sulfonic acid functionalized ionic liquid (SAFIL) into montmorillonite (MMT) clay interlayers as an organic–inorganic HNCS (Table 46). HNCS accelerated the transesterification of β-keto esters with primary (entries 1–6 and 9–12), secondary (entries 7, 8, 13 and 14), cyclic (entries 13 and 14) and benzylic (entries 11 and 12) alcohols. SAFIL afforded equally good yields either in an ionic liquid, such as [TMBA] NTf_2_ (entry 1), or under solvent-free conditions (entry 2). The catalytic activity of the HNCS was measured in the absence and presence of [TMBA] NTf_2_ solvent (entries 3 and 4), and a 0% yield was recorded when no solvent was present. Recycling experiments confirmed that both the catalyst and ionic liquid may be reused up to seven times.

**Table 46:** [TMBA] NTf_2_ catalysis^107^

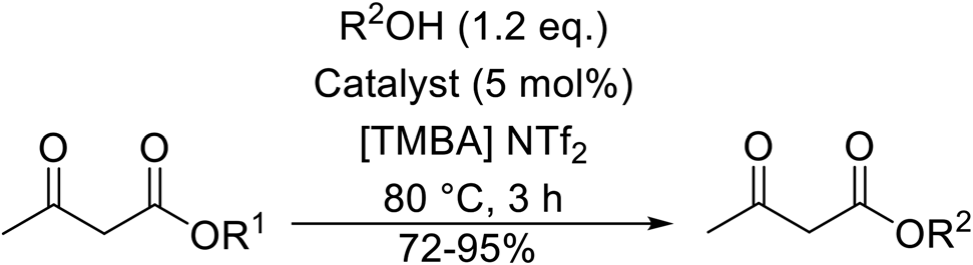				
Entry	Catalyst	R^1^	R^2^	Yield (%)
1	SAFIL	Et	^*n*^Oct	92
2	SAFIL	Et	^*n*^Oct	90^a^
3	HNCS	Et	^*n*^Oct	95
4	HNCS	Et	^*n*^Oct	0^a^
5	SAFIL	Me	^*n*^Bu	90
6	HNCS	Me	^*n*^Bu	85
7	SAFIL	Me	^i^Bu	80
8	HNCS	Me	^i^Bu	90
9	SAFIL	Me	H_2_C=CHCH_2_	75
10	HNCS	Me	H_2_C=CHCH_2_	88
11	SAFIL	Me	Bn	72
12	HNCS	Me	Bn	80
13	SAFIL	Me	Cy	75
14	HNCS	Me	Cy	80

a No [TMBA] NTf_2_ solvent used.

Vázquez and co-workers compared several different hybrid silica sol–gels as potential catalysts (Table 47).^108,109^ SAF (modified silica with AP) and SHF (modified silica with HS) type sol–gels were prepared using 3-aminopropyltriethoxysilane (AP) and 1,1,1,3,3,3-hexamethyldisilazane (HS) respectively. Functionalised silica sol–gels with different modifier loadings (SAF, SAFI and SAFII) and combinations of modifiers (SAHMI and SAHMII) were also examined (Table 47). The use of pure silica (entry 1), SAFI (entry 4) or SAFII (entry 5) to catalyse the reaction of ethyl acetoacetate with 3-phenylpropanol resulted in unwanted ether formation due to self-coupling of the alcohol. The highest yields and shortest reaction times were associated with the SHF catalyst (entry 2). Similar activity was noted for SAHMII (entry 7), but a surprisingly poor yield of 49% was recorded with the SAHMI catalyst (entry 6). The nature of the surface groups of the SAHMI and SAHMII catalysts afforded better hexamethyldisilazane dispersion and improved accessibility to the active sites. For each alcohol tested, comparable yields were obtained with either ethyl acetoacetate (entries 1–13) or methyl acetoacetate (entries 15–21).

**Table 47:** Silica sol–gel catalysis^108,109^

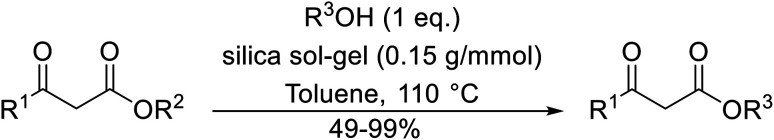							
Entry	R^1^	R^2^	R^3^	Catalyst	Modifiers (mL)	Time (h)	Conversion (%)
1	Me	Et	Ph(CH_2_)_3_	Silica	—	10	99^a^
2	Me	Et	Ph(CH_2_)_3_	SHF	HS (3)	4	98
3	Me	Et	Ph(CH_2_)_3_	SAF	AP (3)	7	92
4	Me	Et	Ph(CH_2_)_3_	SAFI	AP (0.2)	16	99^a^
5	Me	Et	Ph(CH_2_)_3_	SAFII	AP (0.6)	11	99^a^
6	Me	Et	Ph(CH_2_)_3_	SAHMI	AP (3)/HS (1.5)	7.5	49
7	Me	Et	Ph(CH_2_)_3_	SAFMII	AP (3)/HS (4.5)	4.5	90
8	Me	Et	(−)-Menthyl	SHF	HS (3)	4	94
9	Me	Et	(−)-Menthyl	SAF	AP (3)	8	77
10	Me	Et	Bn	SHF	HS (3)	4	92
11	Me	Et	4-MeO-C_6_H_4_CH_2_	SAF	AP (3)	7	92^b^
12	Me	Et	Geraniol	SAF	AP (3)	6	95^b^
13	Me	Et	Linalool	SAF	AP (3)	7	52^b^
14	Ph	Et	Ph(CH_2_)_3_	SAF	AP (3)	8	80^b^
15	Me	Me	Ph(CH_2_)_3_	SHF	HS (3)	4	97
16	Me	Me	Ph(CH_2_)_3_	SAF	AP (3)	8	88
17	Me	Me	Cholesterol	SAF	AP (3)	11	98^b^
18	Me	Me	(−)-Menthyl	SAF	AP (3)	10	74^b^
19	Me	Me	PhOEt	SAF	AP (3)	10	98^b^
20	Me	Me	Bn	SAF	AP (3)	6	77^b^
21	Me	Me	EtSEt	SAF	AP (3)	14	72^b^

a Secondary product detected (bis-3-phenylpropil-ether).

b Solvent-free conditions.

## 8. Mesoporous and microporous material-based catalysts

Mesoporous materials contain pores that are between 2 nm and 50 nm in diameter, while microporous materials have a pore size of less than 2 nm in diameter.^110^ These materials possess a large surface area, making them ideal for the purpose of catalysis.

**Scheme 8:**
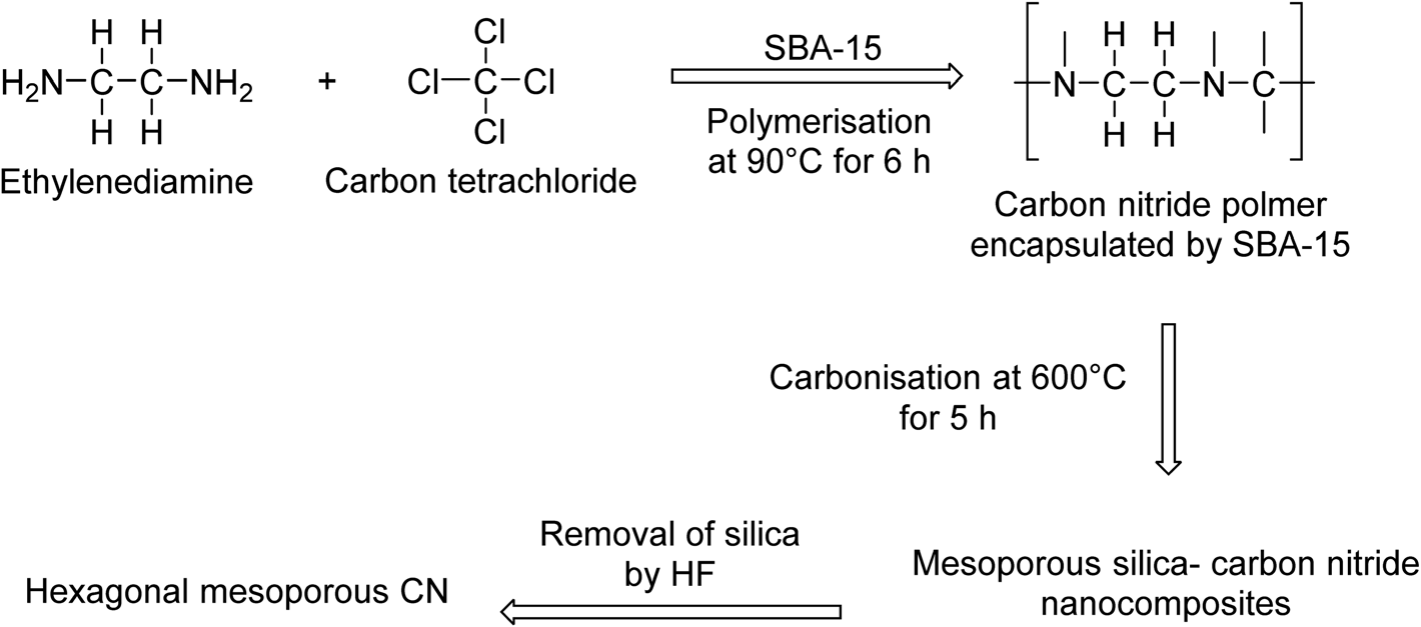
Preparation of mesoporous CN using SBA-15.^112^

Catalysts are typically prepared *via* a polymerisation reaction between ethylenediamine (EDA) and carbon tetrachloride (Scheme 8). Mesoporous carbon nitride (MCN) is a carbon-based material that boasts a well-ordered and tunable porous structure, with an extremely high nitrogen content.^111^ Varying the ratio of EDA to carbon tetrachloride allows for the pore diameter, specific surface area and the nitrogen content to be altered.

Vinu and co-workers have found that MCN-1, derived from SBA-15, is highly active in the solvent-free transesterification of cyclic, aromatic, long and short chain primary alcohols to afford the corresponding β-keto esters in yields up to 69% in 6 hours.^113^ MCN-1 performed better with aliphatic alcohols (*e.g. n*-butanol and *n*-octanol) than cyclic alcohols (*e.g.* cyclohexanol and furfuryl alcohol) or aromatic alcohols (*e.g.* benzyl alcohol). A 2% catalyst loading was found to be optimal, with higher loadings leading to lower yields, likely due to a poisoning effect. By comparing the activity of MCN-1 with mesocarbon (CMK-3), it was concluded that the basic amine groups along the walls of the material offer strong Lewis basic sites. Recycling studies were also performed, and MCN-1 could be reused at least three times without any significant loss of catalytic activity.

As an alternative to mesoporous carbon nitride, nitrogen-containing ordered mesoporous carbon (NOMC) material offers many advantages in both preparation and application.^114^ The nitrogen percentage can be modified with average pore sizes of 3.4–3.7 nm. NOMC-550 displays superior catalytic activity compared to other NOMC materials due to its optimal surface area and nitrogen composition. Xu *et al.* employed NOMC-550 for the transesterification of β-keto esters (Table 48).^115^ A reaction time of 6 hours and temperature of 100 °C are optimum for this catalyst. After five consecutive reactions, there was little fall off in catalytic activity. Aliphatic alcohol (entries 1 and 2), cyclic alcohols (entry 3) and aromatic alcohols (entries 4–6) afforded moderate conversions.

**Table 48:** NOMC-550 catalysis^115^

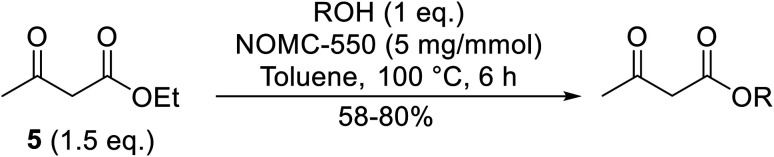		
Entry	R	Conversion (%)
1	^*n*^Bu	80
2	^*n*^Oct	61
3	Cy	75
4	Bn	67
5	PhCH=CHCH_2_	61
6	Ph(CH_2_)_2_	58

**Fig. 8:**
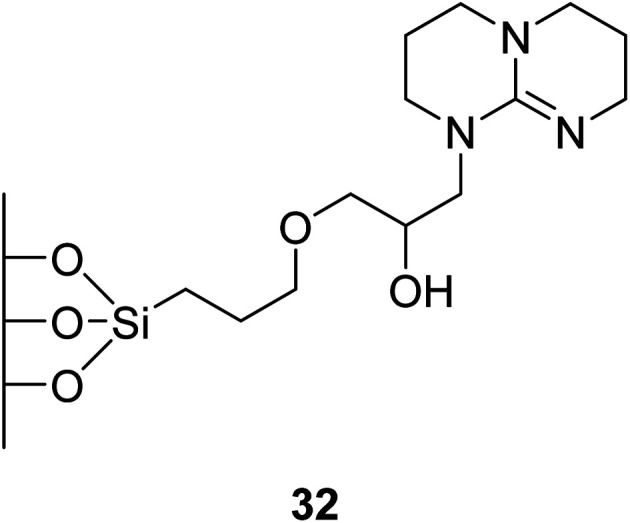
Structure of TBD-MCM.

Kantam and Sreekanth have investigated a heterogeneous guanidine-derived catalyst, 1,5,7-triazabicyclo[4.4.0]dec-5-ene (TBD), anchored on MCM-41 mesoporous material (TBD-MCM) (Fig. 8).^116^*n*-Butyl esters were found to react faster and in higher yields with ethyl acetoacetate (Table 49, entry 3) than with methyl acetoacetate (entry 2). When an aminoalcohol was introduced, the reaction occurred exclusively at the hydroxyl and no transamidation was reported (entry 9). By virtue of the simple alkyl-spacer which anchors guanidine, the robust TBD-MCM catalyst does not leach guanidine. The mechanical stability of the inorganic support materials also ensures easy separation of the solid catalyst.

**Table 49:** TBD-MCM catalysis^116^

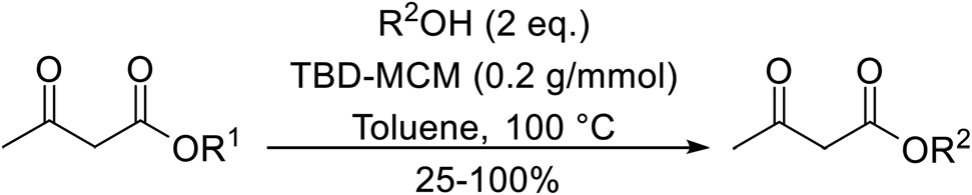				
Entry	R^1^	R^2^	Time (h)	Yield^a^ (%)
1	Me	Bn	24	60
2	Me	^*n*^Bu	24	80 (75^b^)
3	Et	^*n*^Bu	12	100
4	Me	^*n*^Hex	12	80
5	Me	^*n*^Oct	6	52
6	Me	^*n*^Oct	24	25^c^
7	Me	Geraniol	24	83
8	Et	Me	12	45
9	Me	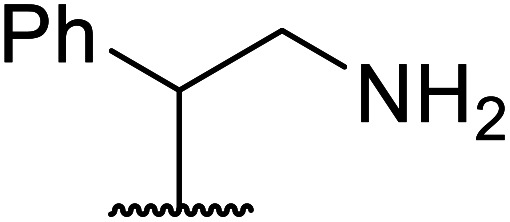	12	60
10	Me	HC≡CCH_2_	24	45

a Yield based on ^1^H-NMR, based on β-keto ester.

b 5th recycle.

c Using homogeneous catalyst.

Xu *et al.* have probed the utility of mesoporous CN-FDU-12 in catalysing transesterifications (Table 50).^117^ The surface area and pore volume in CN-FDU-12 are 702 m^2^ g^−1^ and 1.4 cm^3^ g^−1^ respectively. Benzyl alcohol (entry 5) and the heterocyclic furfuryl alcohol (entry 4) afforded comparable conversions to aliphatic alcohols (entries 1–3) demonstrating the substrate scope of this system. Cyclic alcohols proved similarly compatible (entry 3). The Lewis base type tertiary N species are speculated to be the active sites in the catalyst.

**Table 50:** CN-FDU-12 catalysis^117^

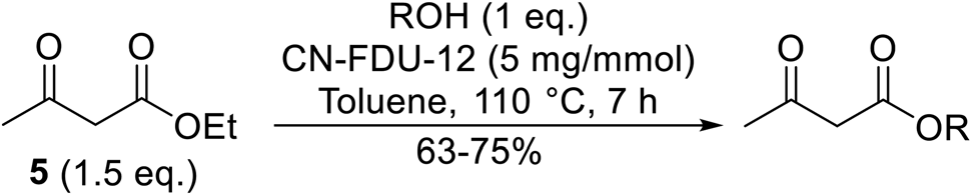		
Entry	R	Conversion^a^ (%)
1	^*n*^Bu	74
2	^*t*^Bu	64
3	Cy	75
4	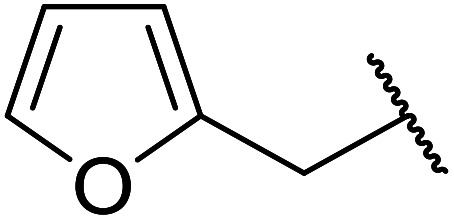	63
5	Bn	70

a Conversion determined using GC analysis.

Wang and co-workers discovered that mpg-C_3_N_4_ is an effective catalyst for both Knoevenagel condensations and transesterifications (Table 51).^118^ The overall performance could be significantly improved if the catalyst was previously treated with base, in the order ^*t*^BuOK > KOH > K_2_CO_3_. Long-chain primary alcohols (entry 4) and hindered cyclic alcohols (entry 5) could both be converted into the corresponding esters, albeit in much higher yields with the former. The heterogeneous catalyst was highly stable and could be reused several times.

**Table 51:** Mpg–C_3_N_4_–^*t*^Bu catalysis^118^

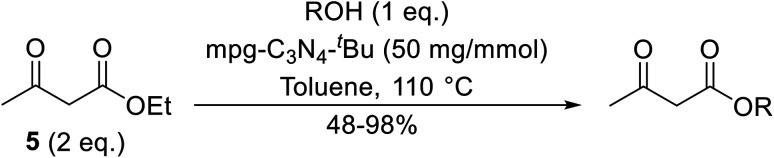			
Entry	R	Time (h)	Conversion^a^ (%)
1	Bn	4.5	88
2	4-MeO-C_6_H_4_CH_2_	6	61
3	PhCH_2_=CH_2_	5	90
4	^*n*^Hex	5	98
5	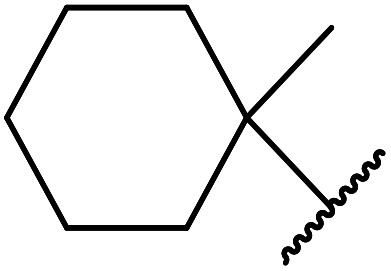	5.5	48

a Conversion determined using GC analysis.

Sasidharan and co-workers studied H-β-zeolites as catalysts for the transesterification of several β-keto esters (Table 52).^119–121^ The higher acid strength of H-β-zeolites, coupled with large pore openings and void spaces, may account for its superior activity. The catalyst was reused three times without loss of activity. Open chain (entries 1–5 and 7–18) and cyclic aliphatic β-keto esters (entries 6 and 19) afforded higher yields compared to aryl β-keto esters (entries 7–12), where 15–20% of starting material was recovered. The bulky nature of these substrates may limit diffusion into the pores of the zeolite catalyst. The catalyst is selective for β-keto esters with other esters, including α-keto esters (entry 20), α-halogenated esters (entry 22), unsaturated esters (entry 23) as well as simple esters (entry 21), remaining unreacted. Solvent-free, microwave conditions were examined and furnished high yields of the corresponding products after just 10–15 minutes. This compares favourably with the 8–10 hours required with conventional heating (entries 24–31).

**Table 52:** H-β-zeolite catalysis^119–121^

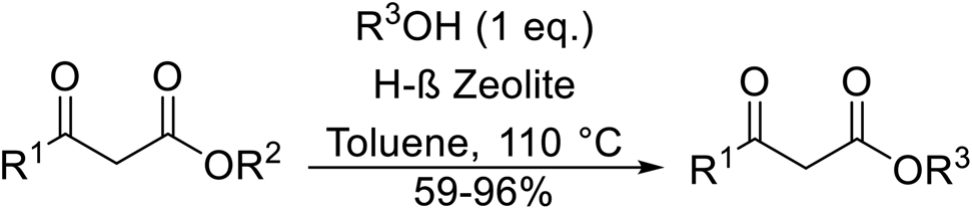								
Entry	R^1^	R^2^	R^3^	Catalyst loading	Conventional		Microwave	
					Time (h)	Yield (%)	Time (min)	Yield^a^ (%)
1	Me	Et	Ph(CH_2_)_2_	10 mol%	8	96	—	—
2	Me	Et	^*n*^C_12_H_25_	10 mol%	8	86	—	—
3	Me	Et	Bn	10 mol%	8	81	—	—
4	Me	Et	Menthyl	10 mol%	8	95	—	—
5	Me	Et	PhCH=CHCH_2_	10 mol%	8	95	—	—
6	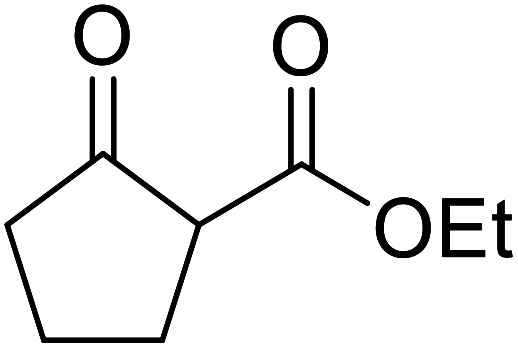		PhCHCHCH_2_	10 mol%	8	84	—	—
7	Ph	Me	PhCH=CHCH_2_	10 mol%	8	66	—	—
8	Ph	Me	Ph(CH_2_)_3_	10 mol%	8	64	—	—
9	Ph	Me	^*n*^CH_3_(CH_2_)_11_	10 mol%	8	71	—	—
10	Ph	Me	Menthyl	10 mol%	8	69	—	—
11	3,4,5-(MeO)_3_–C_6_H_2_	Me	PhCH=CHCH_2_	10 mol%	8	62	—	—
12	3,4,5-(MeO)_3_–C_6_H_2_	Me	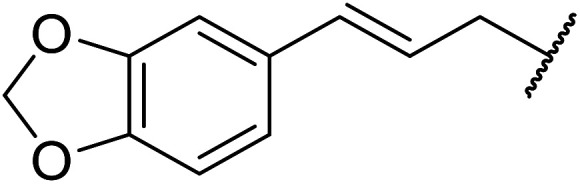	10 mol%	8	59	—	—
13	Me	Et	^*n*^Hex	20 wt%	10	92	—	—
14	Me	Et	^*n*^Bu	20 wt%	10	87	—	—
15	Me	Et	Me	20 wt%	10	85	—	—
16	Me	Et	^*t*^Bu	20 wt%	10	66	—	—
17	Me	Et	Cy	20 wt%	10	87	—	—
18	Me	Me	^*n*^Hex	20 wt%	10	85	—	—
19	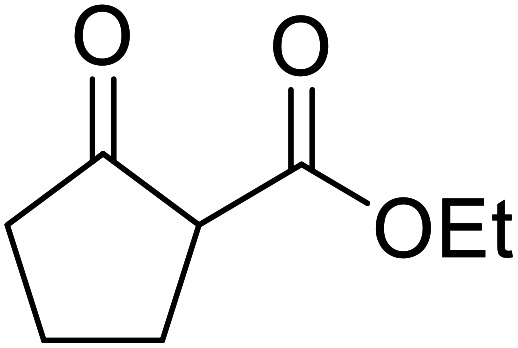		^*n*^Hex	20 wt%	10	79	—	—
20	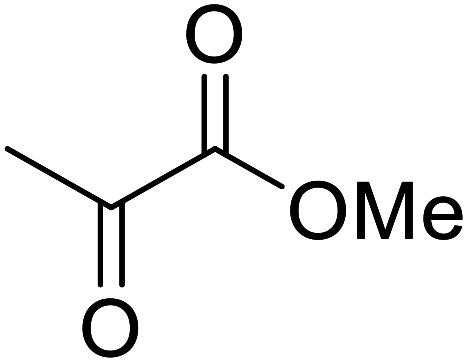		^*n*^Hex	20 wt%	10	—	—	—
21	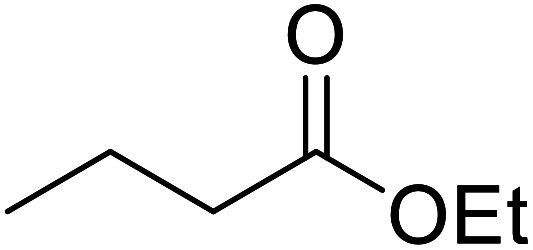		^*n*^Hex	20 wt%	10	—	—	—
22	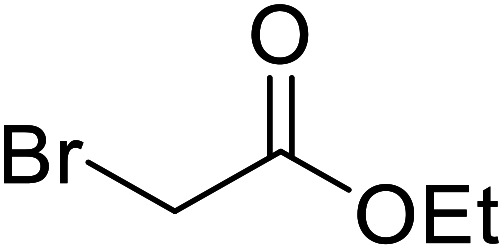		^*n*^Hex	20 wt%	10	—	—	—
23	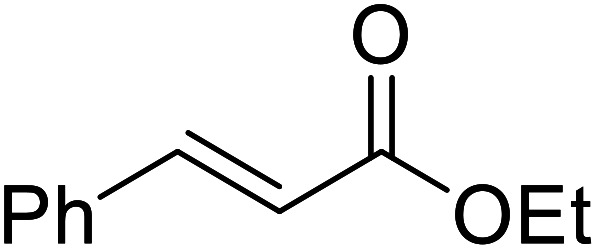		^*n*^Hex	20 wt%	10	—	—	—
24	Me	Me	Ph(CH_2_)_3_	10 wt%	—	—	10	95
25	Ph	Et	PhCH=CHCH_2_	10 wt%	8	66	13	78
26	Ph	Et	Ph(CH_2_)_2_	10 wt%	8	64	15	80
27	Ph	Et	EtO(CH_2_)_2_	10 wt%	—	—	10	72
28	Ph	Et	Cl(CH_2_)_2_	10 wt%	—	—	11	70
29	Ph	Et	Br(CH_2_)_2_	10 wt%	—	—	11	73
30	Ph	Et	Ph(CH_2_)_2_	10 wt%	—	—	10	80
31	3,4,5-(MeO)_3_–C_6_H_2_	Et	Ph(CH_2_)_3_	10 wt%	—	—	15	69

a 1 : 1 amount of β-keto ester and alcohol were mixed and microwaved as a neat mixture with 10% (w/w of β-keto ester) of the catalyst.

The acidic form of the zeolite ferrierite, namely H-ferrierite (H-FER), is an exceptionally stable and shape selective catalyst for the isomerisation of butane to isobutene.^122–124^ Chavan *et al.* have reported the transesterification of β-keto esters catalysed by H-FER under solvent-free conditions (Table 53).^125^ In most cases, 1.2 equivalents of alcohol was sufficient, but for volatile alcohols (entries 3, 6, 7, 11 and 12) a higher loading of 2 equivalents was required. Both primary (entries 3, 5–10 and 12–16) and secondary (entries 1, 2, 4 and 11) alcohols were readily esterified. However, *tert*-butanol did not furnish the corresponding *tert*-butyl ester. Substitution at the α-position hindered the transesterification of ethyl 2-methyl-3-oxobutanoate with benzyl alcohol (entry 15). Ethyl 2,2-dimethyl-3-oxobutanoate failed to react, due to the absence of an enolisable α-proton (entry 16). This zeolite may be reused several times without any loss of activity.

**Table 53:** H-FER catalysis^125^

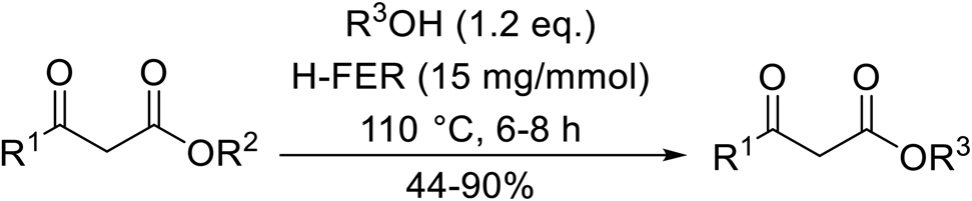					
Entry	R^1^	R^2^	R^3^	Alcohol (eq.)	Yield (%)
1	Me	Me	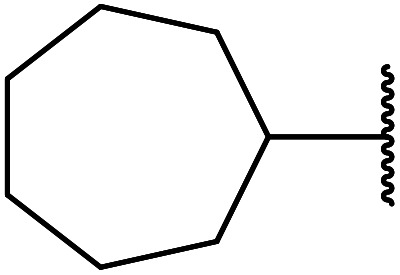	1.2	72
2	Me	Et	(−)-Menthyl	1.2	90
3	Me	Me	HC≡CCH_2_	2	61
4	Me	Me	Cy	1.2	85
5	Me	Me	Bn	1.2	90
6	Me	Me	H_2_C=CHCH_2_	2	65
7	Me	Me	^i^Bu	2	84
8	Me	Me	^*n*^Dec	1.2	70
9	Me	Me	^*n*^Oct	1.2	71
10	Me	Me	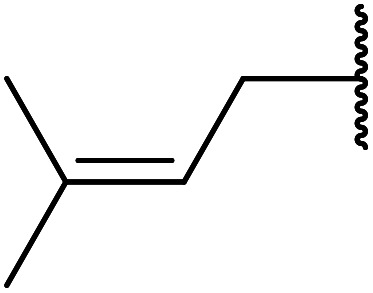	1.2	80
11	Me	Me	^i^Pr	2	59
12	Me	Me	^*n*^Pr	2	65
13	Ph	Et	4-MeO-C_6_H_4_CH_2_	1.2	74
14	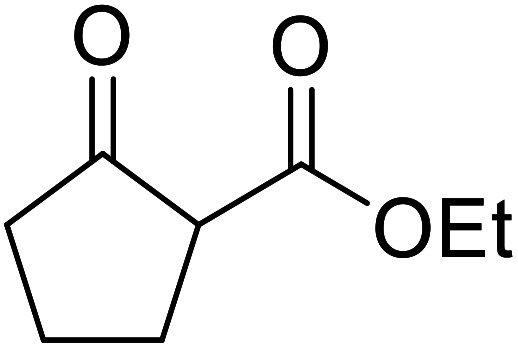		^*n*^Hex	1.2	72
15	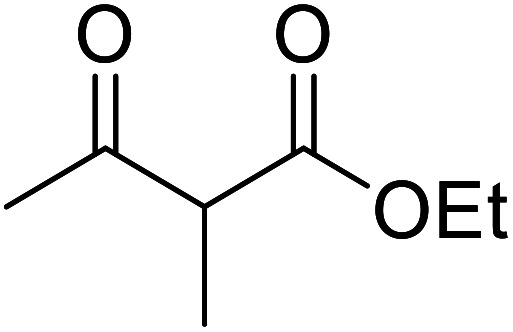		Bn	1.2	44
16	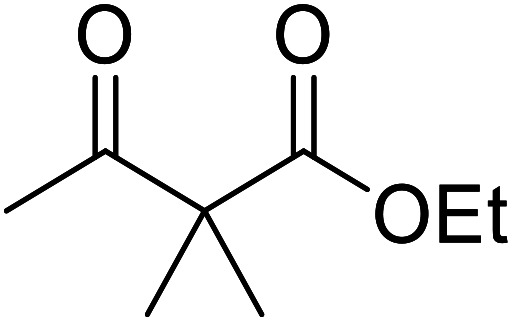		Bn	1.2	n. r.

## 9. Catalyst-free methodologies

Depending on the stability of the reactants or products, the presence of a catalyst may not always be well tolerated. This has driven the development of alternative approaches where transesterification is promoted by other means, including distillation, molecular sieves or microwave irradiation.

A catalyst-free transesterification of methyl, ethyl and *tert-*butyl β-keto esters with primary and secondary propargyl alcohols using a distillation apparatus has been described by Mottet and colleagues (Table 54).^126^ This process is not trivial, as traditional acid- and base-catalysed transesterifications with propargyl alcohols produce low yields due to Carroll rearrangement. The transformation of C-2 substituted β-keto esters (entries 4–12) suggests that a ketene intermediate is likely involved. The absence of catalyst does lead to very slow reactions, with typical reaction times extending from 1 day (entries 1–3) to almost 2 weeks (entries 5 and 7).

**Table 54:** Catalyst-free conditions^126^

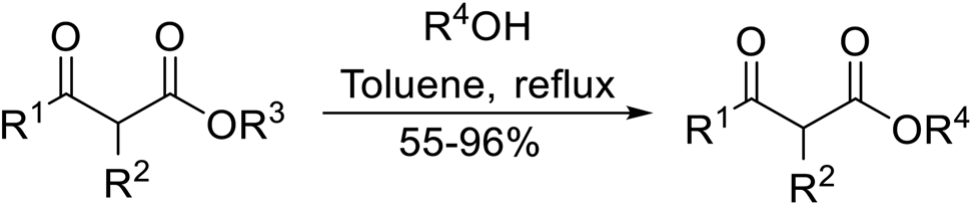							
Entry	R^1^	R^2^	R^3^	R^4^	Alcohol (eq.)	Time (days)	Yield
1	^*n*^Pr	H	Et	HC≡CCH_2_	5	1	91
2	^*n*^Pr	H	Et	CH_3_C≡CCH_2_	1.5	1	94
3	Ph	H	Et	HC≡CCH_2_	5	1	96
4	Me	CHCH=CH_2_	Me	HC≡CCH_2_	5	10	70
5	Me	PhCH_2_	Me	HC≡CCH_2_	5	12	69
6	Me	PhCH_2_	Me	HC≡CCH_2_	5	6	55
7	Me	^i^Pr	^*t*^Bu	HC≡CCH_2_	5	12	79
8	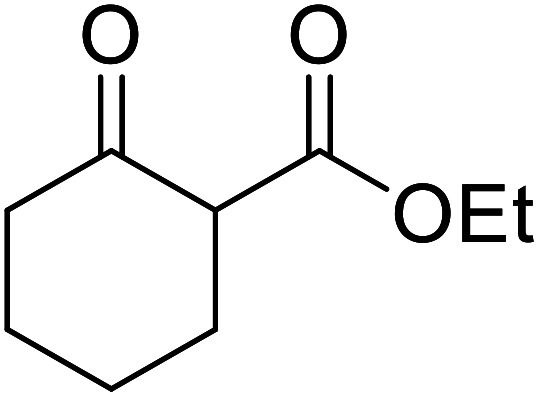			HCCCH_2_	5	4	59
9	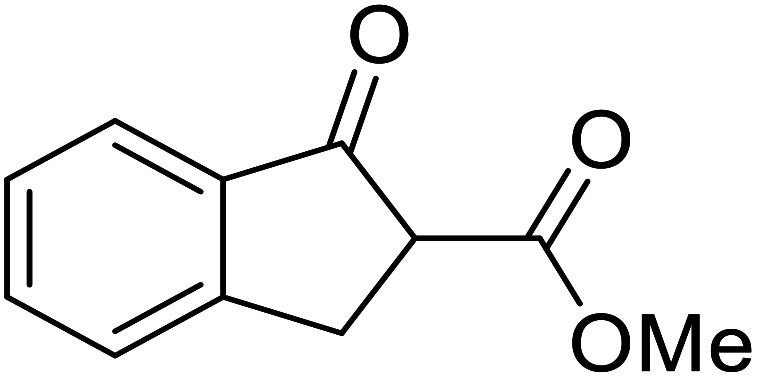			HCCCH_2_	5	0.8	86
10	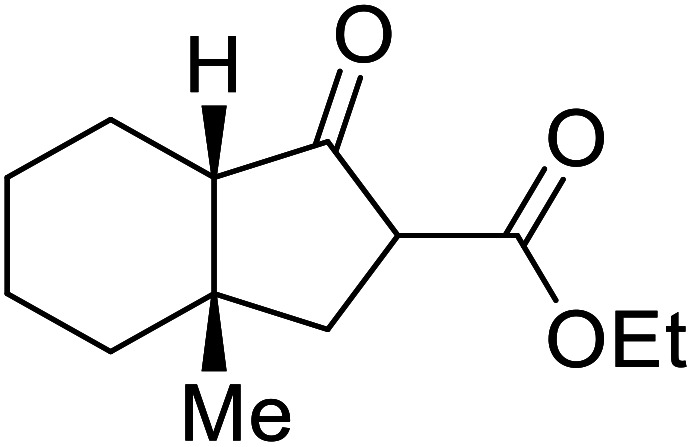			HCCCH_2_	5	1	90
11	Me	CH_2_Ph	Me	HC≡CCH(Ph)	1.2	10	85
12	Me	CH_2_Ph	Me	HC≡CCH(^*n*^Pe)	1.2	10	83

**Scheme 9:**
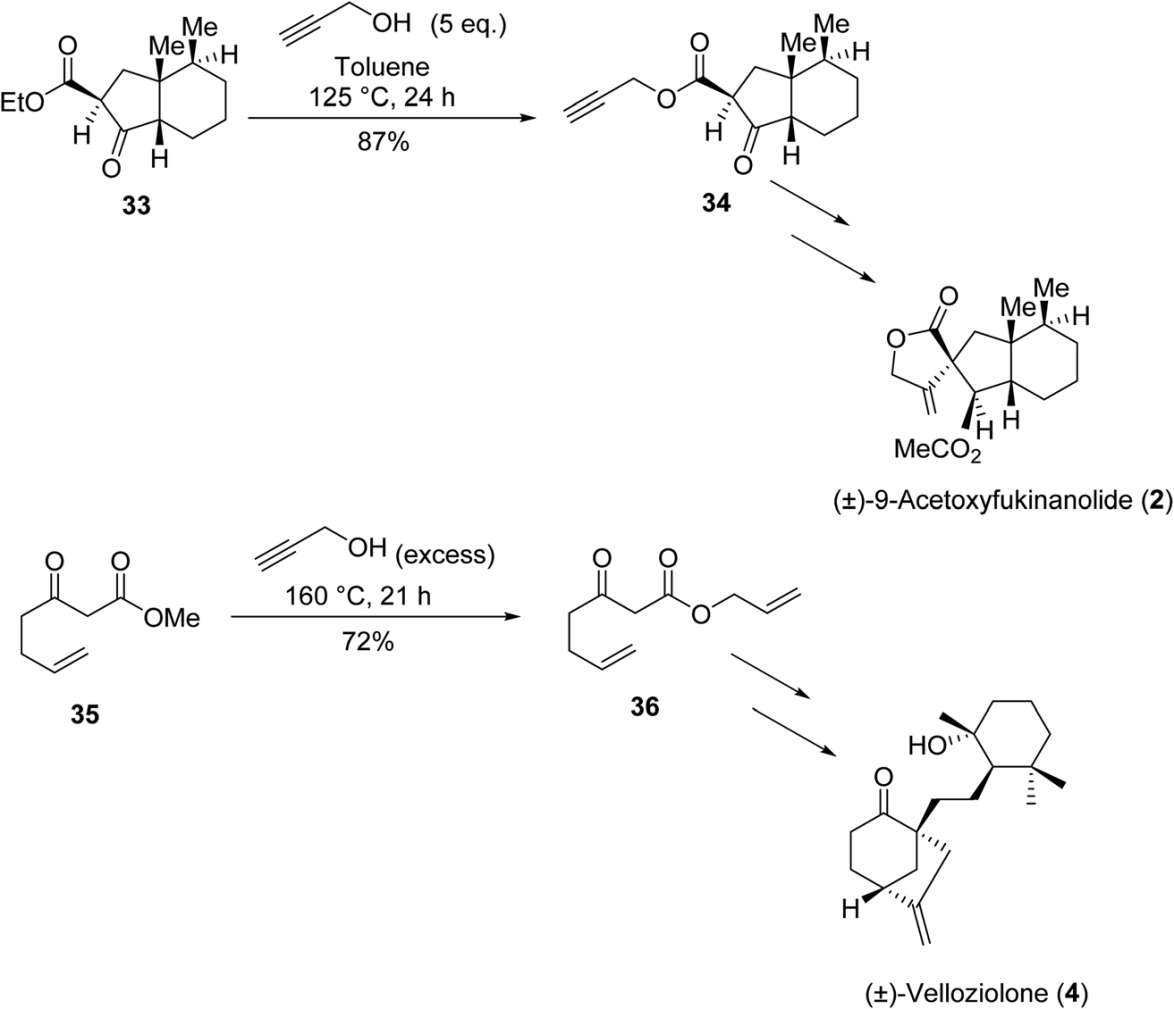
Synthesis of (±)-9-acetoxyfukinanolide and (±)-velloziolone.

Successful catalyst-free transesterifications have been reported on several occasions in the literature, such as the synthesis of (±)-9-acetoxyfukinanolide and (±)-velloziolone (Scheme 9).^4,5^

Molecular sieves with pore sizes of 4 Å or greater can absorb low molecular weight alcohols such as ethanol and methanol. The removal of these alcohol by-products drives transesterification processes forward (Table 55). Primary (entries 1 and 2), secondary (entries 3–5) and tertiary (entries 6 and 7) alcohols were transesterified in high yields on addition of 4 Å molecular sieves, with reaction times increasing as the steric crowding around the hydroxyl group increased.^127^ Tertiary alcohols proved significantly less reactive and the yield of 1-adamantanyl acetoacetate was poor (entry 6).

**Table 55:** Impact of 4 Å molecular sieves^127^

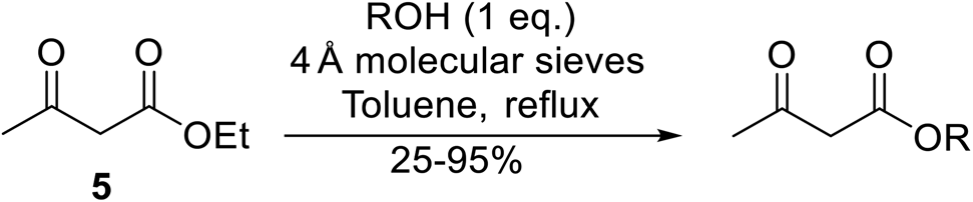			
Entry	R	Time (h)	Yield (%)
1	^*n*^Hex	4	95
2	^*n*^CH_3_(CH_2_)_11_	5	93
3	Cy	9.5	87
4	Menthyl	12	92
5	Borneol	16	78
6	1-Adamantanol	24	25
7	2-Methyl-2-butanol	9	90

Using a domestic microwave oven, Gianotti *et al.* successfully transesterified ethyl β-keto esters with a variety of alcohols (Table 56).^128^ The presence of a TBDMS protecting group did not interfere with the expected outcome (entry 1–5). Only enolisable β-keto esters were found to react, suggesting that the reaction proceeds *via* an enol intermediate in this instance. Both aromatic (entries 2, 4, 7 and 9) and aliphatic alcohols (entries 1, 3, 5, 6, 8 and 10) furnished the corresponding esters in good to excellent yields. The only exception was the transesterification of ethyl acetoacetate with diphenylmethanol, which resulted in a 62% yield (entry 9). Employing *tert*-butyldimethylsilanol afforded a similar yield of 60% (entry 11).

**Table 56:** Microwave heating^128^

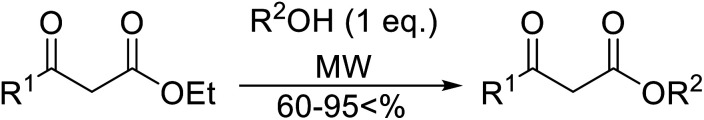					
Entry	R^1^	R^2^	Power (W)	Time (min)	Yield (%)
1	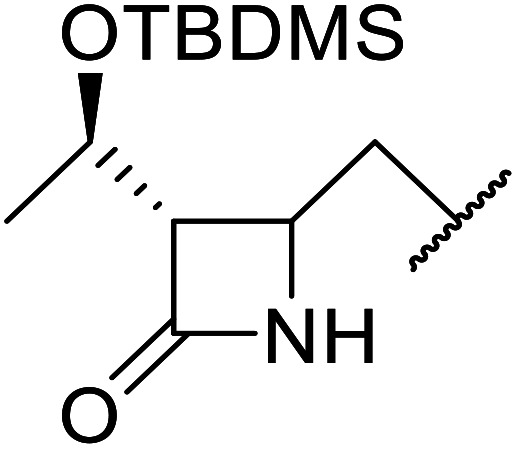	^*n*^CH_3_(CH_2_)_11_	650	40	>95
2	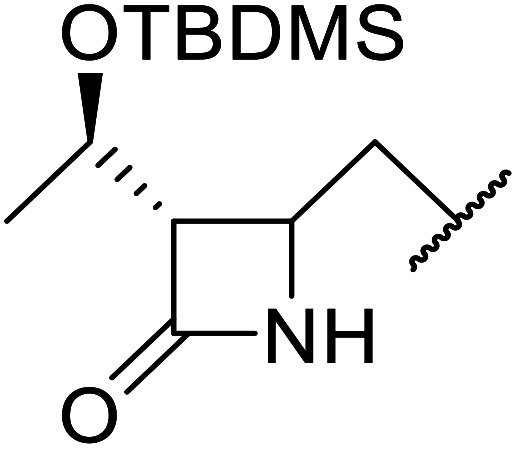	4-NO_2_–C_6_H_4_CH_2_	650	30	89
3	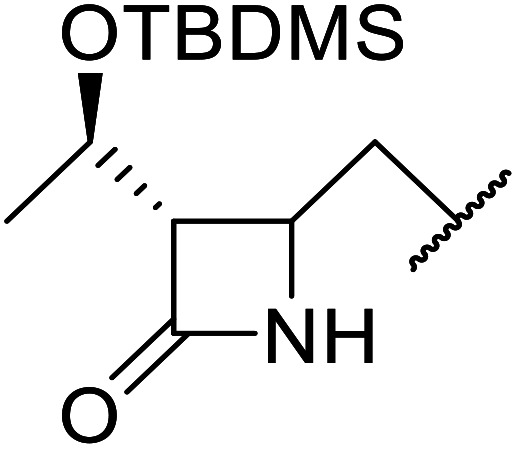	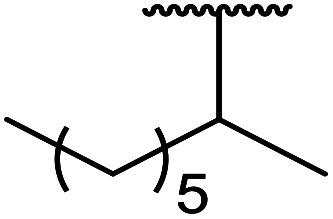	650	30	92
4	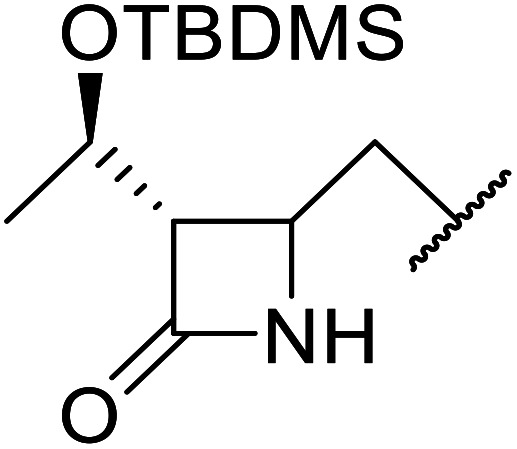	(Ph)_2_CH	650	30	81
5	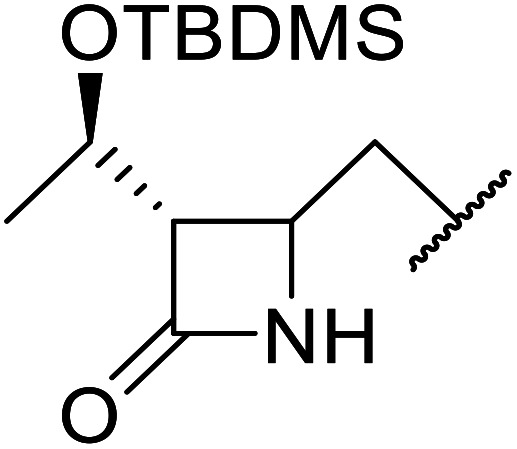	(−)-Menthyl	650	30	93
6	Me	^*n*^CH_3_(CH_2_)_11_	650	30	>95
7	Me	4-NO_2_–C_6_H_4_CH_2_	750	30	82
8	Me	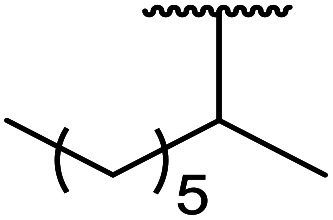	650	30	>95
9	Me	(Ph)_2_CH	750	30	62
10	Me	(−)-Menthyl	650	25	92
11	Me	TBDMSi-	650	20	60

**Scheme 10:**
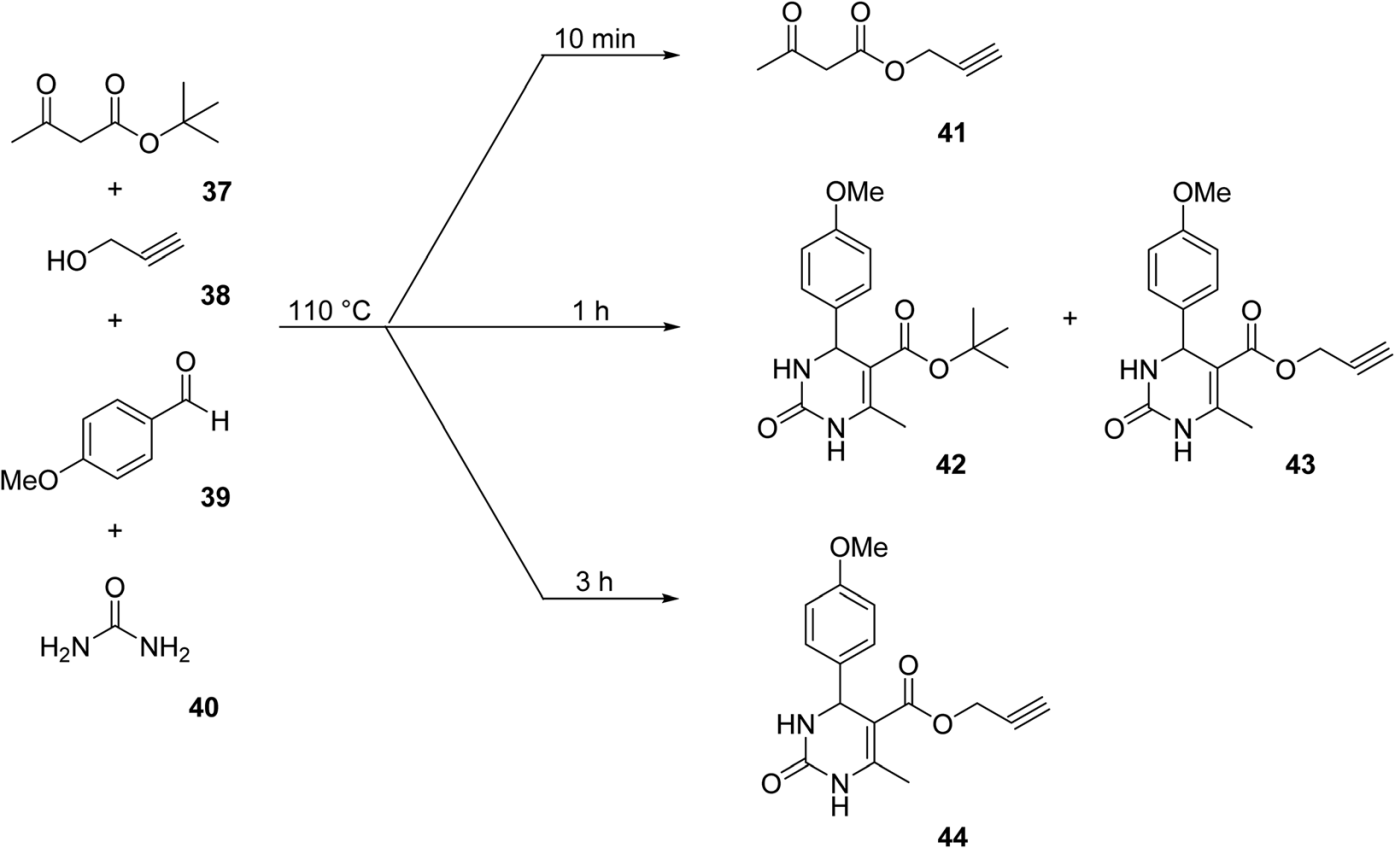
Pathways to dihydropyrimidin-2(1*H*)-one C5 ester derivatives.^129^

A catalyst-free, one-pot synthesis of dihydropyrimidinones was developed by Darma Rao and co-workers*.*^129,130^ This methodology involved the *in situ* generation of β-keto esters *via* transesterification followed by a Biginelli reaction with arylaldehyde and urea at 110 °C (Scheme 10). The best results were obtained when the β-keto ester, arylaldehyde, urea and propargyl alcohol were used in a 1 : 1 : 1.2 : 1.5 ratio. ESI-MS analysis confirmed the presence of the transesterified propargyl-β-keto ester product after ten minutes, the dihydropyrimidinone product and *tert*-butyl β-keto ester after 1 hour, and finally the desired dihydropyrimidinones after three hours. This one-pot preparation followed two main pathways: either transesterification followed by Biginelli reaction or *vice versa.* When the same conditions were applied to methyl, ethyl or isobutyl β-keto esters, the corresponding transesterified dihydropyrimidinones were obtained as expected.

The scope of this transesterification procedure was investigated across a range of alcohols (Table 57). In general, yields were good to excellent, but tertiary alcohols (entry 7) and unsaturated aliphatic alcohols (entries 14 and 15) were associated with a noticeable reduction in yields. The presence of amine (entry 5) or ether (entries 9 and 12) functional groups was tolerated under these conditions.

**Table 57:** Transesterification under solvent-free, catalyst-free conditions^130^

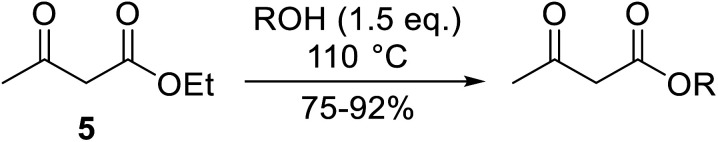			
Entry	R	Time (h)	Yield (%)
1	Et	3	92
2	^i^Bu	3	90
3	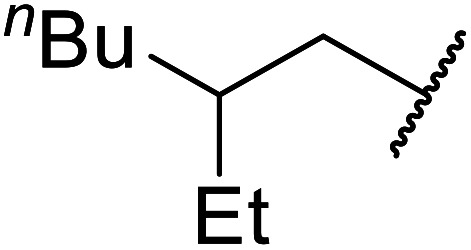	3	85
4	^*n*^Oct	3	85
5	Me_2_N(CH_2_)_2_	3	90
6	Ph	3	85
7	^*t*^Bu	5.5	60
8	Bn	3.5	88
9	4-MeO–C_6_H_4_CH_2_	3	90
10	4-Cl–C_6_H_4_CH_2_	4	85
11	Ph(CH_2_)_2_	3	83
12	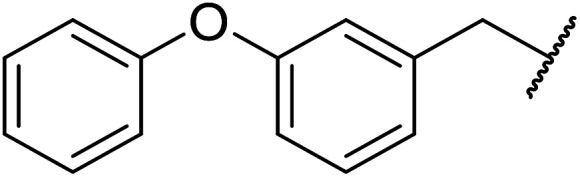	4	80
13	CH_2_=CHCH_2_	3	80
14	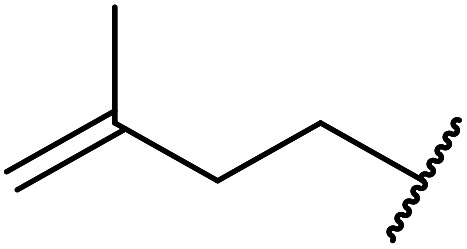	4	76
15	HC≡CCH_2_	3	75

The semi-synthesis of paclitaxel from 10-deacetylbaccatin III involves a key transesterification reaction. With this in mind, Mandai *et al.* developed a catalyst- and solvent-free methodology and expanded its scope to include various β-keto esters (Table 58).^2^ A large excess (20 equivalents) of the β-keto ester, heated to 90 °C for 24 hours, generated the target in 82% yield (entry 1). The reaction was greatly accelerated by continuous removal of ethanol under reduced pressure (entry 2). Transesterification proceeded at a reasonable rate at 70 °C (entry 3) but very slowly at 50 °C (entry 4) even under reduced pressure. Reducing the equivalents of β-keto ester from 20 to 5 led to only a slight reduction in yield (entry 5). However, a reduction to 2 equivalents caused a sharp decrease in yield (entry 6). In cases where R^2^ is aromatic, the reactivity or yields were not affected (entries 9–14). Cyclic and acyclic substituents were also well tolerated (entries 14–16). Methyl-substituted ethyl benzoylacetate reacted sluggishly to yield only 20% of product (entry 19).

**Table 58:** Transesterification of β-keto esters with protected derivatives of 10-deacetylbacctin III^2^

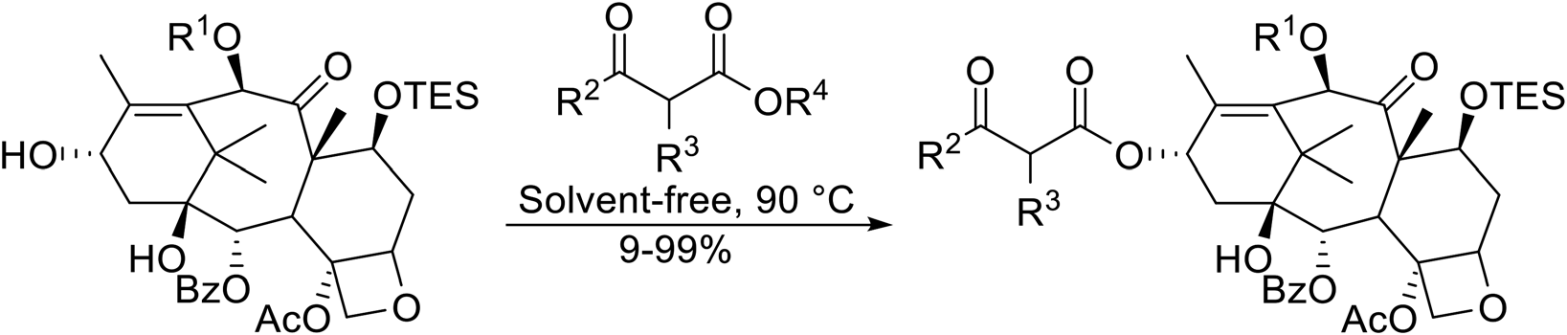								
Entry	R^1^	R^2^	R^3^	R^4^	β-Keto ester eq.	Pressure (mmHg)	Time (h)	Yield (%)
1	Cbz	Ph	H	Et	20	760	24	82 (14^a^)
2	Cbz	Ph	H	Et	20	<1	3	97
3^b^	Cbz	Ph	H	Et	20	<1	27	90 (5^a^)
4^c^	Cbz	Ph	H	Et	20	<1	21	9 (90^a^)
5	Cbz	Ph	H	Et	5	20	10	94
6	Cbz	Ph	H	Et	2	20	24	43 (54^a^)
7	Ac	Ph	H	Et	20	<1	3	91
8	Alloc	Ph	H	Et	20	<1	3	96
9	Cbz	4-MeO–C_6_H_4_	H	Me	10	20	7	99
10	Cbz	3-F–C_6_H_4_	H	Me	10	20	6	88
11	Cbz	2-F–C_6_H_4_	H	Me	10	20	6	92
12	Cbz	4-F–C_6_H_4_	H	Me	10	20	6	94
13	Cbz	3-CF_3_–C_6_H_4_	H	Me	10	20	6	90
14	Cbz	2-Furyl	H	Me	20^a^	20	8	99
15	Cbz	Cy	H	Me	10	20	6	95
16	Cbz	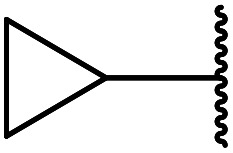	H	Me	10	20	6	94
17	Cbz	^*n*^CH_3_(CH_2_)_8_	H	Me	10	20	6	95
18	Cbz	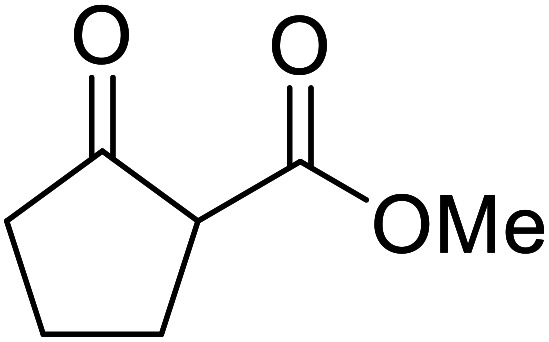			20^a^	20	5	93
19	Cbz	Ph	Me	Me	20	20	25	20^b^

a Recovery of starting material.

b Conducted at 70 °C.

c Conducted at 50 °C.

Chadha and co-workers discovered that reaction of β-keto esters with polyols under microwave irradiation afforded the corresponding monotransester in good to excellent yields (Table 59).^131^ Subsequent crosslinking led to the accumulation of the corresponding diester as a minor product. A molar ratio of 1 : 5 β-keto ester to polyol minimised the formation of the unwanted diester. The efficient absorption of microwave energy by the polar reactants saw full conversions in under 10 minutes in all cases. An increase in chain length was associated with longer reaction times and lower yields (entries 9–11). The unsaturated diol 1,4-butenediol also underwent selective mono-transesterification (entry 19). Accordingly, this method may be suitable for the selective mono-transesterification of polyols.

**Table 59:** Microwave-mediated transesterification of polyols^131^

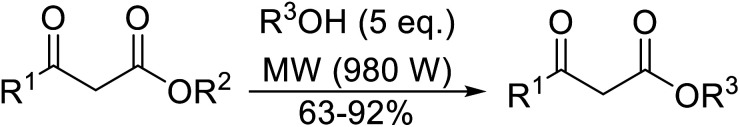					
Entry	R^1^	R^2^	R^3^	Time (min)	Yield (%)
1	Ph	Et	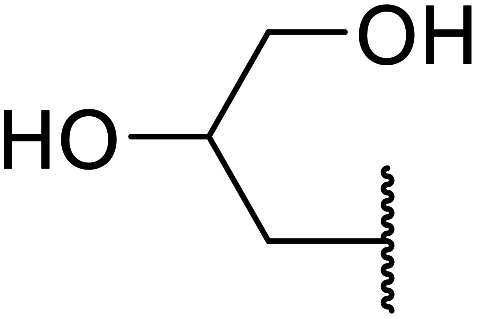	5	75
2	4-MeO–C_6_H_4_	Et	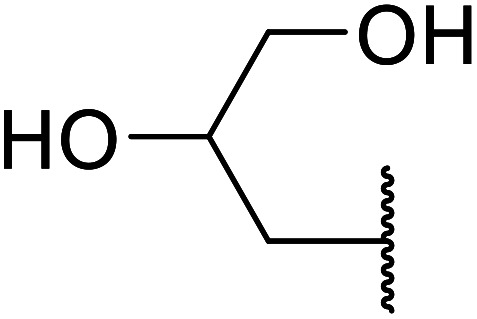	5	73
3	4-NO_2_–C_6_H_4_	Et	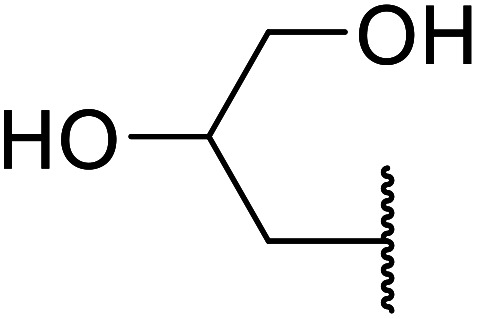	4	78
4	Me	Me	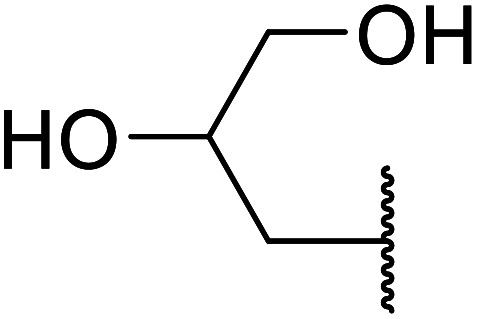	4	75
5	Me	Et	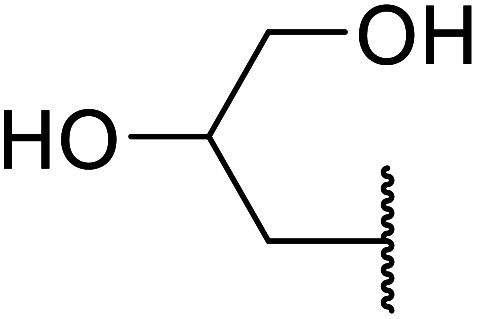	4	80
6	Me	^i^Pr	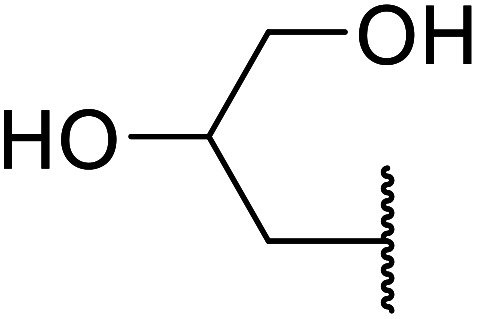	6	63
7	Me	^*t*^Bu	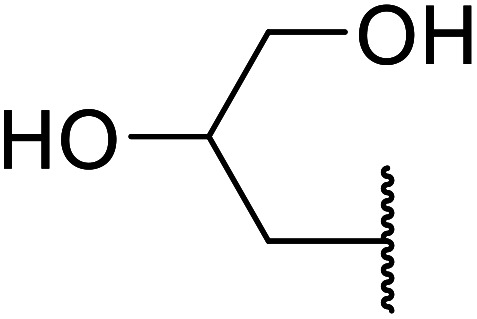	7	65
8	Ph	Et	HO(CH_2_)_2_	8	92
9	Me	Et	HO(CH_2_)_2_	10	89
10	Me	^i^Pr	HO(CH_2_)_2_	12	85
11	Me	^*t*^Bu	HO(CH_2_)_2_	14	82
12	Ph	Et	HO(CH_2_)_2_O(CH_2_)_2_	9	90
13	Me	Me	HO(CH_2_)_2_O(CH_2_)_2_	8	89
14	Me	Et	HO(CH_2_)_2_O(CH_2_)_2_	8	92
15	Me	^i^Pr	HO(CH_2_)_2_O(CH_2_)_2_	10	84
16	Me	^*t*^Bu	HO(CH_2_)_2_O(CH_2_)_2_	12	80
17	Ph	Et	HO(CH_2_)_4_	7	85
18	Ph	Et	HO(CH_2_)_5_	7	88
19	Ph	Et	HOCH_2_CH=CHCH_2_	8	85

## 10. Miscellaneous methods

Superacids are more acidic than sulfuric acid and have a proven track record in organic transformations.^132^ Chavan *et al.* have pioneered the use of the sulfonated stannous oxide, a solid super acid, for transesterification reactions (Table 60).^133^ This catalyst is selective for both β-keto esters (entries 1–6 and 8–10) and γ-keto esters (entry 7). However, γ-keto esters did require longer reaction times and returned lower yields. Transesterification of tertiary alcohols generally proceeded in lower yields (entries 9 and 10). Some of the limitations of this catalyst includes its high cost and difficulty of preparation.

**Table 60:** Catalysis using sulfonated stannous oxide^133^

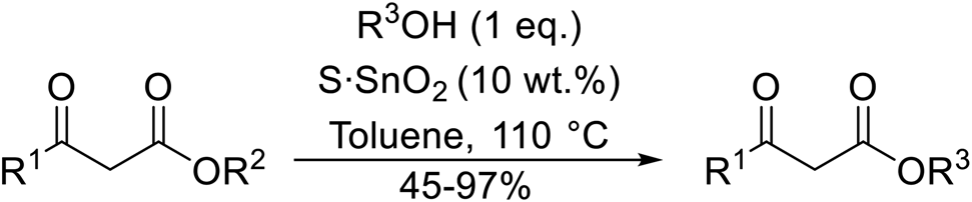					
Entry	R^1^	R^2^	R^3^	Time (h)	Yield (%)
1	Me	Me	^*n*^Bu	6	97
2	Me	Me	Menthyl	6	91
3	Me	Me	^*n*^Oct	7	89
4	Me	Me	Cy	7	84
5	Me	Me	Cl(CH_2_)_2_	6	92
6	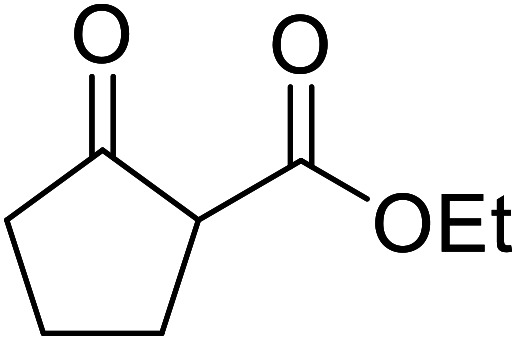		^*n*^Bu	7	63
7	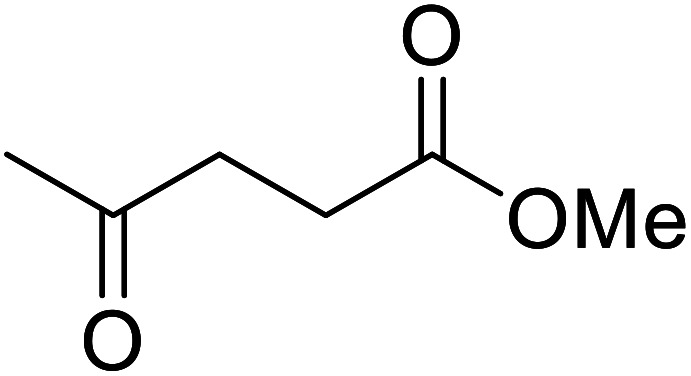		^*n*^Bu	10	45
8	Me	Me	H_2_C=CHCH_2_	8	65
9	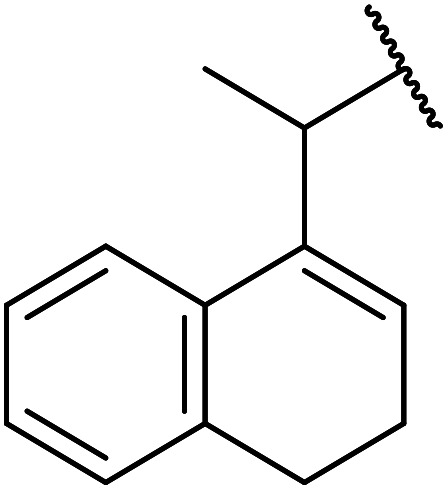	Et	^*t*^Bu	8	50
10	Me	Me	^*t*^Bu	12	50

Amberlyst-15 is an acidic ion exchange resin often employed in organic synthesis (Table 61).^134–137^ Amberlyst-15 successfully catalysed the transesterification of primary (entries 1–7 and 10–12), secondary (entries 8 and 9) and allylic alcohols (entry 10).^138^ Both β-ketoesters (entries 1–11) and γ-ketoesters (entry 12) were also transesterified. The conversion of *N*-(2-hydroxyethyl)phthalimide to 2-phthalimidoethyl acetoacetate ester (entry 1) is especially noteworthy, as the product is an important building block for the preparation of 1,4-dihydropyridine derivatives, which are calcium channel blockers. When mercaptoethanol was introduced, an equimolar amount of the thioester and ester products were recovered in good yields (entry 11).

**Table 61:** Amberlyst-15-catalysed transesterification^138^

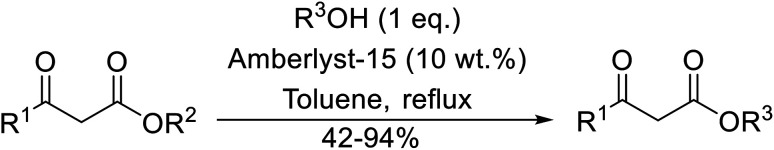					
Entry	R^1^	R^2^	R^3^	Time (h)	Yield (%)
1	Me	Me	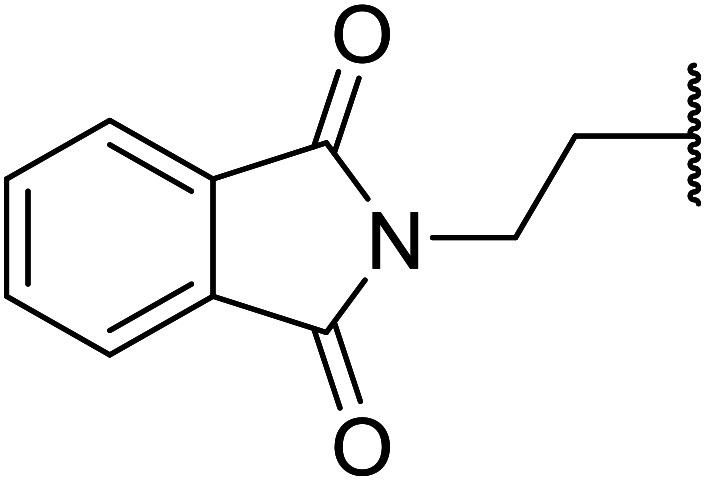	10.0	85
2	Me	Me	^*n*^Bu	5.5	85
3	Me	Me	^*n*^Oct	2.0	89
4	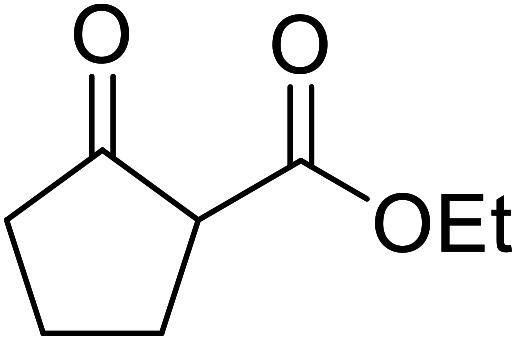		^*n*^Bu	3.0	90
5	Me	Me	Cl(CH_2_)_2_	3.0	75
6	Me	Me	Ph(CH_2_)_2_	4.0	88
7	Me	Me	Ph(CH_2_)_2_	2.0	64
8	Me	Me	Menthyl	6.0	94^a^
9	Me	Me	Cy	6.0	65
10	Me	Me	H_2_C=CCH_2_	8.0	42
11	Me	Me	HS(CH_2_)_2_	2.0	86^b^
12	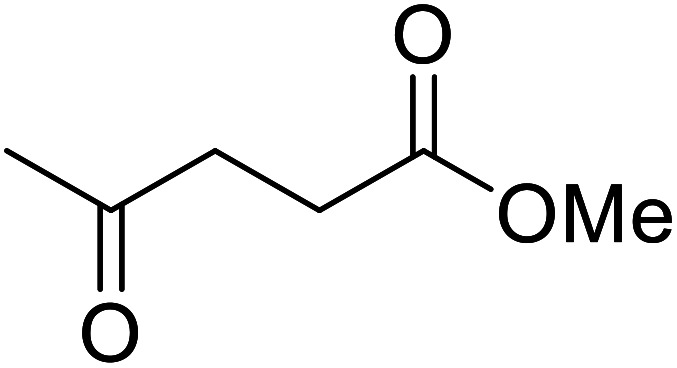		^*n*^Bu	10.0	54

a 2 equivalents of alcohol used.

b Combined yields of transthioesterification and transesterification products (1 : 1 ratio).

Sodium borohydride is commonly employed for the reduction of carbonyls and imines, but not esters. Padhi and Chadha have developed a room temperature, one-pot reduction/transesterification of β-keto esters using sodium borohydride (Table 62).^139^ Kinetic studies established that the reduction step precedes transesterification. When dissolved in an alcohol, sodium borohydride forms a Na^+^B^−^(H)_3_(OR)_1_ complex.^140,141^ The active hydride in this complex is responsible for the reduction of the carbonyl group. Transesterification of β-hydroxy esters is difficult due to the presence of the acid/alkali sensitive hydroxyl, and often necessitates exotic reagents or prolonged reaction times.^142–144^ Employing isopropanol led to a low 45% yield (entry 4). Propargyl alcohol also resulted in a poor yield of 25% (entry 15). These low yields are likely due to the poor solubility of sodium borohydride in these alcohols, leading to limited formation of the key alkoxy borate complex. Sodium borohydride-mediated reduction/transesterification is unique to β-keto esters. With α- and γ-keto esters, only the corresponding hydroxy esters were recovered. A similar reduction/transesterification reaction of α-keto esters employing a combination of sodium borohydride and cerium trichloride has been reported.^145^

**Table 62:** Reduction/transesterification of β-keto esters using sodium borohydride^139^

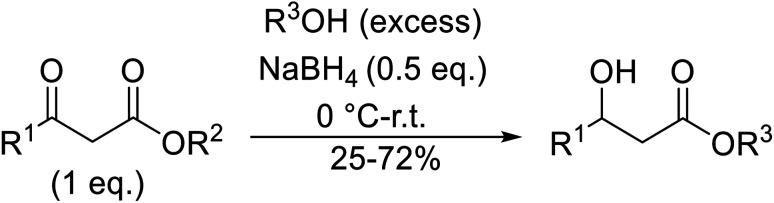					
Entry	R^1^	R^2^	R^3^	Time (h)	Yield (%)
1	Me	Me	Et	12	65^a^
2	Me	Me	^*n*^Pr	12	62^a^
3	Me	Me	^*n*^Bu	12	60^a^
4	Me	Me	^i^Pr	12	45^a^
5	Me	Et	Me	12	72
6	Me	Et	^*n*^Pr	12	65
7	Me	Et	^*n*^Bu	12	61
8	Me	^*n*^Bu	Me	12	65
9	Me	^*n*^Bu	Et	12	62^a^
10	Me	^*n*^Bu	^*n*^Pr	12	61^a^
11	Ph	Et	Me	18	64^a^
12	Ph	Et	^*n*^Pr	18	62^a^
13	Ph	Et	^*n*^Bu	18	60^a^
14	Ph	Et	H_2_C=CHCH_2_	18	72
15	Ph	Et	HC≡CCH_2_	18	25

a An increase of 2–21% in yield was observed when a large excess of alcohol and longer reaction time was used.

Yadav *et al.* discovered that triphenylphosphine facilitates the transesterification of β-keto esters (Table 63).^146^ In all cases, the reactions reached completion within 6–8 hours. Challenging substrates, such as propargyl alcohol (entry 1), sterically hindered menthol (entry 5) and prenol (entry 9), reacted readily to give products in excellent yields. In the case of heptyl alcohol, transesterification of an aromatic ester (entry 3) was faster than for an aliphatic ester (entry 14). A similar trend was noted for decenyl alcohol (entry 4 *vs.* 15). The reaction of benzyl alcohol with a cyclic ester (entry 11) was slower than with an aliphatic ester (entry 10). This confirms that aromatic esters are more reactive than aliphatic esters, which in turn are more reactive than cyclic esters.

**Table 63:** Triphenylphosphine-mediated transesterification^146^

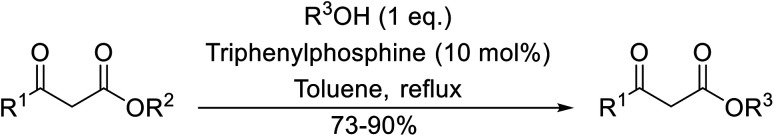					
Entry	R^1^	R^2^	R^3^	Time (h)	Yield (%)
1	Me	Me	CH≡CCH_2_	6.0	90
2	Me	Me	^*n*^Bu	6.5	87
3	Me	Me	^*n*^Hep	7.0	79
4	Me	Me	H_2_C=CH(CH_2_)_8_	6.5	84
5	Me	Me	Menthyl	6.5	90
6	Me	Et	H_2_C=CHCH_2_	8.0	78
7	Me	Et	Cl(CH_2_)_2_	7.0	84
8	Me	Et	Ph(CH_2_)_2_	6.0	90
9	Me	Et	(CH_3_)_2_C=CHCH_2_	6.5	90
10	Me	Et	Bn	6.0	78
11	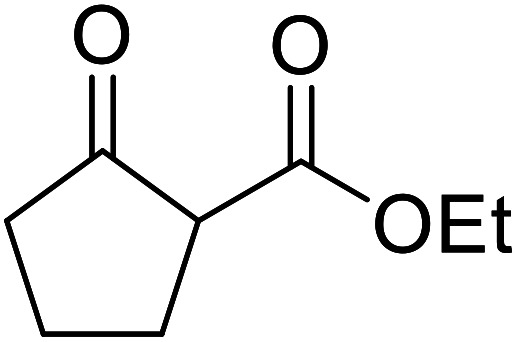		Bn	8.0	73
12	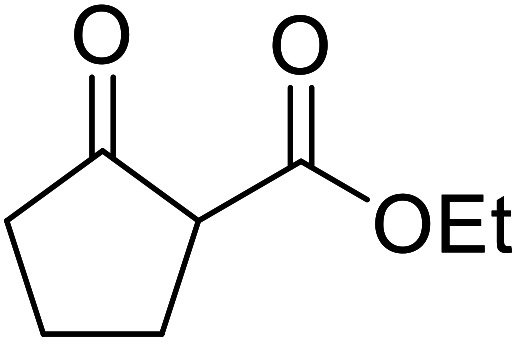		H_2_CCHCH_2_	7.0	84
13	Ph	Et	H_2_C=CHCH_2_	6.0	87
14	Ph	Et	^*n*^Hep	6.5	88
15	Ph	Et	H_2_C=CH(CH_2_)_8_	6.0	86

In recent years, the application of ionic liquids to organic transformations has gained considerable interest.^147^ Bhanage and co-workers investigated *N*-methyl-2-pyrrolidone hydrogen sulfate ([NMP]^+^HSO_4_^−^) as an efficient and reusable catalyst for the transesterification of β-ketoesters.^148^ This ionic liquid has several important advantages over other systems including high catalytic activity. Additionally, *N*-methyl-2-pyrrolidone is more economical and accessible than other imidazolium/pyridinium counterparts. β-Ketoesters were smoothly transesterified in high yields with easy separation of the ionic liquid and the products (Table 64). Transesterification of methyl acetoacetate with aliphatic alcohols furnished yields of between 74% and 91% (entries 1–7). Similar results were obtained with ethyl acetoacetate (entries 12–14). [NMP]^+^HSO_4_^−^ produced good yields with various unsaturated alcohols (entries 9–11 and 16) and with no evidence of Carroll rearrangement. Four successive runs with the recycled catalyst saw only a slight decrease in yields.

**Table 64:** [NMP]^+^HSO_4_^−^ catalysis^148^

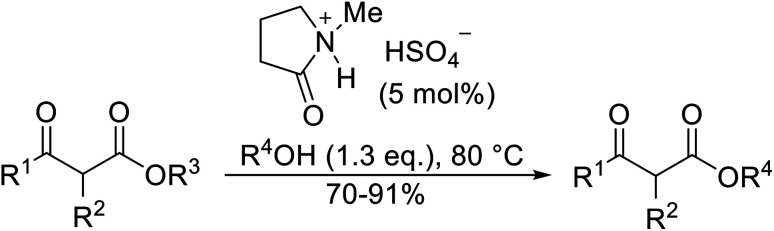							
Entry	R^1^	R^2^	R^3^	R^4^	Time (h)	Conversion^a^ (%)	Yield^a^ (%)
1	Me	H	Me	^*n*^Bu	3	80	79
2	Me	H	Me	^*n*^Hex	3	93	90
3	Me	H	Me	^*n*^Hep	3	94	89
4	Me	H	Me	^*n*^Oct	3	92	91
5	Me	H	Me	^i^Bu	3	88	84
6	Me	H	Me	^i^Pr	3.5	87	74
7	Me	H	Me	Cy	4	94	85
8	Me	H	Me	Bn	3.5	89	76
9	Me	H	Me	PhCH=CHCH_2_	3	91	87
10	Me	H	Me	CH_3_CH=CHCH_2_	3	95	90
11	Me	H	Me	H_2_C=CHCH_2_	3	92	86
12	Me	H	Et	^*n*^Bu	3	83	78
13	Me	H	Et	^*n*^Hex	3	94	85
14	Me	H	Et	^*n*^Oct	3	93	84
15	Me	H	Et	Bn	3.5	91	70
16	Me	H	Et	H_2_C=CHCH_2_	3	94	80
17	Me	Me	Et	^*n*^Hex	3.5	89	87
18	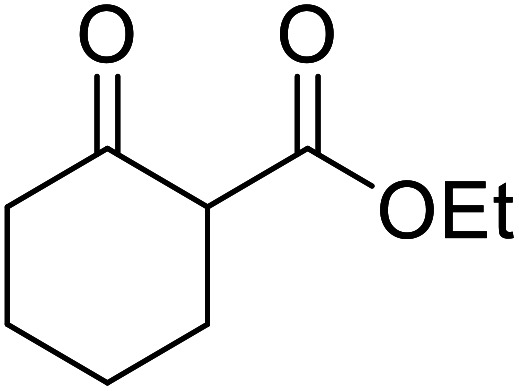			^*n*^Bu	3.5	90	84
19	Ph	H	Et	^*n*^Oct	3.5	88	83

a Based on GC analysis.

1-Propyl-3-methylimidazole chloride ([C_3_MIm]Cl) in sulfamic acid has been studied as a recyclable catalytic medium for the transesterification of methyl acetoacetate (Table 65).^149^ These conditions were compatible with a wide range of structurally diverse substrates including aliphatic (entries 1–4), cyclic (entry 5), unsaturated (entries 6 and 7), aromatic (entries 8 and 9) and sterically crowded (entry 3) alcohols. This catalyst system was reused five times with only a slight decrease in yield noted (entry 1). A disadvantage of this approach is the requirement for large quantities of the ionic liquid (10 g ionic liquid for 1 g sulfamic acid).

**Table 65:** [C_3_MIm]Cl/sulfamic acid-catalysed transesterification of methyl acetoacetate^149^

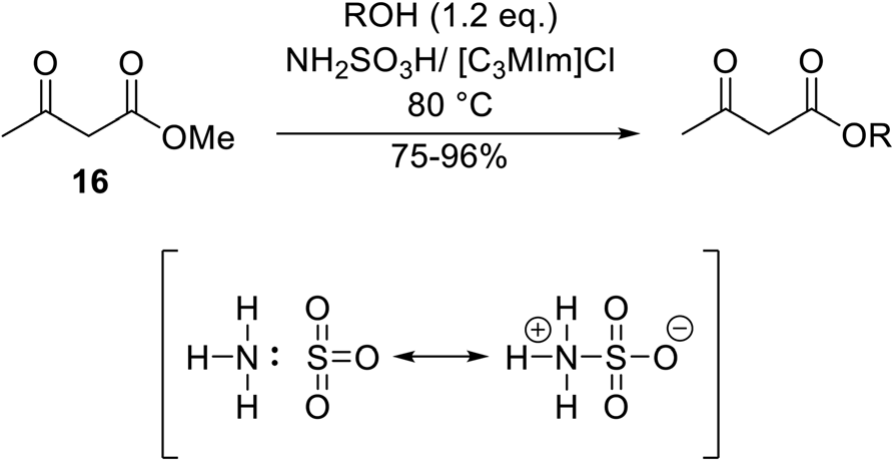			
Entry	R	Time (h)	Yield^a^ (%)
1	^*n*^Bu	3.0	96 (86^b^)
2	^*sec*^Bu	3.0	95
3	^*t*^Bu	4.5	75
4	^*n*^Hex	3.0	95
5	Cy	3.0	94
6	H_2_C=CHCH_2_	3.0	93
7	HC≡CCH_2_	4.0	92
8	Bn	4.0	93
9	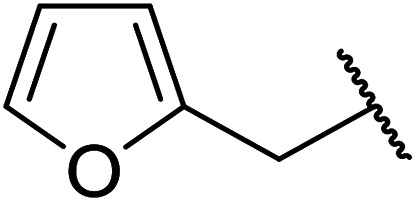	4.0	93

a GC yield.

b Yield after 5 cycles.

Weber *et al.* have recently reported the synthesis of long chain acetoacetates *via* sulfamic acid catalysis under solvent-free conditions (Table 66).^150^ Using microwave irradiation, the reaction time was reduced from 6 hours to 13 minutes. The yields obtained *via* conventional heating or microwave irradiation were comparable. Furthermore, the catalytic activity of sulfamic acid was maintained, even after three cycles.

**Table 66:** Sulfamic acid-catalysed transesterification of methyl acetoacetate^150^

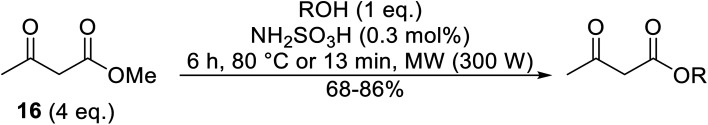			
Entry	R	Conventional heating yield (%)	MW irradiation yield (%)
1	^*n*^Hex	79	81
2	^*n*^Oct	77	80
3	^*n*^CH_3_(CH_2_)_9_	68	75
4	^*n*^CH_3_(CH_2_)_11_	69	73
5	^*n*^CH_3_(CH_2_)_13_	69	75
6	^*n*^CH_3_(CH_2_)_15_	83	85
7	^*n*^CH_3_(CH_2_)_17_	85	86
8	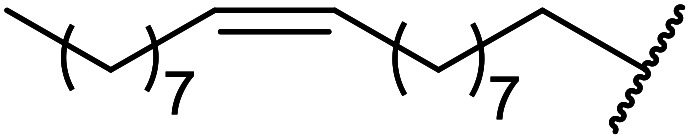	78	80
9	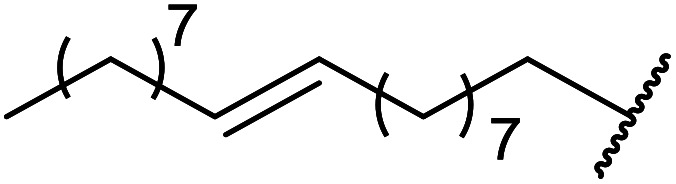	73	75
10	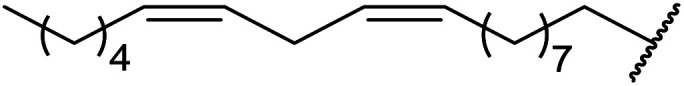	75	79
11	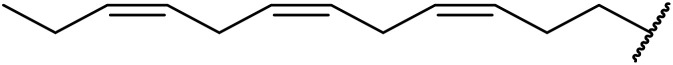	72	76

*para*-Toluene sulfonic acid (*p*-TSA) catalyses both the esterification of β-keto acids and the transesterification of β-keto esters in comparable yields (Table 67).^151^ Good yields were achieved with aliphatic (entries 1–7 and 10–12) and aromatic (entries 8 and 9) alcohols, but bulkier substrates such as *tert-*butanol (entry 5 and 11) furnished lower yields. Substituting methyl acetoacetate (entries 1–8) with ethyl acetoacetate (entries 9–12) afforded near identical yields.

**Table 67:** Catalysis with *para*-toluene sulfonic acid^151^

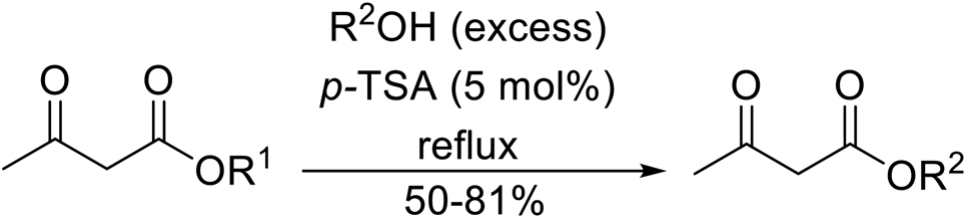			
Entry	R^1^	R^2^	Yield (%)
1	Me	^*n*^Pr	71
2	Me	^i^Pr	65
3	Me	Cy	72
4	Me	^*sec*^Bu	73
5	Me	^*t*^Bu	50
6	Me	^*n*^Bu	78
7	Me	^*n*^Oct	81
8	Me	Bn	71
9	Et	Bn	70
10	Et	^*n*^Bu	80
11	Et	^*t*^Bu	51
12	Et	Cy	68

*N*-Bromosuccinimide (NBS) is a standard brominating agent which may also be exploited for the selective transesterification of β-keto esters (Table 68).^152^ Structurally diverse β-keto esters including alkyl (entries 1–10, 16), cyclic (entries 11–15) and aromatic (entries 17–22) esters were readily transformed. The substrate scope also encompassed thiols (entry 23) and amines (entry 24). The hydroxyl group of 2-mercaptoethanol reacted preferentially (entry 25). In the case of amino alcohols, reaction at the more nucleophilic amine afforded the corresponding amide in 79% yield (entry 26). Diol substrates were converted to the diester (entry 27). The authors also investigated related reagents and found that *N-*chlorosuccinimide (NCS) was an equally effective catalyst. By contrast, *N-*chloronicotinamide (NCN) returned incomplete conversions and poor yields.

**Table 68:** *N*-Bromosuccinimide catalysis^152^

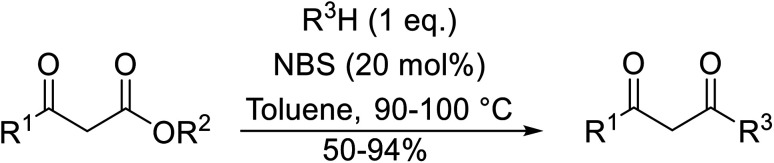					
Entry	R^1^	R^2^	R^3^	Time (h)	Yield (%)
1	Me	Me	PrO	3	94
2	Me	Et	PrO	3	92
3	Me	Pr	BuO	3	93
4	Me	Bu	PrO	3	94
5	Me	Et	CyO	4	88
6	Me	Et	Menthyl	4	89
7	Me	Et	^*t*^BuO	8	55
8	Me	Et	4-MeO–C_6_H_4_CH_2_O	4	82
9	Me	Et	PhCH=CHCH_2_O	4.5	86
10	Me	Et	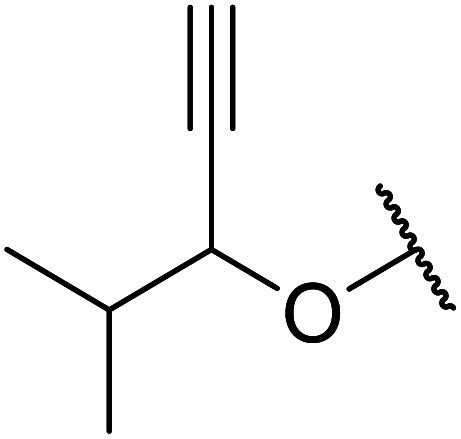	3.5	80
11	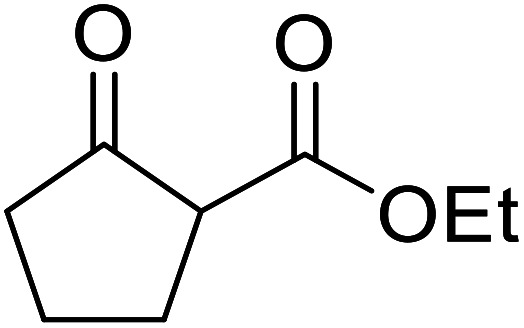		BuO	4	87
12	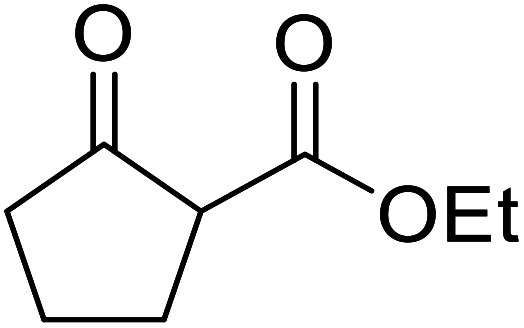		Menthyl	4	72
13	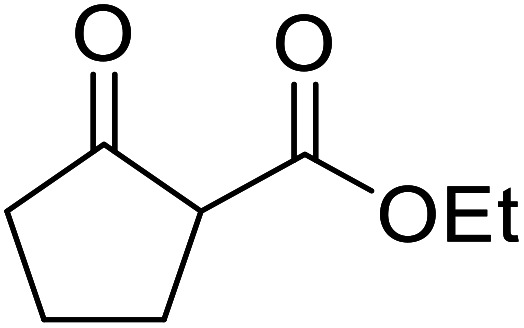		Ph_3_CO	8	52
14	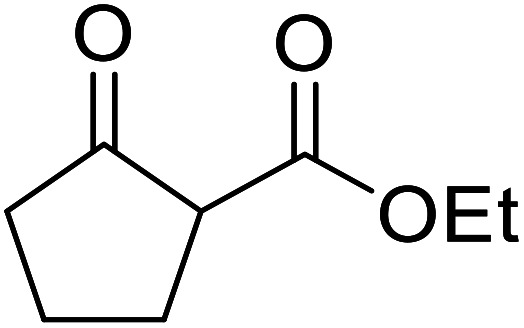		PhCHCHCH_2_O	4	85
15	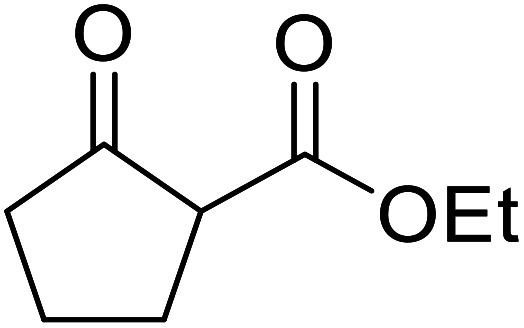		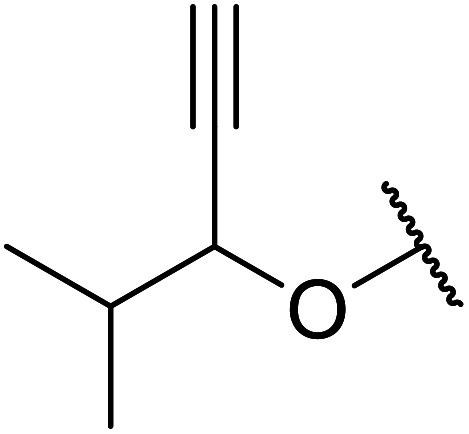	4	83
16	Me	Menthyl	BuO	8	50
17	Ph	Et	PhCH=CHCH_2_O	6	84
18	2,3,4-(MeO)_3_–C_6_H_2_	Et	PhCH=CHCH_2_O	7	81
19	2,3,4-(MeO)_3_–C_6_H_2_	Et	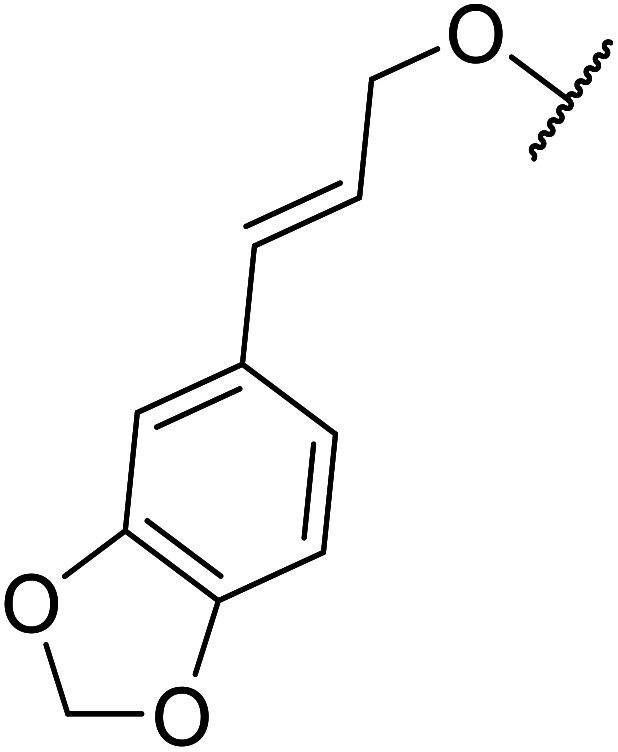	7	81
20	Ph	Et	Menthyl	4	77
21	Ph	Et	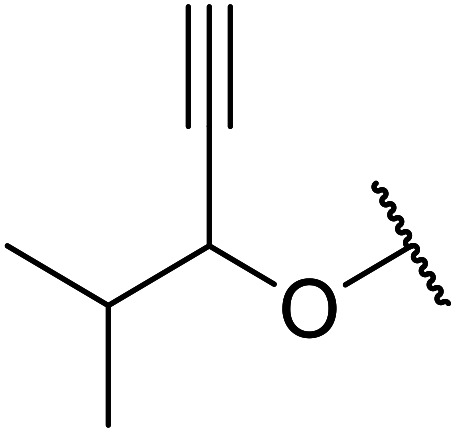	4	77
22	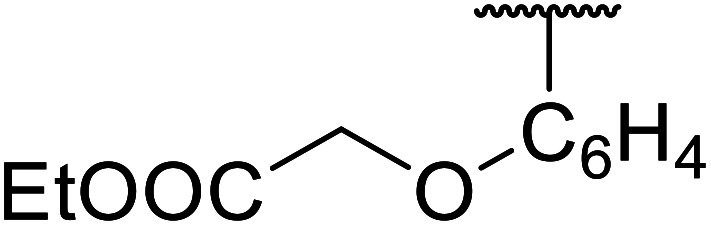	Et	PhCHCHCH_2_O	2	77
23	Me	Et	4-Cl–C_6_H_4_S	6	75
24	Me	Et	3-Cl–C_6_H_4_NH	4	80
25	Me	Et	HS(CH_2_)_2_O	4	89
26	Me	Et	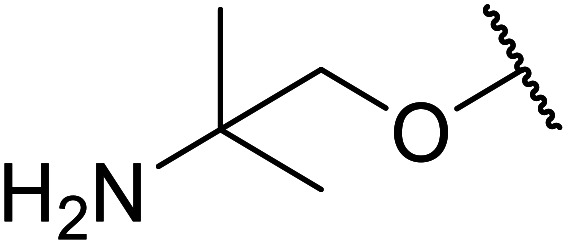	4	79
27	Me	Et	HO(CH_2_)_2_O	4	80

Building upon their previous work involving iodine-activated zinc catalysis (Table 32), Chavan *et al.* subsequently reported how iodine alone behaves as an efficient transesterification catalyst (Table 69).^153^ Transesterification of β-keto esters with benzyl (entry 11), allylic (entry 9) and propargyl (entry 10) alcohols proceeded in moderate to high yields. In most cases, 1.2 equivalents of alcohol proved sufficient. However, in the case of volatile alcohols, such as *n*-propanol (entry 6) and isopropanol (entry 7), two equivalents were required. The diester product was generated when a diol was introduced (entry 3). Phenols failed to react with either methyl acetoacetate or ethyl acetoacetate. By contrast, the zinc/iodine-catalysed transformation of phenols was successful (Scheme 7).

**Table 69:** Iodine-mediated catalysis^153^

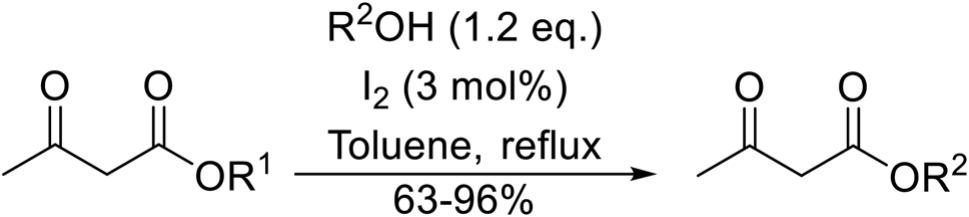					
Entry	R^1^	R^2^	Alcohol (eq.)	Time (h)	Yield (%)
1	Me	(−)-Menthyl	1.2	4	96
2	Me	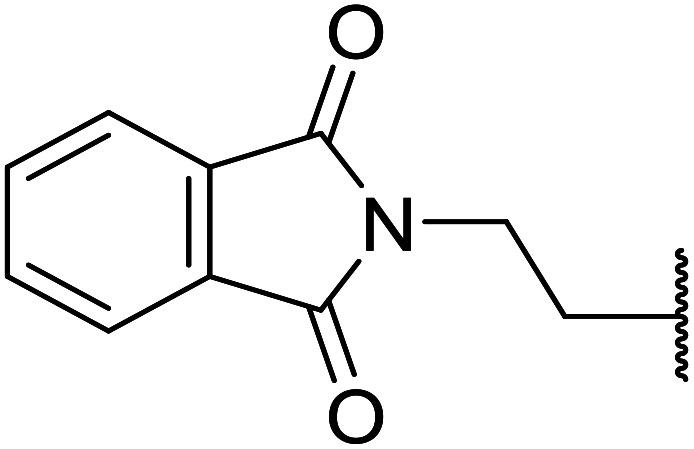	1.2	6.5	87
3	Me	HO(CH_2_)_10_	0.5	7	79^a^
4	Me	^*n*^CH_3_(CH_2_)_10_	1.2	5	86
5	Me	^*n*^Bu	1.2	5	81
6	Me	^*n*^Pr	2	7	65
7	Me	^i^Pr	2	7	63
8	Me	^*n*^Hep	1.2	5	89
9	Me	(CH_3_)_2_C=CHCH_2_	1.2	4	74
10	Me	HC≡CCH_2_	2	6.5	80
11	Et	Bn	1.2	5	83
12	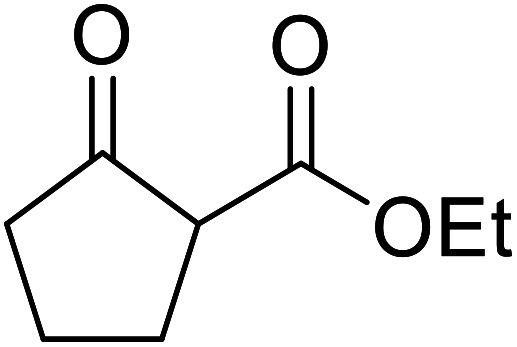	Bn	1.2	5	88
13	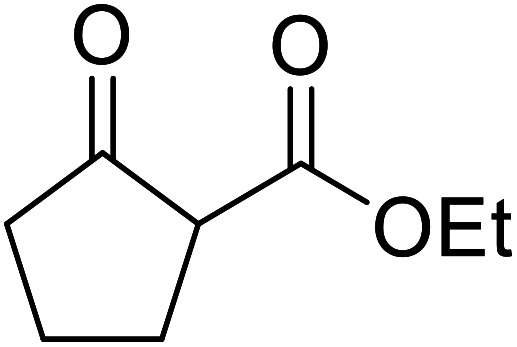	(−)-Menthyl	1.2	4.5	92
14	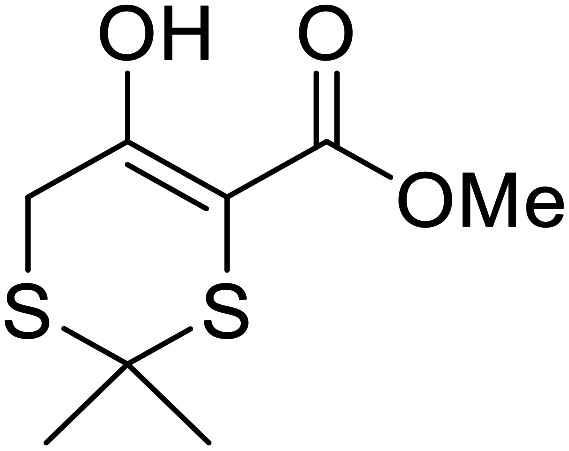	(−)-Menthyl	1.2	4	96

a Dimer product formed.

A mixture of polyethylene glycol (PEG) ionic liquids and toluene may display both homogeneous and heterogeneous phases at different temperatures (*e.g.*, bi-phasic conditions at lower temperatures and mono-phasic at higher temperatures). Ren and Cai investigated the transesterification of ethyl and methyl esters using a catalytic amount of iodine in PEG ionic liquid (Table 70).^154^ The authors found that heating to reflux was essential as the reaction was sluggish and low yielding at room temperature. β-Ketoesters were produced in good yields from aliphatic (entries 1–7 and 9–11) and aromatic (entries 8 and 12) alcohols. The reaction of *tert*-butanol afforded the corresponding ester in only moderate yields (entries 5 and 10). The catalyst could be reused four times with satisfactory results.

**Table 70:** Transesterification of β-keto esters using iodine in PEG ionic liquid^154^

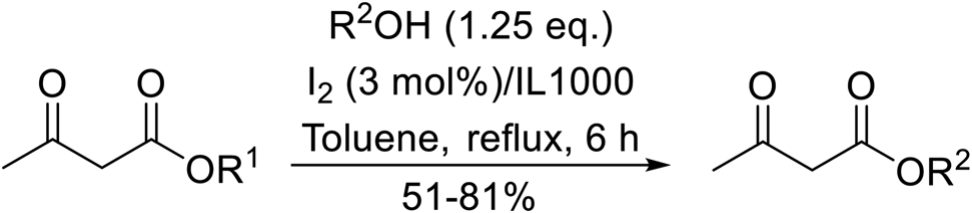			
Entry	R^1^	R^2^	Yield (%)
1	Et	^*n*^Pr	70
2	Et	^i^Pr	68
3	Et	^*n*^Bu	80
4	Et	^*sec*^Bu	76
5	Et	^*t*^Bu	51
6	Et	Cy	71
7	Et	^*n*^Oct	80
8	Et	Bn	75
9	Me	^*n*^Bu	81
10	Me	^*t*^Bu	53
11	Me	Cy	70
12	Me	Bn	72

Bandgar *et al.* compared sodium periodate, potassium periodate and anhydrous calcium chloride as potential transesterification catalysts (Table 71).^155^ The transformation of bulky tertiary alcohols (entries 4, 5 and 10) and unsaturated alcohols (entries 2, 4, 7 and 12), which are often problematic substrates in acid-catalysed reactions, was successfully realised by these catalysts albeit in modest yields. Of the three catalysts studied, potassium periodate generally afforded the highest yields and shortest reaction times. This methodology is highly specific for β-keto esters and other esters (*e.g.*, α-keto esters) remained unreacted. Amine, phenol or thiol nucleophiles proved similarly unreactive.

**Table 71:** Comparison of sodium periodate, potassium periodate and anhydrous calcium chloride catalysis^155^

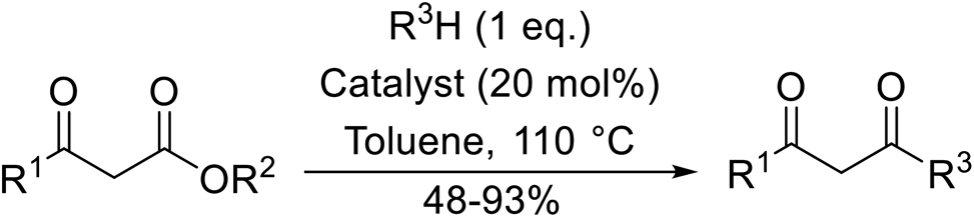									
Entry	R^1^	R^2^	R^3^	NaIO_4_		KIO_4_		CaCl_2_	
				Time (h)	Yield (%)	Time (h)	Yield (%)	Time (h)	Yield (%)
1	Me	Et	BnO	2	84	0.75	93	3	81
2	Me	Et	PhCH=CHCH_2_O	2	83	66	78	3	76
3	Me	Et	Menthyl	3	76	2.15	92	4	69
4	Me	Me	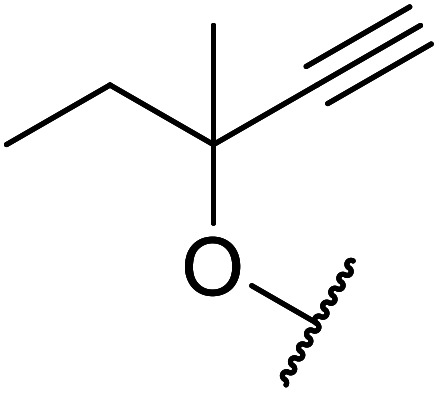	6	62	6	69	6	58
5	Me	Me	^*t*^BuO	6	55	6	67	6	48
6	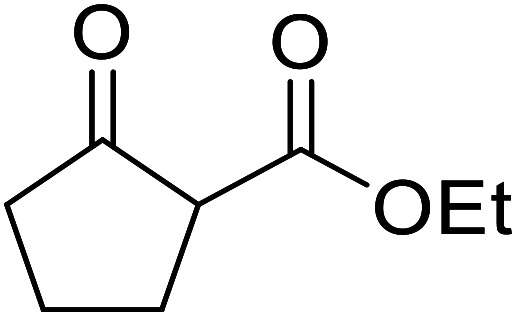		BnO	0.5	81	0.5	83	3	78
7	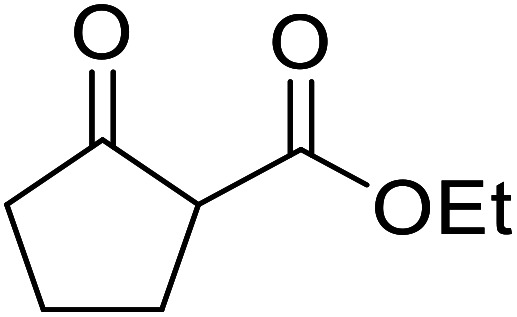		PhCHCHCH_2_O	1.5	81	1	83	5	76
8	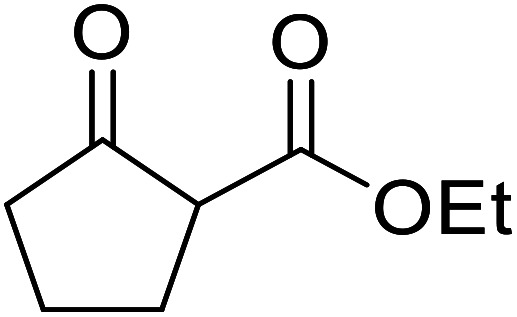		Menthyl	1.25	74	0.75	75	5	69
9	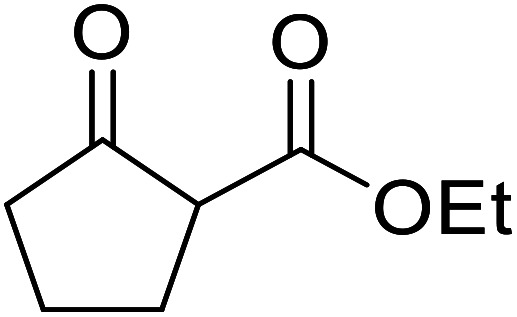		^*n*^BuO	1.25	77	0.75	81	3	68
10	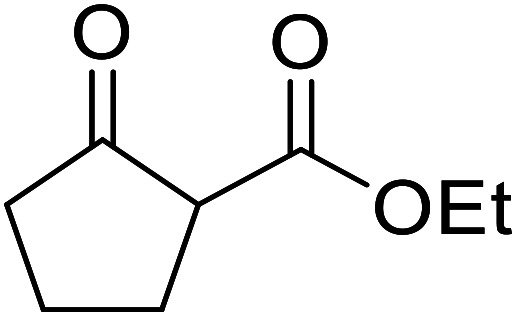		^*t*^BuO	3.5	48	3	56	6	48
11	Ph	Et	BnO	3.5	78	3	81	6	70
12	3,4,5-(MeO)_3_–C_6_H_2_	Et	PhCH=CHCH_2_O	3.5	78	3	81	6	80
13	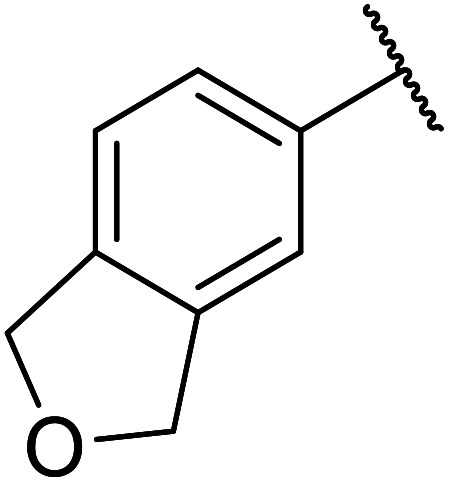	Et	Menthyl	2	76	1.5	82	4	56

In related work, Bandgar examined the catalytic activity of sodium perborate^156^ and lithium perchlorate^157^ (Table 72). Both catalysts were selective for β-keto esters (entries 1–12). The yields were comparable for both catalysts across all substrates tested (entries 1–12). However, sodium perborate is cheaper and less acidic than lithium perchlorate. A notable drop in yield was recorded for the bulky alcohol triphenylmethanol (entry 7).

**Table 72:** Sodium perborate or lithium perchlorate catalysis^156,157^

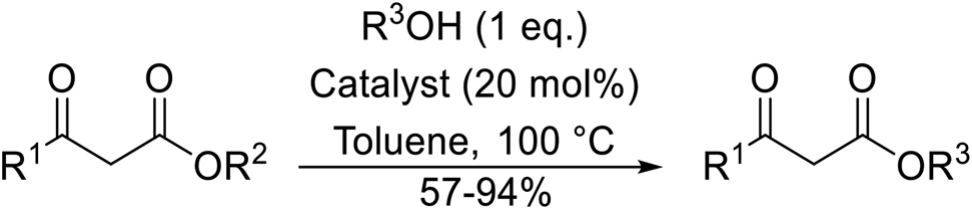							
Entry	R^1^	R^2^	R^3^	Sodium perborate		Lithium perchlorate	
				Time (h)	Yield (%)	Time (h)	Yield (%)
1	Me	Et	^*n*^Pr	2	90	2	93
2	Me	Et	^*n*^Bu	2	88	2	88
3	Me	Et	Bn	2	91	2	94
4	Me	Me	Cy	3	85	2.5	89
5	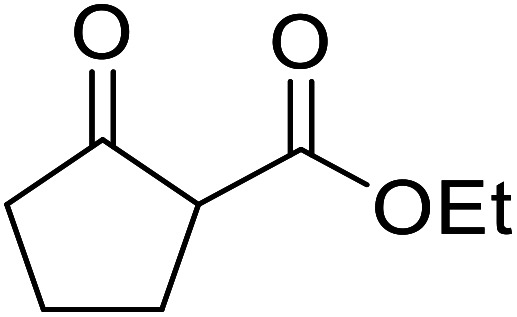		Menthyl	3.5	82	3.5	79
6	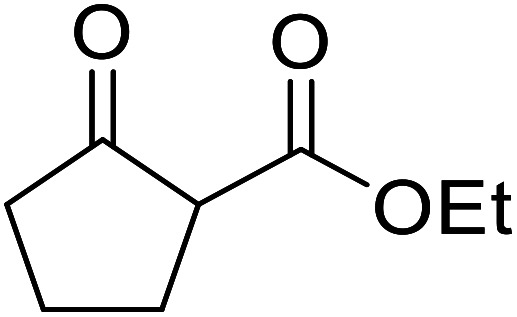		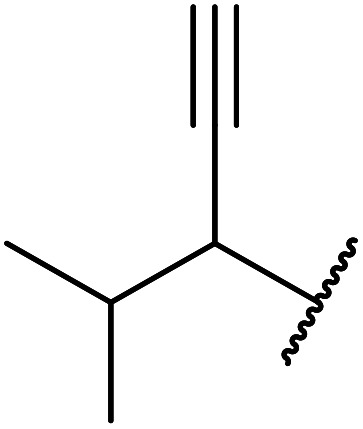	2.5	88	3	73
7	Me	Et	Ph_3_C	8	58	8	57
8	Me	Et	PhCH=CHCH_2_	3	80	3	65
9	Ph	Et	PhCH=CHCH_2_	4	85	6	82
10	3,4,5-(MeO)_3_–C_6_H_2_	Me	PhCH=CHCH_2_	4	88	6	77
11	3,4,5-(MeO)_3_–C_6_H_2_	Me	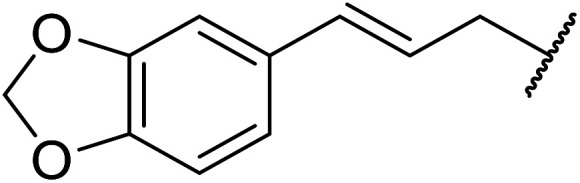	4	87	6	71
12	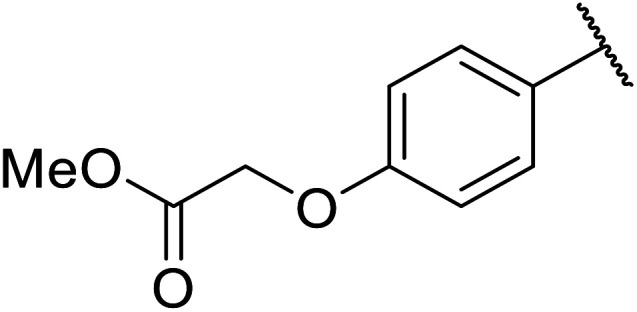	Et	PhCHCHCH_2_	3.5	81	7	76

The effectiveness of caesium fluoride as a transesterification catalyst was investigated by Inahashi and colleagues.^158^ Caesium chloride, caesium bromide and caesium iodide were examined under identical conditions, and caesium fluoride was found to be the superior catalyst. The optimal loading of caesium fluoride was 10 mol% (Table 73), but quantitative transesterification was observed with 2 mol% albeit at the cost of longer reaction times (entry 2). Acyclic (entries 1, 3–6 and 11), cyclic (entries 7 and 8) and aromatic esters (entries 9 and 10) furnished the desired products in excellent yields. The non-enolisable β-keto ester methyl 2,2-dimethyl-3-oxobutanoate was readily converted in 93% yield on increasing the catalyst loading to 10 mol% (entry 26). Allylic (entries 12–14), propargylic (entry 15), acid-sensitive (entries 14, 16 and 17) and base-sensitive (entry 18) alcohols reacted smoothly in good yields. Alcohols carrying tertiary amines were also transesterified in good yields (entries 20 and 21). Additionally, sterically hindered secondary alcohols, such as borneol (entry 22) and menthol (entry 23), as well as less reactive tertiary alcohols (entries 24 and 25), afforded the corresponding β-keto esters in moderate to good yields. The catalyst could be reused at least ten times without any appreciable decrease in activity.

**Table 73:** Transesterification using caesium fluoride^158^

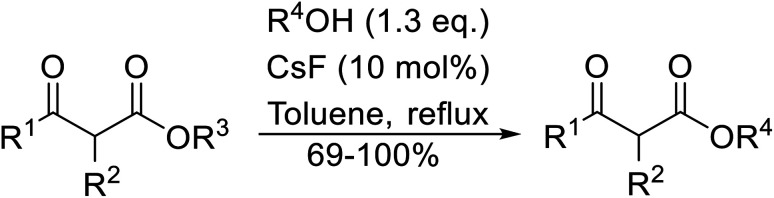						
Entry	R^1^	R^2^	R^3^	R^4^	Time (h)	Yield (%)
1	Me	H	Me	^*n*^Oct	18	93
2	Me	H	Me	^*n*^Oct	28	93^a^
3	Me	H	Et	^*n*^Oct	22	88
4	Me	H	^i^Pr	^*n*^Oct	4	81
5	^*n*^Pr	H	Et	^*n*^Oct	6	86
6	Me	Me	Et	^*n*^Oct	17	84
7	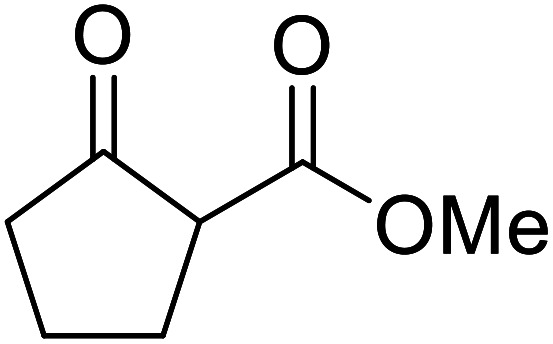			^*n*^Oct	2	93
8	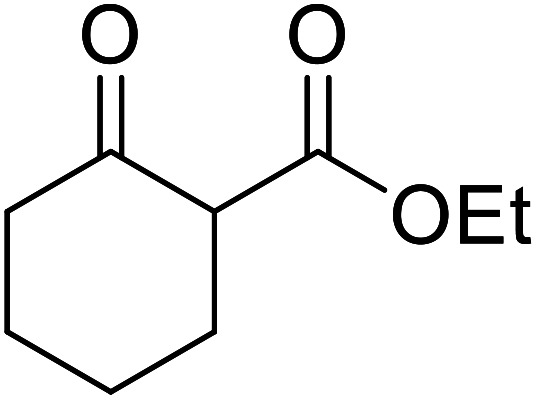			^*n*^Oct	9	93
9	Ph	H	Et	^*n*^Oct	6.5	100
10	4-MeO–C_6_H_4_	H	Et	^*n*^Oct	5	82
11	^*n*^Pr	H	Et	^*n*^Oct	6	86
12	^*n*^Pr	H	Et	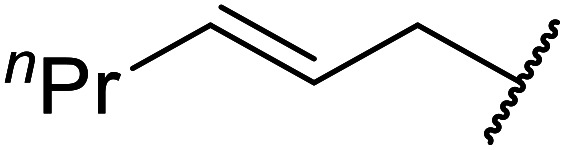	13.5	81
13	^*n*^Pr	H	Et	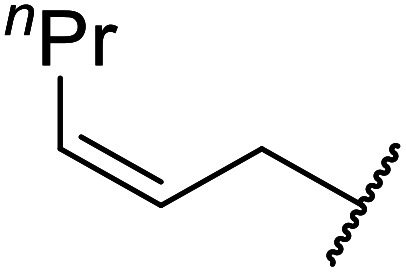	8.5	96
14	^*n*^Pr	H	Et	Geraniol	10.5	80
15	^*n*^Pr	H	Et	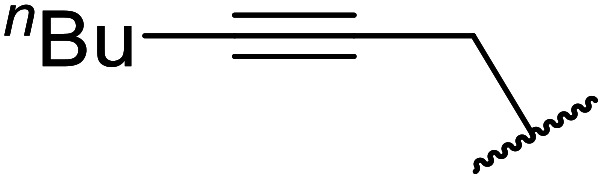	18	93
16	^*n*^Pr	H	Et	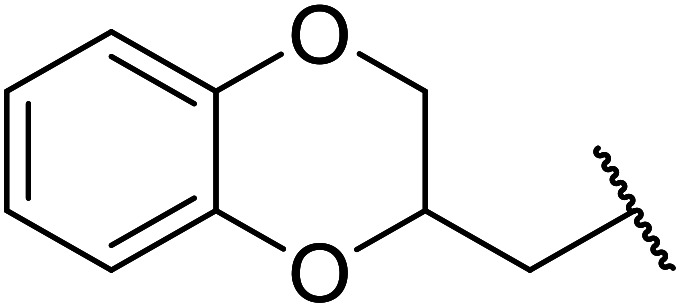	27	85
17	^*n*^Pr	H	Et	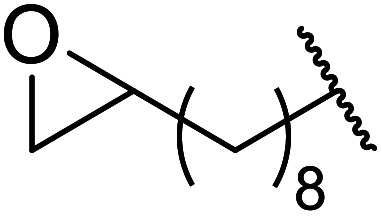	18	82
18	^*n*^Pr	H	Et	Cl(CH_2_)_6_	28	87
19	^*n*^Pr	H	Et	^*t*^BuMe_2_SiO(CH_2_)_6_–	8	80
20	^*n*^Pr	H	Et	Me_2_N(CH_2_)_2_	30	79
21	^*n*^Pr	H	Et	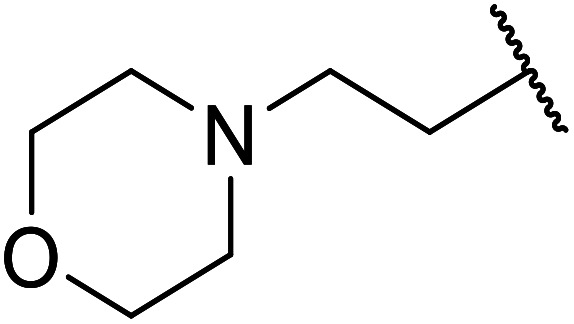	32	80
22	^*n*^Pr	H	Et	Borneol	25	82
23	^*n*^Pr	H	Et	Menthol	43	88
24	^*n*^Pr	H	Et	1-Adamantol	38	69
25	^*n*^Pr	H	Et	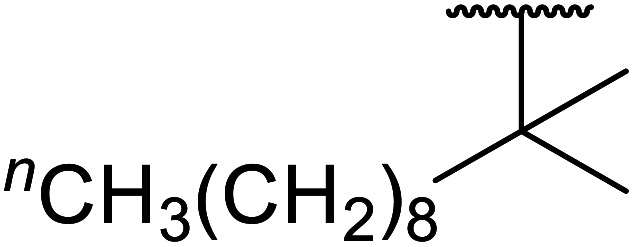	40	73
26	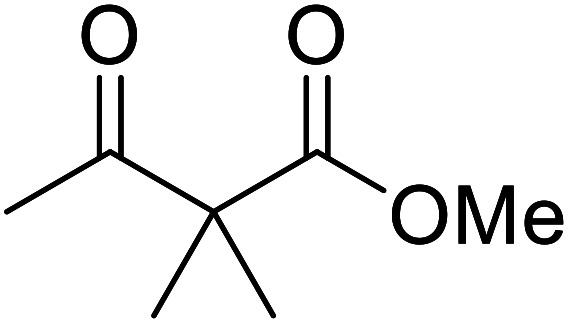			^*n*^Oct	23	93

a 2 mol% CsF was used.

## 11. Conclusion

The ability to selectively transesterify β-keto esters remains an important transformation in organic chemistry. As the market for alternative fuels, such as biodiesel, grows, the demand for robust and reliable transesterification methodologies will increase accordingly. Chemists are continually searching for higher yielding, milder and more environmentally benign conditions with broad substrate scope. In addition to traditional approaches based around metal catalysts, a variety of novel methodologies incorporating enzymes, clays or even catalyst-free conditions have been described in this review. Chemists can look forward to even more innovation in this field in the years ahead.

## Conflicts of interest

There are no conflicts to declare.

## Supplementary Material
